
JOURNAL INFORMATION
==============================
NLM Title Abbreviation: Innov Surg Sci
Journal ID: Innov Surg Sci
NLM Journal ID: 101708165
Journal ID: iss

Innovative Surgical Sciences

EISSN: 2364-7485
Publisher: De Gruyter

ARTICLE INFORMATION
==============================
PMCID: PMC10216891
DOI: 10.1515/iss-2023-9004
Article ID: iss-2023-9004
Article version: 1
Subjects: Article

Abstract DGAV

Publication date: 2023 Mar
Electronic publication date: 2023 Mar 13
Volume: 8
Issue: Suppl1
First page: 31
Last page: 122
Copyright: © the Author(s), published by De Gruyter
Copyright year: 2023
Copyright holder: the Author(s), published by De Gruyter
License: This work is licensed under the Creative Commons Attribution 4.0 International License.
License URL: https://creativecommons.org/licenses/by/4.0/

Page count: 92

==============================

**Sarcopenia in Bariatric Surgery - HIT BAR**
ID: 194

**Kategorie: DGAV - Bariatrische und metabolische Chirurgie**

Alida Finze[1]

[1]*Chirurgische Klinik, Universitätsmedizin Mannheim, Mannheim, Deutschland*

**Background:**
Bariatric surgery, especially sleeve gastrectomy (SG) and Roux-en-Y Gastric Bypass (RYGB), have emerged to be currently the most effective, most common (especially SG) and feasible surgical options for successful treatment of morbid obesity and comorbidities [1-3].
Although patients are advised to a high oral protein intake [4], there is data indicating different effect strengths on sarcopenia regarding oral protein intake [5]. In fact, some studies have shown that although patients adhere towards the currently recommended amount of oral protein intake after bariatric surgery, sarcopenia seems to develop in up to 50% of patients, especially after RYGB [6, 7].
Although data is very scarce, there are some indications that patients might profit from a supervised physical training in combination with elevated protein intake. Nevertheless, there are no clear recommendations for the type of exercise, the intensity, and goals within exercise for bariatric patients so far [8].
Furthermore, follow-up compliance in the bariatric patient group is known to be low, which is compliance is associated with comorbidities and vitamin deficiencies after surgery [9].
Considering the mostly young age of bariatric patients, there is a strong need for evaluation, if controlled physical activity can prevent sarcopenia and loss of strength whilst preserving a maximum of compliance regarding a possible exercise schedule.

**Materials and methods:**
We have initiated a prospective feasibility trial using high intensity interval training in patients receiving SG or RYGB at the surgical department of University Hospital Mannheim. Patients are being divided into three groups with different training protocols using high intensity interval bicycle training and one control group for a four-week training protocol. Primary outcome is compliance towards the training protocol. Gain in strength will be measured via bicycle ergometry and lactate tolerance.
Patients must only carry travel costs. The training is offered free of charge.

**Results:**
So far, compliance could be shown to be extremely low. Out of 153 recruited patients, 100 patients have been randomized. 39 have dropped out due to screening failure (prior to randomization) and 60 of the 100 randomized patients again dropped out. So far, 15 patients have completed the trial and 23 patients have completed training. Currently, 4 patients are undergoing training. Excluding screening failure, at least 60% of patients did not begin their training period.
Results regarding strength and lactate tolerance after a 4-week training period are still to be evaluated. We intend to give an overview over compliance in bariatric patients receiving controlled physical training after surgery. As a second goal, we wish to differentiate between different training protocols regarding gain of strength and compliance. For this purpose, an interim analysis is planned to be provided for the first 23 patients.

**Conclusion:**
A first overview shows very high incompliance in bariatric patients regarding controlled physical high-intensity interval training of at least 60% in the randomized patient group. This calls for the necessity of emphasizing the importance of physical activity after bariatric surgery and for keeping the threshold for physical activity as low as possible.

Open Access. © 2023 The Author(s), published by De Gruyter. This work is licensed under the Creative Commons Attribution 4.0 International License.

For the future, we intend to contribute to the development of recommendations for physical activity in patients who have undergone bariatric surgery for possible prevention of sarcopenia.

1. Wendy A Brown, L.K., Scott Shikora , Ronald Liem, Richard Welbourn, John Dixon, Edin Peter Walton, Robin Kinsman, *6th IFSO Global Registry Report 2021.* The IFSO Global Registry, 2021.
2. Henry Buchwald, M., PhD Yoav Avidor, MD Eugene Braunwald, MD Michael D. Jensen, MD Walter Pories, MD Kyle Fahrbach, PhD Karen Schoelles, MD, *Bariatric Surgery A Systematic Review and Meta-analysis.* JAMA, 2004. **292**(14).
3. Nguyen, N.T. and J.E. Varela, *Bariatric surgery for obesity and metabolic disorders: state of the art.* Nat Rev Gastroenterol Hepatol, 2017. **14**(3): p. 160-169.
4. Faria, S.L., et al., *Dietary protein intake and bariatric surgery patients: a review.* Obes Surg, 2011. **21**(11): p. 1798-805.
5. Ito, M.K., et al., *Effect of Protein Intake on the Protein Status and Lean Mass of Post-Bariatric Surgery Patients: a Systematic Review.* Obes Surg, 2017. **27**(2): p. 502-512.
6. Vassilev, G., et al., *Sarcopenia after Roux-en-Y Gastric Bypass: Detection by Skeletal Muscle Mass Index vs. Bioelectrical Impedance Analysis.* J Clin Med, 2022. **11**(6).
7. Voican, C.S., et al., *Predictive score of sarcopenia occurrence one year after bariatric surgery in severely obese patients.* PLoS One, 2018. **13**(5): p. e0197248.
8. Oppert, J.M., et al., *Resistance Training and Protein Supplementation Increase Strength After Bariatric Surgery: A Randomized Controlled Trial.* Obesity (Silver Spring), 2018. **26**(11): p. 1709-1720.
9. Reiber, B.M.M., et al., *Weight Loss Results and Compliance with Follow-up after Bariatric Surgery.* Obes Surg, 2021. **31**(8): p. 3606-3614.

**Preconditioning with Cilostazol reverses steatosis prior to major hepatectomy and improves liver regeneration.**
ID: 599

**Kategorie: DGAV - Bariatrische und metabolische Chirurgie**

Sebastian Holländer[1], Maximilian von Heesen[2], Gereon Gäbelein[1], Antonios Spiliotis[3], Matthias Laschke[4], Matthias Glanemann[1], Michael Menger[4]

[1]*Universitätsklinikum des Saarlandes, Klinik für Allgemeine Chirurgie, Viszeral-, Gefäß- und Kinderchirurgie, Homburg, Deutschland*, [2]*Göttingen Uniklinik, Klinik für Allgemein-, Viszeral- und Kinderchirurgie, Göttingen, Deutschland*, [3]*Charité Campus Virchow-Klinikum, Chirurgische Klinik, Berlin, Deutschland*, [4]*Universitätsklinikum des Saarlandes Institut für Klinisch-Experimentelle Chirurgie, Homburg/Saar, Deutschland*

**Background:**
The Non-Alcoholic-Fatty-Liver-Disease (NAFLD) is nowadays the most prevalent chronic liver disease and a rapidly growing epidemic worldwide. The reported prevalence of NAFLD, which varies across studies due to the use of different diagnostic methods and demographic factors, is estimated at 20-40% in the general population and at 70-95% in obese individuals.
Steatosis is a significant risk factor for liver failure after liver resection. The incidence of major complications in patients with NAFLD is higher than in patients with non-steatotic livers. Mortality rates following hepatectomy in patients with steatosis remain higher than 14%. Hepatectomies in patients with steatosis - even moderate - should be considered as high-risk procedures with increased rates of postoperative complications.
The phosphodiesterase-3 (PDE3)-inhibitor (cilostazol) has been associated with reduced levels of liver steatosis and inflammation in rats that had fed a choline-deficient, L-amino acid-defined (CDAA) diet or a high-fat high-calorie (HF/HC) diet. Cilostazol leads to increased hepatic blood perfusion, microcirculation, and liver regeneration. Furthermore, cilostazol reduces hepatocellular disintegration and inflammation after ischemia-reperfusion injury.
In the present study, given the increasing prevalence of NAFLD and its nutritional source, we investigated the effects of cilostazol on hepatocellular damage, liver regeneration, and inflammatory status after major hepatectomy in steatosis induced by high-fructose (high-FRC) diet in a rat model.

**Materials and methods:**
Mixed gender Sprague-Dawley rats (n = 64, body weight = 253 ± 3.6 g, 36 male) from own breeding were used for the experiments. Five weeks prior to surgery, the standard diet was replaced by a high-FRC diet. Animals were divided into the cilostazol group (n = 32, body weight = 231.7 ± 3.5 g, 9 males) and the control group (n = 32, body weight = 257.6 ± 5.6, 27 males).

*Experimental Protocol*
From the sixth week prior to the surgical procedure, all animals had access to a high-FRC diet. From the seventh preoperative day, the 32 animals from the cilostazol group received daily cilostazol oral (5 mg/kg body weight). Daily treatment with cilostazol at the same dose was continued during the postoperative period. The rest 32 animals (control group) received water without cilostazol.
Seven days following the treatment with cilostazol, all animals underwent a 70% liver resection (partial hepatectomy [PHX]). Hepatic blood flow and hepatic microcirculation were measured 60 minutes (POD 0), 24 hours (POD 1), 72 hours (POD 3), and 168 hours (POD 7) after PHX (n = 8 for each subgroup). After these in vivo measurements, 2 mL blood sample was taken from the inferior vena cava, and then the animals were sacrificed.

*Ultrasonic Flow Measurements*
An ultrasonic perivascular flow probe (0.5 V; Transonic Systems, Ithaca, NY) was placed around the hepatic artery. Another flow probe (1.5R; Transonic Systems, Ithaca, NY) was positioned around the portal vein. Hepatic arterial and portal venous blood flow were assessed simultaneously by use of a flow meter (T206 Animal Research Flowmeter; Transonic Systems). The detected blood flow was recorded for 10 minutes at each observation time point.

*Laser Doppler Flowmetry*
Microvascular perfusion of the liver parenchyma was evaluated with laser Doppler fluxmetry (PeriFlux System 500; Perimed, Inc., Hertfordshire, United Kingdom). For microvascular blood flow measurements, the fiberoptic probe of the laser Doppler flowmeter was placed on the surface of the right lateral liver lobe and fixed my means of a holder.

*Liver regeneration and Body Weight*
Prior to the surgical procedures, the body weight of the animals was measured. The remnant liver tissue was harvested at 1, 24, 72 and 168 hours after 70% hepatectomy, and the wet tissue weight was recorded.
After hepatectomy, the relative liver weight, indicating the regeneration rate, was expressed as percentage and was calculated according to the following formula: Regeneration rate = 100 * (remnant liver weight at given time point - $\frac{estimated\ total\ liver\ weight\ at\ operation - excised\ liver\ weight}{total\ liver\ weight}$).

*Histology and Immunohistochemistry*
In the histological analysis, hepatocellular steatosis, the vacuolization area, and the mean vacuole diameter were calculated. Cytoplasmic vacuolization of hepatocytes, indicating hepatocellular steatosis, was scored in 20 high-power fields (HPF) by a semiquantitative score from 0 to 4: (0) none, (1) minimal (<10% hepatocytes), (2) mild (10%-25% hepatocytes), (3) moderate (>25%-50% hepatocytes), and (4) severe (>50% hepatocytes). In addition, the vacuolization area and mean vacuole diameter was calculated using the software ImageJ. To calculate the mean vacuole diameter via ImageJ, horizontal lines were drawn at 10 different representative points of the sample image. The program then measured the diameters of the vacuoles among the lines and calculated the mean values.
In the immunohistochemical analysis, cell proliferation (PCNA), apoptotic cell death (cleaved caspase 3), and MPO-positive neutrophilic granulocytes were examined.

**Results:**
The weight of animals treated with cilostazol was significantly reduced compared to the untreated controls after completion of the 5-week high-FRC diet. Furthermore, 40% macrovesicular steatosis was found in the control group compared to 8% in the cilostazol group (POD 0, vacuolization area: 40.63 ± 3.2 vs 8 ± 2.39; $p < 0.001$).
The measured values for arterial and portal venous blood flow as well as for microvascular perfusion presented large heterogeneity, and only the mean arterial blood flow on day 3 in the animals treated with cilostazol was significantly reduced compared to the control group.
A lower liver enzyme release was found on POD 0, POD 1 and POD 7 in the animals treated with cilostazol compared to the control group. The difference on POD 1 was statistically significant (AST: 2.383 U/l vs 426 U/l; ALT: 1.278 U/l vs 198 U/l; $p < 0.001$). In the cilostazol group, significantly lower concentrations of ALP were found at POD 0, POD 1 ($p < 0.001$) and POD 7 ($p < 0.05$) compared to the control group.
In the immunohistochemical analysis, the rate of apoptosis was significantly reduced after pretreatment with cilostazol. In the control group, approximately three neutrophilic granulocytes (MPO-positive) per HPF were found on POD 0-7. In the cilostazol group, a significant reduction of the MPO-positive cells was recorded on all examination days.
The rate of PCNA-positive cells as a marker for cell division of the hepatocytes reached its maximum on POD 1 and decreased again on the following days (POD 3 and POD 7). Pretreatment with cilostazol led to a significant increase in PCNA-positive cells compared to the control group. This effect reached its maximum on POD 1 (proliferation index: cilostazol group vs controls in [%] = 20.85 ± 4.69 vs 10.02 ± 1.22; $p < 0.001$).
After PHX, there was a constant regeneration of the liver tissue, so that on POD 7 both groups showed a significant increase in tissue mass compared to POD 0. The regeneration in the control group remained stable between POD 3 and POD 7, and was presented significantly reduced compared to the cilostazol group on POD 7 (1.665 ± 0.218 vs 1.427 ± 0.129, $p < 0.05$).

**Conclusion:**
In our study, 40% macrovesicular steatosis developed in the animals of the control group, whereas only 10% steatosis was observed in the treatment group. In addition to the already known mechanisms of action of cilostazol in experimental non-steatotic models, we were able to show for the first time reduced inflammatory response (MPO), reduced liver cell damage (AST/ALT), improved liver regeneration (PCNA), and reduced cell death (caspase-3) in a steatotic major hepatectomy model after pretreatment with cilostazol.
Pretreatment with cilostazol represents an effective pharmacological preconditioning prior to major hepatectomy. It significantly reduces postoperative inflammation and hepatocellular damage, while improves liver regeneration by an “a priori” reduction of preexisting steatosis.

**Efficacy and Safety of Rivaroxaban for Postoperative Thromboprophylaxis in Patients after Bariatric Surgery: The BARIVA randomized clinical trial**
ID: 72

**Kategorie: DGAV - Bariatrische und metabolische Chirurgie**

Dino Kröll[1], Philipp Nett[1], Fabian Deichsel[2], Yves Borbely[1], Antonio Nocito[2], Jörg Zehenter[3], Urs Kessler[3], Yannik Fringeli[3], Lorenzo Alberio[4], Guido Stirnimann[1], Daniel Candinas[1]

[1]*Inselspital Bern, University Hospital and University of Bern, Department of Visceral Surgery and Medicine, Inselspital, Bern, Schweiz*, [2]*Cantonal Hospital of Baden, Department of Surgery, Schweiz*, [3]*Hirslanden Clinic Beau-Site, Berne, Switzerland, Department of Visceral Surgery,, Bern, Schweiz*, [4]*Centre Hospitalier Universitaire Vaudois (CHUV), University of Lausanne (UNIL), CH 1011 Lausanne, Switzerland, Division of Haematology and Central Haematology Laboratory, Lausanne, Schweiz*

**Background**
Venous thromboembolism (VTE) is a leading cause of morbidity and mortality after bariatric surgery. Studies on thromboprophylaxis with direct oral anticoagulants investigating clinical endpoints and duration of prophylaxis are lacking. The objective of this study was to assess efficacy and safety of prophylactic rivaroxaban once daily after bariatric surgery.

**Methods**
This randomized, assessor blinded clinical phase 2 multicenter trial was conducted from July 2018 through June 2021. Patients were randomized one day after bariatric surgery to 10 mg oral rivaroxaban for either 7 (short prophylaxis) or 28 days (extended prophylaxis). Primary efficacy outcome was the composite of deep-vein thrombosis (symptomatic or asymptomatic) and pulmonary embolism within 28 days after bariatric surgery. Main safety outcomes included major bleeding, clinically relevant non-major bleeding and mortality.

**Results**
272 patients were randomized to either 7 (n=135) or 28 days (n=137) of VTE prophylaxis. Only one thromboembolic event (0.39%) occurred (asymptomatic thrombosis in a Sleeve Gastrectomy patient with extended prophylaxis). The rates of major bleeding and clinically relevant non-major bleeding in the short prophylaxis group was 0.75%, each. In the 28-day arm, rates of major bleeding and clinically relevant non-major bleeding were 0.74% and 1.48%, respectively. Bleeding rates and adverse events were not different between the two treatment groups nor in any subgroup investigated (Sleeve Gastrectomy, Roux-en-Y gastric bypass, revisional surgery).

**Conclusions**
A prophylactic 10 mg once-daily dose of rivaroxaban was effective and safe in the early postoperative phase after bariatric surgery in both the short and the extended prophylaxis group.

**Santoro- Transit Bipartition after Sleeve Gastrectomy: preliminary results and Comparison to Sleeve Ileal Bipartition with Braun's jejunojejunostomy.**
ID: 816

**Kategorie: DGAV - Bariatrische und metabolische Chirurgie**

Ashwini Rao[1], Vasiliki Christogianni[1], Radostina Dukovska[1], Osama El Zaidi[1], Martin Büsing[1]

[1]*45657, Klinikum Vest, Knappschaftskrankenhaus, Recklinghausen, Deutschland*

**Background:**
Worldwide obesity has nearly tripled since 1975, with over 39% adults suffering from being overweight and around 13% adults suffering from obesity.[1] Bariatric surgery- in terms of restrictive or malabsorptive procedures- has become the need of the hour.
Sleeve gastrectomy (SG) has established itself as the treatment of choice in the treatment of morbid obesity [4]. Failure of effective weight loss (EWL<50%) and de novo reflux are two common indications for a revisional operation. Bypass surgery like Roux-en-Y or OAGB are usually performed to combat the aforementioned problems.
Transit bipartition procedures promise to counterbalance the effect of food with high glycemic index by hormonally hypostimulating the proximal gut and hyperstimulting the distal gut, providing an effective weight loss and in comparison to minimize the risk of extreme malabsorption. Furthermore, the additional stomach outlet can be help reduce the GERD symptoms, by reducing the pressure in the sleeve. Thus, Sleeve Ileal Bipartition with Braun´s jejujejunostomy (SASI with BJJ) and Santoro Bipartition are the procedures of choice performed in our clinic in cases of weight regain/inadequate weight loss and GERD symptoms after SG. Purpose of this study is to present our preliminary results of Santoro Bipartition as revisional operation after SG and compare our findings with those of SASI Bipartition with BJJ

**Materials and methods:**
We retrospectively analyzed data of patients undergoing Santoro Bipartition after SG in our center, from July 2020 until April 2022. From the 72 patients, 9 patients were excluded from the study (n= 1, was a single setting Sleeve and Santoro surgery; and n=8 were revision surgeries due to persistent GERD after SASI Bipartition). Due to loss to Follow- up, further 19 were not enclosed. Patients with s/p SG and persistent GERD (Grade B or above Reflux Esophagitis-Classification of Savary-Millers, Reflux Index Score 5 or more, presence of Bile Reflux in the upper endoscopy) and/or Weight regain were considered as indication for the surgery. 58 patients of the same demographics, undergoing SASI Bipartition with BJJ were at the end compared to the Santoro group.
<u>Additional Information:</u> The patient excluded from the list were nevertheless observed for any perioperative complications till the date of discharge that could have been relevant to the study. Since those data dint change the mean surgical duration or have any significant deviation from the presented data with regards to intraoperative complications, have not been included.

**Results:**
Of the analyzed 72 cases, 44 patients consisting of 8 men and 36 women with a mean age of 45+/-9.5 years were considered. On an average, patients underwent revisional surgery 48 months after the initial procedure.

*Perioperative complications observed in the first 30 days.* In 2 cases, a laparotomy due to massive adhesions was necessary. 10 patients underwent an additional hiatoplasty and in further 10 cases, an extended laparoscopic adhesiolysis needed to be performed. 2 patients developed Pneumothorax, of which one was treated conservatively and the second with a drain. In the 30 day follow up period, 13 patients were readmitted. In 5 cases a revisional operation was performed due to symptoms of mechanical ileus, caused by internal hernia (n=3) or adhesions (n=2). Other readmission causes were epigastric pain or fever (n=8). Upper endoscopy was performed in all of patients presenting with symptoms of epigastric pain or dysphagia revealing a gastritis and duodenitis in 5 cases. 2 patients developed pneumonia and were treated with oral antibiotics.
In the 30 day follow up patients were satisfied with the weight loss (mean BMI 34.97+/-4.6 $kg/m^2$) and 37 patients agreed to having relatively less GERD symptoms.
At the 1year follow up 25 patients are satisfied with the
Surgery, 7 patients weren't satisfied with the outcome (n=5, were converted to Roux-en-y Bypass)

*Comparitive study to SASI Bipartition:*

| | SASI | Santoro |
|---|---|---|
| OP Duration | 123+/-36 | 114+/-23 |
| Hospital stay | 3-4 days | 2-4 days |
| Initial BMI | 49.88+/-8.52 | 48.3+/-5.3 |
| Lowest BMI | 32.7+/-6.05 | 32.66+/-5.25 |
| BMI perioperative | 37.61+/-7.08 | 37.42+/-5 |
| BMI s/p 1 Month | 35.83+/-6.2 | 34.97+/-4.6 |
| BMI 1 y | 33.63+/-10.57 | 32,26+/-5.3 |
| TBWL 1y | 33,36+/-10.574 | 31,62+/-7.37 |
| EWL 1y | 70,38+/-17.75 | 68,12+/-16.25 |
| EeWL 1y | 45.46+/-24.75 | 52.02+/-24.41 |

**Conclusion:**
Santoro Transit Bipartition after SG so far gives promising results, with satisfactory weight loss and clear improvement of GERD symptoms compared to standard Bypass procedures. A surgery which resembles a Roux-en-Y procedure without the biggest drawback of its irreversibility and restricted endoscopic treatments could provide an alternative surgical treatment in the future to post- sleeve gastrectomy patients.
The present study will be continued to better understand the long term outcomes.

**360° Gastroplication with the remnant stomach- An option for late dumping syndrome after Roux-en-y gastric bypass? - a small case series**
ID: 822

**Kategorie: DGAV - Bariatrische und metabolische Chirurgie**

Vasiliki Christogianni[1], Ashwini Rao[1], Radostina Dukovska[1], Marlon de la Cruz[1], Martin Büsing[1]

[1]*Klinikum Vest, Recklinghausen, Deutschland*

**Background:**
Dumping syndrome after Roux–en-y gastric bypass (RNGB) is common and usually, can be treated with diatery modifications. In cases of persistent hypoglycemia, acarbose or somatostatine analogues may be considered. Though rare, some patients suffer from uncontrolled neuroglycopenic symptoms, resistant to conservative therapy. Procedures which delay the passage of ingested food or reverse the effect of reactive hyperinsulinemia have been proposed. We present a small case series of five patients who underwent a 360° gastroplication with the remnant stomach in order to treat late dumping after RYGB.

**Materials and methods:**
A retrospective analysis of patients who underwent a 360° Gastroplication to treat the symptoms of dumping syndrome(n=5, f=5). Three patients underwent a primary RYGB. Two were treated initially with a sleeve gastrectomy followed by a RNGB as revisional procedure to enhance weight loss. Mean BMI prior to bariatric surgery was 44.9 kg/m$^2$ and at the time of the revisional operation 27.8 kg/m$^2$. In all patients a 360° gastroplication with the remnant stomach was performed in the site of the anastomosis. One patient with a preexisting penetrating minimizer ring underwent additionally a revision of the anastomosis. Patients suffering from neuroglycopenic symptoms for more than 1 year and resistant to conservative therapy were selected for the procedure. In the Follow up, a subjective improvement of hypoglycemic n neuroglycopenic symptoms were checked, for assessing the improvement.

**Results:**
In the one month, 6months and 12months follow up all patients showed complete resolution of symptoms.

**Conclusion:**
Modified gastroplication after RYGB seems to delay the transit of ingested food, resulting in better control of hypoglycemia in patients suffering from severe dumping.

### Elaboration of a metabolic-bariatric colorectal carcinoma mouse model (RyGB-CRC model)

ID: 881

**Kategorie: DGAV - Bariatrische und metabolische Chirurgie**

Claudia Läßle[1], Bernhard Mauerer[2], Lisa Marx[2], Christopher Berlin[1], Gabriel Seifert[1], Jodok Fink[1], Stefan Fichtner-Feigl[1], Goran Marjanovic[1], Rebecca Kesselring[2]

[1]*Klinik für Allgemein- und Viszeralchirurgie, Universitätsklinikum Freiburg, Freiburg, Deutschland*,
[2]*Zentrum für Translationale Zellforschung, Universitätsklinikum Freiburg, Freiburg, Deutschland*

**Background:**
Colorectal carcinoma (CRC) is the seventh leading cause of death in the Western world. Obesity is a major risk factor for CRC. Clinically, the first positive effects after metabolic-bariatric surgery on CRC carcinogenesis are becoming apparent. Basic science has not yet investigated this impressive effect.

**Materials and methods:**
To investigate these impressive results, we developed a metabolic-bariatric mouse model in form of a classic Roux-Y-gastric bypass (RyGB) and implanted in a second step CRC-organoids under the subserosa of the cecum of the mice.

**Results:**
Mice with a RyGB developed significantly smaller CRC tumors than the control group without RyGB surgery under high fat diet and under normal chow. In addition, the RyGB group showed no hepatic metastasis while the HFD and NC group developed multiple liver metastases.

**Conclusion:**
A RyGB-CRC mouse model was successfully developed. The clinically observed protective effect of RyGB surgery regarding carcinogenesis and invasiveness of CRC was successfully demonstrated. Further investigations are still needed.

### VACStent applicability and safety – pooled results from 80 implantations in the upper gastrointestinal tract

ID: 228

**Kategorie: DGAV - Endoskopie und Sonographie**

Jonas Lange[1], Georg Kähler[2], Dörte Wichmann[3], Jörn Bernhardt[4], Ulrich Hügle[1], Judith Knievel[5], Dirk R. Bulian[1], Claus Ferdinand Eisenberger[1], Markus Maria Heiss[1]

[1]*Kliniken der Stadt Köln gGmbH, Köln, Deutschland*, [2]*Universitätsklinik Mannheim, Mannheim, Deutschland*, [3]*UKT Tübingen, Tübingen, Deutschland*, [4]*Klinikum Südstadt Rostock, Rostock, Deutschland*, [5]*Universität Witten/Herdecke, Witten, Deutschland*

**Background:**
The VACStent consists of a fully covered nitinol stent surrounded by an open pore polyurethane sponge cylinder.  A suction catheter creates a suction in the sponge which fixes the VACStent to the intestinal lumen and promotes wound healing by the endoscopic vacuum effect (EVT). To date, initial results are available for various indications, which are evaluated together in this analysis to assess applicability and safety.
The data from three different study collectives should be evaluated together with regard to the criteria of applicability and safety in order to enable a better assessment.

**Materials and methods:**
The data pool includes data from the VACStent registry and the VACStent trial. In the prospective registry, 39 VACStent implantations have been documented so far, 30 for leakage and 9 intraoperative applications for preemptive therapy of esophageal anastomosis. The prospective VACStent trial includes 41 implantations for leaks of various causes, mostly as anastomotic insufficiency after esophagectomy. All aspects of implantation (the positioning, the release, the positional stability, the suction, the drainage function), the VACStent removal (the migration, the ingrowth of the sponge cylinder) and the possible local sequelae (the bleeding, the ulceration, the perforation) were recorded and evaluated.

**Results:**
A total of 80 VACStents were implanted in 42 patients. No complication, lesion, perforation, or bleeding was seen during application and release. In one case, the VACStent was released too distally, but this could be corrected and repositioned. Transnasal suction catheter expulsion was successful in all cases, as was suction build-up and drainage by vacuum pump (suction varied between 75-125 mmHg). The average duration of VACStent placement was 4.6 days (3-8) and there was no case of relevant dislocation. Removal was possible in all cases; sponge loss occurred once. However, this could be completely recovered and removed. During loosening and extraction of the sponge, documented minor mucosal bleeding occurred rarely (5%: 4/80), and local hemostasis was necessary once. No perforation or major pressure ulcer occurred. Overall, the healing rate was 81% (34/42), although the collectives were clearly inhomogeneous in underlying diseases and pretreatments.

**Conclusion:**
The data demonstrate that the VACStent can be easily delivered to the upper GI tract in most cases and is well positioned. Release is easy and has a low potential for dislocation. The VACStent is well tolerated and the drainage function of the sponge cylinder produces very good positional stability and direct wound closure due to suction. The application is less complicated and the associated EVT effect effectively promotes wound healing. Further studies should clarify the value in different indications.

### Endoscopic ultrasonography(EUS)-guided therapy of cholangiolithiasis (CAL) in surgically altered anatomy of the upper GI tract - a unicenter case study describing real-world data

ID: 646

**Kategorie: DGAV - Endoskopie und Sonographie**

Frank Füldner[1], Frank Meyer[2], Uwe Will[1]

[1]*Dept. of Gastroenterology, Hepatology and General Internal Medicine, Municipal Hospital ("SRH Wald-Klinikum Gera gGmbH"), Gera, Deutschland*, [2]*Dept. of General, Abdominal, Vascular & Transplant Surgery, University Hospital of Magdeburg, Magdeburg, Deutschland*

**Background:**
For the treatment of cholangiolithiasis, ERCP can be considered the gold standard. In cases of surgically altered anatomy of the upper GI tract with or without a biliodigestive anastomosis, conventional ERCP using a duodenoscope can often be frustrating and unsuccessful. Therapeutic alternatives are ERCP using balloon-enteroscope, PTCD or even surgical intervention. Due to long-term experiences in EUS-guided techniques for drainage of the biliary system, the novel and favorable option of an endoscopic ultrasonography (EUS)-guided antegrade or even retrograde gall stone extraction via a transhepatic route.

**Materials and methods:**
All consecutive patients with cholangiolithiasis and surgically altered anatomy of the upper GI tract with and without previous attempts of an ERCP were enrolled in this unicenter case study over a defined period of time and were characterized with regard to the technical and clinical success of this approach.

**Results:**
From 2004 to 03/2020 overall 449 patients underwent EUS-guided cholangiodrainage in the reporting center. Among them there were 37 patients with cholangiolithiasis. In 8 of these 37 patients, gall stone extraction was achieved using EUS-ERCP rendezvous technique who were not included in this study since there was no surgically altered anatomy of the upper GI tract. In 13 of the remaining 29 subjects (45 %), there was a failure of previous attempts to reach the papilla of Vater od biliodigestive anastomosis using balloon-enteroscopy-guided ERCP. EUS-guided access to the biliary system was achieved in all 29 patients. Stone extraction was performed in 26 individuals (90 %) by means of antegrade push-technique after balloon dilatation of the papilla of Vater and biliodigestive anastomosis, respectively, before. In 11/29 cases (42 %), double pigtail prostheses were subsequently placed to track papilla of Vater / biliodigestive anastomosis ("ring drainage"), which were removed with gastroscopy three months later after previous ultrasound- and lab parameter-based follow-up control. In two patients (7 %), gall stones were extracted via a retrograde route using a transhepatic access site; in one patient (3 %), stone were removed by means of a combined antegrade and retrograde technique. In two subjects (7 %), cholangioscopy with electrohydraulic lithotripsy was used. Technical as well as clinical success rate was 100 % (29 of 29 patients). Re-interventions became necessary in 6/29 cases (21 %), complications occurred in 6 individuals (21 %).

**Conclusion:**
EUS-guided stone extraction in antegrade or retrograde technique for patients with surgically altered anatomy of the upper GI tract can be considered a favorable and safe but challenging approach of interventional endoscopy/EUS. It can provide a high technical and clinical success and low complication rates. In addition, it has the potential to substitute the time-consuming balloon-enteroscopy-guided ERCP as well as, in particular, PTCD and, thus, secundary and tertiary therapeutic alternatives.

**Comparison of postoperative hyperlipasemia and hyperamylasemia in predicting pancreatic fistula after pancreatic resections**
ID: 104

**Kategorie: DGAV - Hepato-pankreato-biliäre Chirurgie**

Ughur Aghamaliyev[1], Ganildo Cepele[2], Claudius Kessler[1], Felix Hofmann[1], Jens Werner[1], Jan G. D'haese[1], Maximilian Weniger[1]

[1]*Klinikum Großhadern, Klinik für Allgemein-, Viszeral- und Transplantationschirurgie, München, Deutschland*, [1]*Klinikum Großhadern, Klinik für Allgemein-, Viszeral- und Transplantationschirurgie, München, Deutschland*

**Background:**
Postoperative pancreatic fistula (POPF) is the most significant source of postoperative morbidity after pancreatic resection and contributes to reduced short and long-term survival. Hence, multiple efforts have been undertaken to predict POPF and identify patients at risk. Recently, emerging data indicate that postpancreatectomy acute pancreatitis (PPAP) might be a critical factor in POPF pathophysiology. Although serum amylase activity has been shown to predict PPAP and POPF, there are currently few reports to predict POPF based on serum lipase activity. Particularly, studies including both amylase and lipase activity are lacking. As a result, serum lipase has not been taken into consideration in the new classification of PPAP by International Study Group for Pancreatic Surgery (ISGPS). Accordingly, we sought to clarify whether serum lipase can predict POPF in a patient cohort after pancreatic resections.

**Materials and methods:**
Patients with pancreatic resection from January 2019 to December 2021 at our hospital were identified retrospectively. Clinical data were collected including age, gender, BMI, diagnosis, surgical procedure, estimated intraoperative blood lost, postoperative pancreatic fistula (POPF), serum amylase and lipase levels on POD0, POD1, POD2 and POD3.

**Results:**
A total of 328 patients were included in this study. Of these, 102 patients had both serum amylase and lipase concentrations measured on POD0, 210 on POD1, 185 on POD2, 154 on POD3. Serum levels of amylase and lipase on POD0, POD1, POD2, and POD3 were both significantly higher in patients with CR-POPF ($P<0.001$). Receiver operating characteristic (ROC) analyses showed improved diagnostic accuracy for POD0, POD2, POD3 serum lipase compared with serum amylase in predicting POPF (POD0: AUC: 0.74 vs. 0.72, $p <0.001$; POD1: AUC: 0,69 vs 0,69, $p <0.001$; POD2: AUC: 0.70 vs. 0.64, $p <0.001$; POD3: AUC: 0.73 vs. 0.66, $p <0.001$). Moreover, Pearson correlation analysis showed a strong positive correlation between postoperative serum amylase and lipase levels on POD0, POD1, POD2 and weaker correlation on POD3 (POD0: $R=0.87$, $p<0.0001$; POD1: $R=0.93$, $P<0.0001$; POD2: $R=0.85$, $p<0.0001$; POD3: $R=0.67$, $p<0.0001$). Furthermore, the univariate analysis showed that serum lipase on POD0, POD1, POD2, POD3 and serum amylase on POD0 and POD1 were risk factors for CR-POPF. The multivariate analysis demonstrated that evidence of elevated serum lipase level on POD 0 was an independent risk factor for CR-POPF after pancreatic resections.

**Conclusion:**
In our single-center retrospective study, postoperative hyperlipasemia was more valuable than hyperamylasemia for predicting CR-POPF after pancreatic resections.

**Conventional partial pancreatoduodenectomy versus an uncinate first, extended partial pancreatoduodenectomy approach for the resection of pancreatic head cancer – the randomized, controlled PancER pilot trial**
ID: 122

**Kategorie: DGAV - Hepato-pankreato-biliäre Chirurgie**

Patrick Heger[1], Thilo Hackert[2], Manuel Feißt[3], Matthias Gailda[4], Christina Klose[3], Phillip Knebel[2], Rosa Klotz[2], Colette Dörr-Harim[1], Christoph Michalski[1], Markus Büchler[2], André Mihaljevic[1]

[1]*Allgemein- und Viszeralchirurgie, Uniklinik Ulm, Ulm, Deutschland*, [2]*Allgemein-, Viszeral- und Transplantationschirurgie, Uniklinik Heidelberg, Heidelberg, Deutschland*, [3]*Institut für Medizinische Biometry (IMBI), Heidelberg,*, [4]*Institut für Pathologie, Universität Mainz, Mainz, Deutschland*

**Background:**
Pancreatic cancer is one of the leading causes of cancer related death. Even after curative resection by pancreatoduodenectomy (PD), more than 45% of patients suffer from recurrence. The rate of complete microscopic tumor clearance (R0) is associated with the probability of local recurrence and overall survival. A potential intervention to improve the rate of R0 resections is a more radial resection along the superior mesenteric artery (SMA), however randomized data comparing the potential risks and benefits of such an approach are lacking. Therefore, we conducted the randomized, controlled PancER pilot trial to evaluate feasibility and safety of an extended PD compared to conventional PD.

**Materials and methods:**
Patients have been randomized to extended PD consisting of a modified Kocher maneuver with partial resection of the prerenal fascia, an uncinate first-approach and a systematic mesopancreatic dissection along the SMA equivalent to level III dissection according to Inoue et al., and conventional PD, that was performed including a standard Kocher maneuver, a cranio-caudal resection and dissection of the SMA according to guidelines (Inoue level I and II). The primary endpoint, rate of R0 resections, and other perioperative outcomes have been evaluated.

**Results:**
A total of 50 patients were randomly assigned to extended PD (n=24) and conventional PD (n=26). We decided to prematurely terminate the PancER trial as another more radical and promising intervention was developed (TRIANGLE operation) and recruitment of the trial was behind expectations. Of the 50 patients randomized, 37 patients could be followed-up and were analysed. We found 6% more R0 resections in the extended PD group (26.3% compared to 20.0% in the conventional PD group), which did not show to be statistically significant (p=0.79). Patients after extended PD had significantly higher rates of diarrhea (31.8% grade 1 and 22.7% grade 2 diarrhea vs. 6.7% grade 1 and no grade 2 diarrhea in the conventional PD group; p=0.01). Other perioperative outcomes, including the operation time, were comparable.

**Conclusion:**
The PancER pilot trial shows that extended PD is a safe and feasible procedure with comparable perioperative morbidity, but a significantly elevated rate of diarrhea. However, the improvement in the R0-resection rate was below expectation. Therefore, we developed a more radical resection technique involving not only the SMA, but also the celiac and hepatic artery (TRIANGLE operation), which is currently being investigated in a randomized controlled multicentre study. Results of the PancER pilot trial served to improve this subsequent trial.

### Liver Regeneration after two-stage liver transplantation is more effective than after other preconditioning procedures in colorectal liver metastases

ID: 176

**Kategorie: DGAV - Hepato-pankreato-biliäre Chirurgie**

Aladdin Ali Deeb[1], Falk Rauchfuss[2], Utz Settmacher[2]

[1]*Universitätsklinikum Jena, Klinik für Allgemein-, Viszeral- und Gefäßchirurgie, Jena, Deutschland*,
[2]*Universitätsklinikum Jena, Klinik für Allgemein-, Viszeral- und Gefäßchirurgie, Jena,*

**Background:**
Induction of liver regeneration represents an option to expand the resectability in patients with expected small future liver remnant (FLR). The aim of this cohort-study is to compare the liver regeneration between different surgical procedures, including novel procedures such as two-stage living donor liver transplantation using a small for size grafts.

**Materials and methods:**
Forty-three patients with colorectal liver metastases were included between 2004 and 2020. These underwent one of the following three procedures: portal vein embolization (PVE), associated liver partition with portal vein ligation for staged hepatectomy (ALPPS), and living donor two-stage liver transplantation (LT). The volume gain of the future liver remnant was analyzed in comparison between the three mentioned procedures.

**Results:**
The type of surgery performed had a significant correlation with liver regeneration with a strong effect to the benefit of ALPPS and liver transplantation, respectively ($r = 0.6$, $P = 0.00003$). The type of surgery was the only independent co-factor in the multiple regression, which showed a significant influence on FLR-increase favouring two-stage transplantation compared to the other two related procedures ($ß = 0.12$, $T = 3.9$, $P = 0.0004$). The histological and immunohistochemical studies also showed a clear advantage of proliferation to the benefits of two-stage liver transplantation compared with ALPPS.

**Conclusion:**
Two-stage liver transplantation using small for size grafts induces better FLR-increase than portal vein embolization or ALPPS in patients with colorectal liver metastases.

**The revised R-status is an independent predictor of post-resection survival in pancreatic cancer after neoadjuvant treatment**
ID: 206

**Kategorie: DGAV - Hepato-pankreato-biliäre Chirurgie**

Carl-Stephan Leonhardt[1], Dietmar Pils[1], Motaz Qadan[2], Gerd Jomrich[1], Ulla Klaiber[1], Klaus Sahora[1], Martin Schindl[1], Andrew Warshaw[2], Keith Lillemoe[2], Oliver Strobel[1], Carlos Fernandez del-Castillo[2], Thomas Hank[1,2]

[1]*Klinische Abteilung für Viszeralchirurgie, Universitätsklinik für Allgemeinchirurgie, Medizinische Universität Wien, Wien, Österreich*, [2]*Department of Surgery, Massachusetts General Hospital, Harvard Medical School, Boston, United States*

**Background:**
The revised R-status is an independent prognostic factor in upfront resected pancreatic ductal adenocarcinoma (PDAC). However, the prognostic impact of a 1mm margin clearance after neoadjuvant treatment (NAT) remains controversial. This study aims to investigate the oncological outcomes of patients with PDAC who have a R0 or R1 resection based on the revised R-status (1mm) definition after neoadjuvant therapy (NAT).

**Materials and methods:**
Patients undergoing pancreatectomy following NAT for PDAC between 2010 and 2021 were identified from two prospectively maintained databases. Clinicopathological and survival data were analyzed. Primary outcomes were overall survival (OS), recurrence-free survival (RFS) and pattern of recurrence in association with R0>1mm and R1≤1mm resections.

**Results:**
Three hundred fifty-seven patients with PDAC were included after NAT with subsequent pancreatic resection. Two hundred eight patients (58.1%) received FOLFIRINOX, 42 (11.7%) had gemcitabine-based regimens, 104 had other regimens, and 292 (82.7%) individuals received additional radiotherapy. R0 resection was achieved in 272 (76.0%) patients and 86 (24.0%) had R1 resection. Median OS after R0 was 41.0 months (m) compared to 20.6 m after R1 resection (p=0.002) and median RFS after R0 was 17.5 m vs 9.4 m after R1 resection (p<0.0001). R1 resections were significantly associated local but not distant recurrence (p=0.0005). In addition, R-status was confirmed as independent predictor for OS (R1: HR 1.56, 95%-CI 1.07-2.26) and RFS (R1: HR 1.52; 95%-CI 1.14-2.0).

**Conclusion:**
The revised R status is an independent predictor after NAT for postresection survival in PDAC. This association was confirmed in subgroup analysis of patients who received adjuvant chemotherapy. Achieving R0 resections should be a primary goal in the surgical treatment of PDAC patients in the era of NAT.

**Tumor Burden Score predicts 1-year overall survival after curative resection of colorectal liver metastases.**
ID: 226

**Kategorie: DGAV - Hepato-pankreato-biliäre Chirurgie**

Iakovos Amygdalos[1], Alexandros Chrysos[1], Katharina Jöchle[1], Jan Bednarsch[1], Lea Hitpass[2], Gustav Anton Müller-Franzes[2], Daniel Truhn[2], Philipp Bruners[2], Christiane Kuhl[2], Tom Florian Ulmer[1], Ulf Neumann[1], Andreas Lambertz[1], Sven Arke Lang[1]

[1]*University Hospital RWTH Aachen, Department of General, Visceral and Transplantation Surgery, Aachen, Deutschland*, [2]*University Hospital RWTH Aachen, Department of Diagnostic and Interventional Radiology, Aachen, Deutschland*

**Background:**
Colorectal cancer is the third most common cancer type worldwide. Distant metastasis, particularly liver metastasis, is of outmost importance in terms of prognosis and multiple scores have been developed to predict survival in patients with colorectal liver metastases (CRLM). This study examines the capacity of the Tumor Burden Score (TBS) to predict 1-year overall survival (OS) in patients undergoing curative resection of CRLM at a German university hospital.

**Materials and methods:**
Patients undergoing curative liver resection for CRLM between 2010-2021 at the University Hospital RWTH Aachen were eligible for this retrospective study. Patients with recurrent metastases and in-hospital deaths were excluded. TBS was calculated using the number of liver metastases and diameter of the largest lesion, as extracted from preoperative imaging. A receiver-operating curve (ROC) was used to define a TBS cut-off value for prediction of 1-year OS. Differences between patients with TBS below and above the cut-off were examined with Kaplan-Meier and Cox regression analyses, and the log-rank test.

**Results:**
A total of 528 patients were included. ROC analysis showed an Area Under the Curve (AUC) of 0.71 for prediction of 1-year OS, with a cut-off value for TBS of 5.0 (Youden Index = 0.360). Patients with TBS≥5.0 showed a statistically significant reduction in 1-year OS (cumulative survival 86% vs. 97%, $p<0.001$). The Hazard Ratio for TBS≥5.0 regarding 1-year OS was 4.733 (95%CI 2.089-10.724, $p<0.001$).

**Conclusion:**
A TBS of 5.0 or more is associated with a reduced 1-year OS after curative resection of CRLM. Careful preoperative patient selection, closer postoperative follow-up and aggressive perioperative chemotherapy regimens may improve outcomes in these patients.

**Survival after combined resection and ablation of multiple colorectal liver metastases is not inferior to that after resection alone.**
ID: 235

**Kategorie: DGAV - Hepato-pankreato-biliäre Chirurgie**

Iakovos Amygdalos[1], Katharina Jöchle[1], Alexandros Chrysos[1], Felix Schmidt[1], Gerrit Josephs[1], Lea Hitpass[2], Jan Bednarsch[1], Gustav Anton Müller-Franzes[2], Daniel Truhn[2], Philipp Bruners[2], Tom Florian Ulmer[1], Andreas Lambertz[1], Ulf Neumann[1], Sven Arke Lang[1]

[1]*University Hospital RWTH Aachen, Department of General, Visceral and Transplantation Surgery, Aachen, Deutschland*, [2]*University Hospital RWTH Aachen, Department of Diagnostic and Interventional Radiology, Aachen, Deutschland*

**Background:**
Colorectal liver metastases (CRLM) are the predominant factor limiting survival in patients with colorectal cancer. Especially in patients with multiple CRLM, a combination of treatment strategies may be necessary to achieve total tumor elimination and/or prolong survival. This study examines the efficacy of liver resection combined with ablation, in the treatment of patients with four or more CRLM, in terms of survival.

**Materials and methods:**
Patients undergoing curative liver resection for CRLM between 2010-2021 at the University Hospital RWTH Aachen, with four or more liver lesions in preoperative imaging, were eligible for this retrospective study. Patients with recurrent metastases and early deaths were excluded. The number of liver lesions was extracted from preoperative imaging. Differences in overall- (OS) and recurrence-free-survival (RFS) between patients treated with resection+ablation vs. those with resection only, were examined with Kaplan-Meier and Cox regression analyses, and the log-rank test.

**Results:**
A total of 207 patients were included, of which 55 (27%) were treated with resection+ablation and 152 (73%) with resection only. Kaplan-Meier and log-rank test analyses showed no statistically significant differences in median OS (36 months for resection+ablation vs. 29 months for resection only, $p$=0.411) or median RFS (6 months for resection+ablation vs. 7 months for resection only, $p$=0.932). Cox regression analysis showed a hazard ratio of 1.195 ($p$=0.417) for OS and 1.015 ($p$=0.935) for RFS, for the resection group.

**Conclusion:**
For patients with multifocal CRLM, ablation as an adjunct to resection is a safe and viable option, which may preserve more liver parenchyma and allow for re-resections of recurrences. Further studies are necessary to elucidate the role of ablation as an adjunct or standalone treatment in these patients.

**Perioperative Drug Treatment in Pancreatectomy**
ID: 326

**Kategorie: DGAV - Hepato-pankreato-biliäre Chirurgie**

Ingmar F Rompen[1], Daniela Merz[1], Henrik Nienhüser[1], Thomas Pausch[1], Rosa Klotz[1], Pascal Probst[1,2]

[1]*Klinik für Allgemein-, Viszeral- und Transplantationschirurgie, Universitätsklinikum Heidelberg, Heidelberg, Deutschland*, [2]*Kantonsspital Thurgau, Allgemeinchirurgie, Frauenfeld, Schweiz*

**Background:**
Pancreatic resections for malignant or benign diseases are associated with major morbidity and changes in physiology. To reduce perioperative complications and enhance recovery, many perioperative medical managements have been introduced. The aim of this study was to give an evidence-based overview on best perioperative drug treatment.

**Materials and methods:**
The electronic bibliographic databases Medline, Embase, CENTRAL and Web of Science were systematically searched for randomized controlled trials evaluating perioperative drug treatments in pancreatectomy. The investigated drugs were somatostatin analogues, steroids, pancreatic enzymes replacement therapy (PERT), prokinetic therapy, antidiabetic drugs, and proton pump inhibitors (PPI). Targeted outcomes in each drug category were meta-analysed.

**Results:**
A total of 45 trials were included. The analysis of somatostatin analogues showed a significantly lower incidence of POPF in the somatostatin group compared to the control group (12.0% vs. 18.5%, OR 0.58, 95%CI; 0.45-0.74, p<0.01, I2=20%). Overall complications, however, failed to show a significant difference (OR 0.76, 95%CI; 0.56-1.02, p=0.07, I2=49%). The comparison of glucocorticoids versus placebo showed significantly less grade B and C fistulas in the glucocorticoid group (8.9% vs. 31.25%, OR 0.22, 95%CI; 0.07-0.77, p=0.02, I2=3%). There was no significant difference in delayed gastric emptying when erythromycin was compared to placebo (OR 0.33, 95%CI; 0.08-1.30, p=0.113).

**Conclusion:**
This is the first systematic overview on perioperative drug treatment in pancreatic surgery. In some interventions, postoperative treatment with somatostatin may has a benefit regarding POPF. Further investigation is needed for the shown reduction of POPF after glucocorticoid treatment. The often-prescribed perioperative medical treatments using PERT, Erythromycin, Insulin and PPIs lack high quality evidence in comparative studies.

**The impact of pre-treatment alpha 1-fetoprotein and carbohydrate antigen (CA) 19-9 levels on the long-term prognosis of hepatocellular carcinomas and intrahepatic cholangiocarcinomas.**
ID: 425

**Kategorie: DGAV - Hepato-pankreato-biliäre Chirurgie**

Astrid Bauschke[1], Annelore Altendorf-Hofmann[1], Aladdin Ali-Deeb[1], Utz Settmacher[1]

[1]*Universität Jena, Klinik für Allgemein-, Viszeral- und Gefäßchirurgie, Jens, Deutschland*

**Background:**
The most important factors for long term survival after diagnosis of a malignant solid liver tumor are tumor type, tumor UICC-stage and type of treatment. Alpha 1-fetoprotein (AFP) and carbohydrate antigen (CA) 19-9 are established diagnostic markers for hepatocellular carcinomas (HCC) and intrahepatic cholangiocarcinomas (ICC), but their role in the long-term prognosis is still unclear.

**Materials and methods:**
We reviewed 1607 consecutive patients with solid liver tumors introduced to our department from 2000 to 2019. Pathological findings were HCC (8170/3) in 818 cases and iCC (8160/3) in 341 cases. 448 tumors had no histological confirmation of the tumor type. They were classified clinically as HCC or CCC. All patients were followed until death or at least for 10 years after diagnosis. Treatment decisions for all patients were made in an interdisciplinary conference.
To analyze the influence of therapy, tumor stage, coexisting liver cirrhosis and tumor markers on long-term prognosis we performed multivariate analyses (COX) for all patients and for the subgroups defined by the different types of therapy. Tumours with pre-treatment AFP-levels within the normal range of our laboratory were called AFP-negative. All analyses were performed with SPSS version 26 software.

**Results:**
542 patients (33,8%) were treated in curative intent (228 liver transplantations, 314 partial hepatectomies), 395 patients had palliative local therapies (TACE. SIRT, microwave ablation, percutaneous radiation) and 670 patients had systemic therapies or best supportive care. 40.3% of patients had no cirrhosis, 21.1%, 17.6% and 9.2% had Child A, B and C cirrhosis respectively. 303 patients (18.9%) had lymph node or distant metastases at time of diagnosis (UICC-stage IV).
32.9 % of HCCs were AFP-negative. 24.4% of iCCs had pre-treatment Ca 19-9-levels <20 U/l.
5-year survival rates of patients with HCC were pairwise statistically significant different depending on type of therapy. In patients with iCC there was no statistically significant difference in 5-year survival rates between patient treated in curative intent and patients with palliative local therapies. For patients with HCC as well as iCC the 5-year survival rates of patients without cirrhosis or with Child A cirrhosis were statistically significant better than the 5-year survival rates for patients with Child B/C cirrhosis. The same was true for patients with stage I-III versus stage IV.
The univariate analysis of all patients with HCC the pre-treatment AFP showed a statistically significant influence on the observed 5- and 10-year survival for all cut-off levels (7, 20, 35, 100, 400, 1000 ng/ml). If patients were stratified by therapy the pre-treatment AFP showed a statistically significant influence on the observed 5- and 10-year survival for all cut-off levels for patients with palliative local therapies and patients with systemic therapies or best supportive care. For patients treated with curative intent only a cut-off level of 20 ng/ml showed statistically significant influence on the observed 5- and 10-year survival.
In univariate analysis of all patients with iCC the pre-treatment CA 19-9 showed a statistically significant influence on the observed 5- and 10-year survival for the cut-off levels 20 U/ml and 1000 U/ml.

Multivariate analysis for tumors only clinically classified as HCC proved type of therapy, Child stage, level of preoperative AFP and stage to be independent statistically significant factors for 5-year survival.
Multivariate analysis for all histologically proven HCCs type of therapy, level of preoperative AFP and UICC-stage were independent statistically significant factors for 5-year survival, but Child stage was not.
In multivariate analysis for all histologically proven HCCs treated with curative intent therapy (transplantation versus partial hepatectomy) only UICC-stage had an independent statistically significant influence on 5-year survival, Child stage, type of therapy and level of preoperative AFP had not. In contrast to that the factors therapy (local therapy versus systemic therapies or best supportive care), Child stage, level of preoperative AFP and UICC-stage showed an independent statistically significant influence on 5-year survival for HCCs treated with palliative intent.
Multivariate analysis for all histologically proven iCCs showed type of therapy, level of preoperative CA 19-9 and UICC-stage but not Child stage to be independent statistically significant factors for 5-year survival.
Multivariate analysis for all histologically proven iCCs treated with curative intent, only UICC-stage was an independent statistically significant factor for 5-year survival, type of therapy, level of preoperative CA 19-9 and Child stage were not.
In contrast to that in multivariate analysis for all histologically proven iCCs treated with palliative intent, type of therapy and level of preoperative CA 19-9 were independent statistically significant factors for 5-year survival, UICC-stage and Child stage were not.

**Conclusion:**
Pre-treatment AFP levels show a statistically significant influence on observed 5- and 10-year survival in univariate analysis. In multivariate analyses the AFP-level is an independent statistically significant factor for survival only in patients treated with palliative intent. The same holds true for histologically proven iCCs and CA 19-9.
Therefore, pre-treatment levels of tumor markers together with Child stage and UICC-stage can help to estimate the long-term survival for palliative treated patients. For estimating the long-term survival for patients treated with curative intent the pre-treatment levels of tumor markers are of limited value.

**Total Pancreatectomy with Islet Autotransplantation for Chronic Pancreatitis: First Data from the Robotic Approach**
ID: 438

**Kategorie: DGAV - Hepato-pankreato-biliäre Chirurgie**

Benedict Kinny-Köster[1,2], Christi M. Walsh[1], Zhaoli Sun[1], Mahya Faghih[3], Niraj M. Desai[1], Daniel S. Warren[1], Rita R. Kalyani[4], Vikesh K. Singh[3], John L. Cameron[1], Christopher L. Wolfgang[2], Martin A. Makary[1], Jin He[1]

[1]*Johns Hopkins University School of Medicine, Department of Surgery, Baltimore, United States*, [2]*New York University Grossman School of Medicine, Department of Surgery, New York, United States*, [3]*Johns Hopkins University School of Medicine, Department of Medicine, Division of Gastroenterology and Hepatology, Baltimore, United States*, [4]*Johns Hopkins University School of Medicine, Department of Medicine, Division of Endocrinology, Diabetes, and Metabolism, Baltimore, United States*

**Background:**
Total pancreatectomy with islet autotransplantation (TPIAT) treats refractory pain in chronic pancreatitis and mitigates postoperative brittle diabetes. The procedure is gaining traction in the United States due to accumulating evidence of favorable long-term endocrine function but is mostly performed through laparotomy. Having pioneered a minimally-invasive (MIS) TPIAT program, we moved from a laparoscopic to the robotic approach and present patient outcomes compared to open TPIAT.

**Materials and methods:**
Between 2013 and 2021, 61 adult patients underwent TPIAT after a comprehensive evaluation (64% hereditary chronic pancreatitis). Pancreatic islets were isolated on-site during the procedure. We retrospectively analyzed intraoperative surgical and islet cell characteristics, postoperative morbidity and mortality, and 1-year glycemic outcomes.

**Results:**
MIS TPIAT was performed in 41 patients (67%, 15 robotic and 26 laparoscopic procedures), and 20 patients (33%) had an open operation. MIS TPIAT demonstrated similar results compared to open TPIAT for operative time (mean 577 vs 562 minutes, p=0.54), length of hospital stay (mean 10.4 vs 12.6 days, p=0.10), 30-day readmission rate (27 vs. 15%, p=0.30), and 90-day complication rate (54 vs. 65%, p=0.40). Random c-peptide levels were detectable postoperatively in 93 vs 90% (p=0.72) and at 1-year in 82 vs 70% (p=0.33) of patients. MIS TPIAT was associated with decreased intraoperative blood loss (mean 481 vs. 843 mL, p=0.03) and shorter length of intensive care unit stay (mean 2.9 vs. 4.5 days, p=0.002). The mean length of hospital stay after robotic TPIAT was 8.6 days, shorter than for laparoscopic (11.5 days, p=0.03) and open TPIAT (mean 12.6 days, p=0.02). Both MIS approaches had a 1-year mortality rate of 0%.

**Conclusion:**
Robotic TPIAT was associated with a decreased length of hospital stay, indicating enhanced recovery. More importantly, we also demonstrate that MIS TPIAT was similar on measures of safety, pain control, and 1-year glycemic efficacy compared to laparotomy. Our evolved experience verifies that IAT is valuable and suggests that the robotic approach could become the standard of care for TPIAT.

**APRI+ALBI is associated with adverse outcome after liver resection and shows higher potential than icg-clearance in the prediction of posthepatectomy liver failure - an international multi center study**
ID: 442

**Kategorie: DGAV - Hepato-pankreato-biliäre Chirurgie**

Jonas Santol[1,2], Sarang Kim[1], Lindsey Gregory[3], Hubert Hackl[4], Ruth Baumgartner[5], David Pereyra[6], Anastasia Murtha-Lemekhova[7], Emrullah Birgin[8], Severin Gloor[9], Eva Braunwarth[10], Markus Ammann[11], Daphni Ammon[6], Marijana Ninkovic[10], Anna Kern[6], Felix Huber[6], Yawen Dong[1], Cornelius Thiels[3], Susanne Warner[3], Rory Smoot[3], Mark Truty[3], Michael Kendrick[3], David Nagorney[3], Sean Cleary[3], Guido Beldi[9], Nuh Rahbari[8], Katrin Hoffmann[7], Stefan Gilg[12], Alice Assinger[2], Thomas Grünberger[1], Patrick Starlinger[3]

[1]*Viennese Health Network, HPB Center, Clinic Favoriten, Department of Surgery, Vienna, Österreich*, [2]*Center of Physiology and Pharmacology, Medical University of Vienna, Department of Vascular Biology and Thrombosis Research, Vienna, Österreich*, [3]*Mayo Clinic, Rochester, Division of Hepatobiliary and Pancreatic Surgery, Department of Surgery,, Rochester, United States*, [4]*Biocenter, Medical University of Innsbruck, Institute of Bioinformatics, Innsbruck, Österreich*, [5]*Karolinska University Hospital, Division of Oncology, Stockholm, Sweden*, [6]*Medical University of Vienna, Department of General Surgery, Division of Visceral Surgery, , Vienna, Österreich*, [7]*Heidelberg University Hospital, Department of General, Visceral, and Transplantation Surgery, Heidelberg, Deutschland*, [8]*University Medicine Mannheim, Medical Faculty Mannheim, Heidelberg University, Department of Surgery, Mannheim, Deutschland*, [9]*Inselspital, Bern University Hospital, University of Bern, Department of Visceral Surgery and Medicine, Bern, Schweiz*, [10]*Medical University of Innsbruck, Department of Visceral, Transplantation and Thoracic Surgery, Innsbruck, Österreich*, [11]*State Hospital Wiener Neustadt, Department of Surgery, Vienna, Österreich*, [12]*Karolinska Institutet, Karolinska University Hospital, Division of Surgery, Department of Clinical Science, Intervention and Technology, Stockholm, Sweden*

**Background:**
Over the recent years preoperative risk stratification for patients undergoing liver resection has greatly improved. The development or preoperative dynamic liver function tests like indocyanine green (icg) clearance testing has been a major innovation in this regard. Even though improvements have been made, still about 10% of patients develop posthepatectomy liver failure (PHLF), depending on the underlying liver disease. An alternative, which has been evaluated for preoperative risk assessment, have been preoperative scores or grades based on routine laboratory parameters. Two examples being the aspartate to platelet ratio (APRI) and the Albumin-Bilirubin grade (ALBI). Both have been associated with the development of PHLF. The summative combination of APRI and ALBI APRI+ALBI has shown even improved predictive potential compared to both tests alone. Through the combination of both scores APRI+ALBI seems to reflect both the degree of chronic liver disease as well as liver injury caused by neoadjuvant chemotherapy. A comparison of both APRI+ALBI and icg clearance however has not been made, as of yet. The aim of this study was to compare both APRI+ALBI and icg clearance in their predictive potential for PHLF and validate these findings.

**Materials and methods:**
We included 622 patients out of a prospectively managed biobank, as an exploratory cohort. All patients underwent liver surgery either at the General Hospital Vienna or at the clinic Landstrasse. Also, in all 622 patients icg clearance was preoperatively measured. Further 1906 patients from 8 different international institutions were retrospectively included as a validation cohort. Participating institutions were Karolinska Institutet (Stockholm, Sweden), Mayo Clinic Rochester (Minnesota; USA), University Hospital Heidelberg (Heidelberg, Germany), University Hospital Mannheim (Mannheim,

Germany), Inselspital University Hospital Bern (Bern, Switzerland), University Hospital Innsbruck (Innsbruck, Austria), Clinic Landstraße (Vienna, Austria), State Hospital Wiener Neustadt (Wiener Neustadt, Austria). APRI+ALBI scores were calculated for the exploratory and the validation cohort, from parameters measured in routine laboratory tests. Postoperative patient outcome was retrospectively assessed. PHLF was graded by the criteria put forth by the international study group on liver surgery (ISGLS) and was defined as PHLF grade B and C. Icg clearance was measured in plasma disappearance rate (PDR) and retention after 15 minutes (R15).

**Results:**
We first analyzed differences in our parameters depending on the development of PHLF. APRI+ALBI, and icg clearance showed significant difference when comparing patients with and without PHLF (APRI+ALBI $p < 0.001$, R15 $p = 0.002$, PDR $p = 0.005$). Interestingly only APRI+ALBI showed significant differences in in patients who suffered from 90 days mortality (APRI+ALBI $p = 0.001$). We then analyzed the predictive potential of APRI+ALBI and icg clearance for the development of PHLF grade B + C using receiver operating characteristic (ROC) curve analysis. APRI+ALBI outperformed icg clearance when comparing their respective areas under the curve (AUC) (APRI+ALBI AUC = 0.785, $p < 0.001$; R15 AUC = 0.643, $p = 0.014$; PPDR AUC = 0.643, $p = 0.014$). When only evaluating discriminatory power for the development of PHLF grade C, APRI+ALBI improved its predictive potential (APRI+ALBI AUC = 0.828, $p < 0.001$; R15 AUC = 0.590, $p = 0.581$; PDR AUC = 0.581, $p = 0.379$). We then defined two cut-offs. One cut-off of ≥ -2.43 (negative predictive value (NPV) = 98%, positive predictive value (PPV) = 14%, $p < 0.001$) with a high NPV, to identify patients who could safely be resected, and one cut-off of ≥ -0.86 (NPV = 95%, PPV = 56%, $p < 0.001$) with a high PPV, to identify high risk patients. As patients undergoing major liver resection are at especially high risk for development of PHLF a third cut-off of -0.98 was defined (NPV = 93%, PPV = 57% $p < 0.001$). We then validated our cut-offs in our validation cohort. Comparable to the exploratory cohort the high NPV cutoff could accurately predict which patients had low risk for adverse outcome after resection (NPV = 93%, PPV = 18%, $p < 0.001$). The high risk coutoff could also be validated. Almost 30% of patients above the cutoff suffered from PHLF B + C and only 1 % of patients without PHLF B + C had APRI+ALBI scores higher -0.86 (NPV = 88%, PPV = 29%, $P < 0.001$). When evaluating the high risk cutoff for major resections we could also show that patients with APRI+ALBI scores above -0.98 were more likely to suffer from PHLF B + C (NPV = 86%, PPV = 33%, $p < 0.001$).

**Conclusion:**
This study shows that APRI+ALBI outperforms icg-clearance as a predictor for PHLF B + C. It could be that, as icg clearance only reflects the excretory functions of the liver, APRI+ALBI potentially more accurately reflects the livers physiologic responsibilities as a whole. Especially for high grade PHLF like PHLF B + C and C alone APRI+ALBI showed high discriminatory power, with these grades of PHLF being more clinically relevant, in comparison to grade A for example. We could also validate our findings in an international validation cohort. It is especially remarkable, that even when comparing results from 8 different international institutions, we can see a similar potential of APRI+ALBI as a predictor for postoperative outcome after liver resection. As APRI+ALBI can be easily calculated, using only routine laboratory parameters and a smartphone application, more frequent implementation of APRI+ALBI into the clinical routine, could potentially benefit patient outcome in a significant way.

**Histomorphological features of the pancreatic resection margin – a score to predict the form of a postoperative pancreatic fistula?**
ID: 458

**Kategorie: DGAV - Hepato-pankreato-biliäre Chirurgie**

Tim Fahlbusch[1], Waldemar Uhl[1], Orlin Belyaev[1]

[1]*Ruhr-Universität Bochum, St. Josef-Hospital, Klinik für Allgemein- und Viszeralchirurgie, Bochum, Deutschland*

**Background:**
Over the last decades, mortality rates in pancreatic surgery could be reduced. Still, the postoperative pancreatic fistula (POPF) ranks as one of the most frequent complications occasionally leading to life threatening haemorrhages (PPH). The prediction of the occurrence and form of the POPF could further decrease the risk after pancreatic resections. We developed a scoring system based on histomorphological criteria to predict the appearance of POPF earlier on. In this study the scoring system differentiates between patients developing high risk and low risk fistulas.

**Materials and Methods:**
We enrolled 92 patients in the study who underwent a pancreatic head resection. Data about demographics, co-morbidities and outcome were gathered. Frozen sections of the pancreatic resection margin were analysed and data like pancreatic duct size, fibrosis and fat content were transformed into a scoring system.

**Results:**
48 patients developed a grade B and 44 patients a grade C fistula. Groups were similar regarding age, sex, comorbidities and BMI. The grade C group was characterized by statistically significant higher mortality rates (0 vs 22, $p<0.001$), longer stays in ICU (2.6 vs 21.1, $p<0.001$) and in hospital (29.8 vs 44.6, $p<0.001$). PPHs were more often reported in grade C group (1 vs 25, $p<0.001$). The scoring system amounted to medians of $8.0\pm1.9$ in the grade B group and $7.0\pm2.1$ ($p=0.736$) in grade C group, predicting a higher risk for complications in grade C group.

**Conclusion:**
In pancreatic surgery various complications are reported, but – among others - POPF are highly frequent. Our study group presents a scoring system based on pathological results, predicting the quality of POPF. In combination with additional data, the histological score could help prevent patients in pancreatic surgery from life threating events.

**Prognostic impact of resection margin status in patients with pancreatic cancer undergoing neoadjuvant treatment – meta-analysis of aggregate patient data**
ID: 540

**Kategorie: DGAV - Hepato-pankreato-biliäre Chirurgie**

Carl-Stephan Leonhardt[1], Thomas Hank[1], Leonie Frank-Moldzio[1], Oliver Strobel[1], Ulla Klaiber[1]

[1]*Medical University of Vienna, Department of General Surgery, Division of Visceral Surgery, Vienna, Österreich*

**Background:**
Neoadjuvant treatment is increasingly utilized in patients with pancreatic ductal adenocarcinoma (PDAC). While neoadjuvant treatment is the only chance for successful conversion surgery in patients with locally advanced tumors, the main rationale for neoadjuvant treatment in borderline resectable PDAC is to increase the chance for microscopic tumor clearance. The resection margin (R) status has been identified as an independent prognostic factor of survival in patients undergoing upfront resection for PDAC. The prognostic significance of the R status in patients with PDAC undergoing resection following neoadjuvant treatment is poorly investigated. The aim of this cohort study and meta-analysis was to investigate the prognostic significance of R status in PDAC patients treated with neoadjuvant therapy.

**Materials and methods:**
Cohort studies investigating associations between resection margins and survival in PDAC patients undergoing resection following neoadjuvant treatment were searched in Medline, Web of Science, and the Cochrane Central Register of Controlled Trials (CENTRAL). The search was limited to studies published between 2010 and 2022. Hazard ratios (HR) were pooled using a random-effects model meta-analysis and the results presented with the corresponding 95% confidence interval (CI). Subgroup analyses were performed to account for inter-study heterogeneity regarding the definition of margin status, and inter-study differences in methodological quality. R status was categorized as R0 ≥1 mm, R1 <1 mm, and R1 direct (microscopic tumor infiltration at margin). Margin categories were compared between these groups and tested for survival prediction. Results from proportional hazards regression (Cox model) analysis were included in meta-analysis.

**Results:**
Of 2,347 abstracts screened, 21 studies with 4,941 patients were included in the final qualitative and quantitative synthesis. R1 direct rate was 8%, R0 rate 32%, R0 ≤1mm rate 11%, and R0 ≥1mm rate 48%, respectively. R0 resection was associated with prolonged overall survival compared to combined R1 direct resections (HR 1.38, 95% CI 1.01 to 1.90, p=0.04) using univariable data. No independent associations between different definitions of the R status and survival could be observed. Subgroup analyses including only data from high quality studies did not substantially alter the results.

**Conclusion:**
The results from this meta-analysis show that R status is a significant prognostic factor of overall survival in PDAC patients undergoing resection after neoadjuvant treatment. Pooled data from multivariable analysis, however, did not confirm R status as an independent prognostic factor. Difficulties in pathological assessment of specimen after neoadjuvant treatment combined with the limited number of studies and non-standardized pathology protocols hamper inter-study comparability. Uniform and internationally accepted definitions of margin status are urgently needed.

### Baseline Interleukin-6 serves as a preoperative marker for liver fibrosis

ID: 561

**Kategorie: DGAV - Hepato-pankreato-biliäre Chirurgie**

Dominik Koch[1], Dionysios Koliogiannis[1], Matthias Ilmer[1], Joachim Andrassy[1], Markus Guba[1], Jens Werner[1], Florian Kühn[1]

[1]*Klinikum der LMU München, Klinik für Allgemein-, Viszeral- und Transplantationschirurgie, München, Deutschland*

**Background:**
Liver fibrosis (LF) is characterized by impaired function and regenerative capacity of the liver and can lead to significantly increased morbidity and mortality in the context of surgical liver resection (LR). This is why the knowledge of the degree of LF preoperatively would be desirable. Interleukin-6 (IL-6) is known to play a key role in the pathogenesis of LF, but its exact value as a preoperative marker is unknown. The study aimed to investigate the correlation between preoperatively determined IL-6 and the presence of LF.

**Materials and methods:**
In this prospective study, we determined IL-6 in 108 patients who were scheduled for LR. Postoperatively, the removed liver tissue was classified by a blinded pathologist with regard to the degree of LF according to the Desmet classification (0 to 4). Baseline IL-6 and LF were correlated with each other.

**Results:**
In total, 108 patients were prospectively included prior to LR. 31 patients had liver cirrhosis (Desmet 4) and another 27 patients showed preoperatively elevated infection parameters (CRP, leukocytes). These patients were excluded. The remaining 27 patients had LF grade 0 or 1 according to Desmet in 18 cases and LF grade 2 or 3 (distinctive fibrosis) in 9 cases. Correlation of LF with preoperatively determined IL-6 yielded significantly higher IL-6 levels in the group of patients with distinctive fibrosis (Desmet 2 or 3) compared to the group classified as Demet 0 or 1 ($p=0.0001$).

**Conclusion:**
In the context of LR, our results showed a correlation of preoperatively determined IL-6 with the extent of LF present. High serum baseline IL-6 concentrations were associated with a higher degree of LF. Our results suggest that baseline IL-6 might serve as a valuable parameter to assess LF prior to LR.

### Prognostic scoring systems in recurrent colorectal liver metastases

ID: 573

**Kategorie: DGAV - Hepato-pankreato-biliäre Chirurgie**

Katharina Jöchle[1], Iakovos Amygdalos[1], Jan Bednarsch[1], Alexandros Chrysos[1], Franziska Meister[1], Zoltan Czigany[2], Daniel Heise[1], Tom Florian Ulmer[1], Ulf Neumann[1], Sven Arke Lang[1]

[1]*RWTH Aachen, Klinik für Allgemein-, Viszeral- und Transplantationschirurgie, Aachen, Deutschland*,
[2]*Charité - Universitätsmedizin Berlin, Chirurgische Klinik, Berlin, Deutschland*

**Background:**
Prognostic scoring systems were developed to identify patients with colorectal liver metastases (CRLM) who are more likely to benefit from surgery by stratifying them in different risk categories. However, data on the utility of these scoring systems in patients with recurrent CRLM (recCRLM) are very limited. Therefore, this study aims to analyze the predictive value of prognostic scoring systems in patients with recCRLM.

**Materials and methods:**
Patients with recCRLM who underwent repeat curative intent hepatectomy at the RWTH University Hospital Aachen, Germany between 2010 and 2021 were included. Likelihood ratio (LR) $\chi^2$, linear trend (LT) $\chi^2$ and Akaike Information Criterion (AIC) were used to evaluate the predictive value of Fong's clinical risk score (CRS), Nordlinger score, Nagashima score, RAS mutation CRS, tumor burden score (TBS), GAME score, CERR score and Glasgow prognostic score on overall survival (OS).

**Results:**
167 patients underwent repeat hepatectomy for recCRLM with a median OS of 27 months (3-131) and a 5-year OS rate of 36%. Four of the examined eight predictive scores (the Nordlinger risk score, the Nagashima score, the GAME score and the CERR score) showed a significant discriminatory ability with the CERR score having the best prognostic performance (LT $\chi^2$ 16.86, LR $\chi^2$ 15.15, AIC 175.26).

**Conclusion:**
While the CERR score had the best prognostic ability to predict OS for recCRLM, not all of the examined scoring systems showed a significant discriminatory ability and are therefore of limited utility in recurrent disease.

**Association of pancreatic PArenchymal RISk factors with postoperative pancreatic fistula after partial pancreaticoduodenectomy - a prospective trial (PARIS trial)**
ID: 577

**Kategorie: DGAV - Hepato-pankreato-biliäre Chirurgie**

Fabian Schuh[1], Berk Yildirim[1], Rosa Klotz[1], Frank Pianka[1], Matthias Fink[2], Manuel Feisst[3], Christoph Eckert[4], Constantin Schwab[4], Colette Doerr-Harim[1], André Mihaljevic[1], Markus Büchler[1], Pascal Probst[1]

[1]*Department of General, Visceral and Transplantation Surgery, University of Heidelberg, Heidelberg, Germany, Heidelberg, Deutschland*, [2]*Department for Diagnostic and Interventional Radiology, University of Heidelberg, Heidelberg, Germany, Heidelberg, Deutschland*, [3]*Institute of Medical Biometry and Informatics, University of Heidelberg, Germany, Heidelberg, Deutschland*, [4]*Institute of Pathology, University of Heidelberg, Heidelberg, Germany, Heidelberg, Deutschland*

**Background:**
Partial Pancreatoduodenectomy (PD) is the treatment of choice for numerous malignant and benign diseases of the pancreas. Although postoperative mortality after PD has decreased below 5%, morbidity remains high even in specialized pancreatic cancer centres with postoperative complication rates of up to 40%. Postoperative complications have been uniformly defined by the International Study Group for Pancreatic Surgery (ISGPS) over the last decades which allows standardized reporting of postoperative pancreatic fistula (POPF).

POPF is one of the most common PD- associated complications occurring in 15-30%. POPF-related haemorrhage represents one of the most severe complications after PD. Multiple risk factors have been identified that are associated with the development of POPF following PD including patient associated risk factors (e.g. BMI), perioperative risk factors (e.g. volume management, preoperative total bilirubin), surgeon-associated risk factors (e.g. experience in PD surgery) and pancreas-associated risk factors (e.g. histology, diameter of the main pancreatic duct (MPD), texture and the location of the pancreatic duct). Several fistula risk scores already exist, whereas a prospective study assessing the diagnostic value of a combination of pancreas characteristics is lacking.

Aim of the monocentric, prospective and diagnostic PARIS trial is to investigate the impact of pancreas characteristics (location and diameter of the MPD and parenchymal texture) on the risk of developing a POPF in patients undergoing PD.

**Materials and methods:**
All patients scheduled for elective PD for any indication at the Department of General, Visceral and Transplantation Surgery of the University of Heidelberg were screened for eligibility.
As diagnostic factors, the diameter of the MPD and texture of the pancreatic gland (by haptic measurement of the surgeon as well as usage of a durometer) as well as radiological and histopathological features were recorded. Furthermore, the new four classed risk classification system by the ISGPS established in 2021 was recorded. The postoperative course was monitored prospectively within a follow-up period of 30 days.
The primary endpoint was the occurrence of POPF within 30 days according to the ISGPS definition. Secondary endpoints included delayed gastric emptying, postpancreatectomy heamorrhage, chyle leakage (according to ISGPS definitions), bile leakage (defined by the recent ISGLS classification) and postoperative morbidity and mortality (according to the Clavien-Dindo-Classification).
All data was gathered in an electronic case report form (eCRF) and successively implemented in the REDCap system (Vanderbilt University, Nashville, United States). The diagnostic value of the above-mentioned pancreatic risk factors for POPF were evaluated in a univariate and multivariate analysis, with the main focus on the correlation between MPD size, pancreas texture and the development of POPF.

**Results:**
The analysis of the gathered data is not completed at the time of the submission of the abstract. In total 498 patients were screened for eligibility of which 227 patients were not eligible due to operations other than PD, inoperability or other reasons. In total, 200 patients are included in the final analysis, with expected results in November 2022.

**Conclusion:**
Pancreas-associated risk factors are clinically important as they would offer the opportunity for an easy-to-use risk classification, that might guide intraoperative and postoperative decision-making process including placement of drains, degree of resection, intensity of follow-up, need for intensive care observation, administration of somatostatin analogues and others. Therefore, the presented study will deliver important data for a pancreas-derived individually adapted approach in surgical and postoperative treatment. Furthermore, this trial will deliver data to evaluate the recently published four grouped ISGPS classification system of pre-/ and intraoperatively measured pancreatic gland characteristics for the assessment of the risk of a clinically relevant POPF.

**PVE/HVE improves survival compared to PVE alone – A survival analysis of the DRAGON 0 cohort**
ID: 675

**Kategorie: DGAV - Hepato-pankreato-biliäre Chirurgie**

Jan Heil[1], Remon Korenblik[2,3], Wolf Otto Bechstein[1], Bram Olij[2,3,4], Andreas Schnitzbauer[1], Jens Smits[2,3], Ronald van Dam[2,3,4], Erik Schadde[5,6,7]

[1]*Goethe-Universität Frankfurt, Klinik für Allgemein-, Viszeral-, Transplantations- und Thoraxchirurgie, Frankfurt, Deutschland*, [2]*Department of Surgery, Maastricht University Medical Centre, Maastricht, the Netherlands, Maastricht, Netherlands*, [3]*GROW – School for Oncology and reproduction, Maastricht University, Maastricht, the Netherlands, Maastricht, Netherlands*, [4]*Department of General, Visceral and Transplant Surgery, Uniklinik RWTH Aachen, Aachen, Germany, Aachen, Deutschland*, [5]*Chirurgisches Zentrum Zürich (CZZ), Klinik Hirslanden Zurich, Zurich, Switzerland, Zürich, Schweiz*, [6]*hirurgie Zentrum Zentralschweiz (CZZ), Hirslanden St. Anna, Lucerne, Switzerland, Zürich, Schweiz*, [7]*Department of Surgery, Rush University Medical Center Chicago, Chicago, IL, United States, Zürich, Schweiz*

**Background:**
Regenerative liver surgery improves resectability in patients with a small future liver remnant (FLR) at increased risk of post-hepatectomy liver failure (PHLF). Portal vein embolization (PVE) is the gold standard of regenerative liver surgery. However, novel methods to induce more rapid hypertrophy than PVE result in accelerated liver growth and higher resection rates compared to PVE alone. The purpose of this study is to evaluate the mid-term oncological outcome after simultaneous of portal and hepatic vein embolization (PVE/HVE) compared with PVE alone following the cohorts of the previously published DRAGON 0 study.

**Materials and methods:**
In this retrospective cohort study, all centers of the international DRAGON collaborative provided data of patients undergoing PVE/HVE and PVE between 2016 and 2019. Primary and secondary liver tumors were included. Kaplan-Meier analysis was performed to last follow-up to analyze mid-term recurrence-free and overall survival. Factors impacting survival were analyzed by multivariable analysis using logistic regression.

**Results:**
In total, 199 patients from 7 centers of the DRAGON collaborative were included. PVE/HVE was performed in 39 and PVE alone in 160. Following PVE/HVE more patients were resected compared to PVE alone (PVE/HVE: 90% *vs.* PVE: 68%, p=0.007). After a median time to follow-up of 32 (PVE/HVE) and 9 months (PVE) (p=0.13), there was a significant difference in overall survival in favor of PVE/HVE (PVE/HVE: 58 months *vs.* PVE: 19 months, p=0.009), while there was no difference in recurrence/progression-free survival (PVE/HVE: 27 months *vs.* PVE: 14 months, p=0.47). Multivariable analysis revealed PVE/HVE to be an independent predictor of survival (odds ratio: 4.24 (confidence interval: 1.17 – 15.42), p=0.024).

**Conclusion:**
In patients with the need for regenerative liver surgery, rapid liver regeneration by PVE/HVE appears to improve survival compared to PVE alone. Prospective randomized trials of separate tumor entities are warranted to further investigate, if PVE/HVE improves survival over PVE alone.

**Middle segment-preserving pancreatectomy to avoid pancreatic insufficiency: individual patient data analysis of worldwide published cases from 2003-2021**
ID: 724

**Kategorie: DGAV - Hepato-pankreato-biliäre Chirurgie**

Thomas Pausch[1], Josefine Dincher[1], Ulrike Heger[1], Pietro Contin[1], Pascal Probst[2,3], Rosa Klotz[1,3], Thilo Hackert[1]

[1]*Allgemein-, Viszeral- und Transplantationschirurgie, Universitätsklinikum Heidelberg, Heidelberg, Deutschland*, [2]*Kantonspital Thurgau, Chirurgische Klinik, Frauenfeld, Schweiz*, [3]*Studienzentrum der Deutschen Gesellschaft für Chirurgie (SDGC), Heidelberg, Deutschland*

**Background:**
Middle segment-preserving pancreatectomy (MPP) can treat multilocular diseases in the pancreatic head and tail, while avoiding impairments caused by total pancreatectomy (TP). This study collected all published cases of MPP and analyzed outcomes from their individual patient data (IPD) in comparison with TP patients.

**Materials and methods:**
We conducted a systematic literature review for MPP published from 2003–2021 and collected IPD from original authors to generate a sample of independent cases. We included a comparator group of patients with benign pathologies undergoing TP from our hospital. MPP patients were summarized and analyzed in terms of i) clinical baseline characteristics, ii) intraoperative course, iii) postoperative outcome and iv) survival. MPP patients were compared to TP patients for i-iii. Post-operative outcomes for MPP were tested for prognostic indicators using generalized linear models in a multimodel framework.

**Results:**
The review generated IPD for 28 MPP cases from 17 articles, to which we added 1 MPP case and 14 TP cases from our own patients. MPPs took longer than TPs, but MPP had substantial benefits over TP in preserving pancreatic functionality. New-onset diabetes and exocrine pancreatic insufficiency occurred in 29% and 42% of MPP patients, respectively, compared to ubiquitous prevalence among TP patients. Nevertheless, POPF Grade B occurred in 54% of MPP patients, a complication avoidable with TP. Longer pancreatic remnants were a positive prognostic indicator for shorter and less eventful hospital stays with fewer complications. Complications from endocrine and exocrine pancreatic insufficiency were more closely associated with older patients. Long-term survival prospects after MPP appeared strong (median up to 110 months), but survival was lowered in cases with recurring malignancies and metastases (median <40 months).

**Conclusion:**
This study demonstrates MPP is a feasible treatment alternative to TP for selected cases of multilocular diseases in the pancreatic head and tail, especially in younger and healthier patients. It can avoid pancreoprivic impairments, but at the risk of perioperative morbidity.

**Inadequate lymph node examination leads to misclassification and affects survival in resected pancreatic ductal adenocarcinoma**
ID: 756

**Kategorie: DGAV - Hepato-pankreato-biliäre Chirurgie**

Rüdiger Göß[1,2,3], Carsten Jäger[1,2,3], Julie Perinel[4], Ilaria Pergolini[1,2,3], Elke Demir[1,2,3], Okan Safak[1,2,3], Florian Scheufele[1,2,3], Stephan Schorn[1,2,3], Alexander Muckenhuber[5], Mustapha Adham[4], Alexander Novotny[1], Güralp Ceyhan[6], Helmut Friess[1,2,3], Ekin Demir[1,2,3,6,7]

[1]*Klinikum rechts der Isar, Technical University Munich, School of Medicine, Department of Surgery, Germany, Deutschland*, [2]*German Cancer Consortium (DKTK), Partner Site Munich, Munich, Deutschland*, [3]*CRC 1321 Modelling and Targeting Pancreatic Cancer, Munich, Deutschland*, [4]*E. Herriot Hospital, Hospices civils de Lyon, Department of Digestive Surgery, Lyon, France*, [5]*Klinikum rechts der Isar, Technical University Munich, School of Medicine, Institute of Pathology, München, Deutschland*, [6]*Acibadem Mehmet Ali Aydinlar University, School of Medicine, Department of General Surgery, HPB-Unit, Istanbul, Turkey*, [7]*Else Kröner Clinician Scientist Professor for Translational Pancreatic Surgery, Munich, Deutschland*

**Background:**
Lymph node status is considered one of the strongest prognostic factors for survival in resected pancreatic ductal adenocarcinoma (PDAC). However, the minimum number of examined lymph nodes (ELN) required for adequate staging has not been established in PDAC. The objective of the study was to investigate the influence of ELN on staging and survival in different types of resection for PDAC.

**Materials and methods:**
466 patients undergoing partial or total pancreatectomy for PDAC at two European university hospitals between 2007 and 2016 were retrospectively reviewed. Clinicopathological data were analyzed to identify the minimum number of ELN that influence prognosis and lymph node status. Survival analyses were performed to verify adequate staging.

**Results:**
341 (73%) patients showed lymph node metastasis (N1/N2), whereas 125 (27%) patients had no lymph node involvement (N0). To detect lymph node involvement with a probability of 95%, at least 20 lymph nodes (LN) were required for examination in pancreaticoduodenectomy, ≥25 LN in distal pancreatectomy, and ≥22 LN in total pancreatectomy. With increasing number of ELN, the proportion of positive LN increased. In node-negative patients, survival was significantly worse when < 15 LN were examined, with median overall survival of 32 months (<15 ELN) vs. 65 months (≥15 ELN) (p=0.013). These survival differences were also detectable in patients with N1 lymph node status, but not with N2 status. The examination of less than 15 lymph nodes was a significant negative predictor for overall survival in multivariate analysis (HR 1.725, CI 95% 1.317 - 2.260, p<0.001).

**Conclusion:**
The number of ELN in node-negative patients affects survival in PDAC. We identified possible misclassification of patients with N0 and N1, but not with N2 status when <15 LN were examined. Therefore, at least 15 LN must be examined for adequate staging. Standardized lymphadenectomy and thorough histopathological examination avoid false lymph node classification in PDAC.

**The impact of the time interval from diagnosis to curative-intent surgery on oncological outcome in patients with cholangiocarcinoma**
ID: 779

**Kategorie: DGAV - Hepato-pankreato-biliäre Chirurgie**

Anna Mantas[1], Dong Liu[1], Carlos Constantin Otto[1], Lara Rosaline Heij[1], Daniel Heise[1], Philipp Bruners[2], Sven Arke Lang[1], Tom Florian Ulmer[1], Ulf Peter Neumann[1], Jan Bednarsch[2]

[1]*Department of Surgery and Transplantation, University Hospital RWTH Aachen, Aachen, Deutschland*, [2]*Department of Diagnostic and Interventional Radiology, University Hospital RWTH Aachen, Aachen, Deutschland*

**Background:**
Cholangiocellular carcinoma (CCA) is the second most common primary malignant liver tumor. Being not only highly aggressive but also usually diagnosed at advanced stages, CCAs are associated with high morbidity and mortality rates. Surgical resection with curative intent is the gold standard for their treatment. Due to known limitations of the health care systems and unforeseeable events such as the covid pandemic, time from diagnosis to treatment (TTT) has gained great research interest in many malignancies. To this day, available data for CCA is limited. Thus, we aimed to investigate whether TTT is associated with oncological outcome in surgically treated CCC patients.

**Materials and methods:**
A monocentric cohort of 276 patients undergoing curative-intent liver resection for CCA between 2010 to 2021 in a large German hepatobiliary center were eligible for analysis. Patients undergoing neoadjuvant therapy were excluded from the analysis. Individuals were grouped according to TTT (≤30; 31-60; 61-90; >90 days) and compared regarding clinical characteristics by Kruskal-Wallis-analysis. Further, survival was compared using Kaplan-Meier analysis and characteristics associated with cancer-specific survival (CSS) and recurrence-free survival (RFS) of the study population investigated by univariate and multivariable Cox regressions.

**Results:**
The median CSS of the study cohort was 39 months (3-year-CSS=52 %, 5-year-CSS=42%) and the median RFS was 20 months (3-year-RFS=38%, 5-year-RFS=33%). In univariable Cox regressions, TTT was not associated with CSS (p=0.972) or RFS (p=0.854) of the study cohort, respectively. A grouped analysis with respect to TTT (≤ 30 days, n=106; 31-60 days, n=134; 61-90 days, n=44; >90 days, n=29 days) displayed a median CSS of 38 months, 33 months, 51 and 41 months and median RFS of 17 months, 22 months, 28 months and 20 months within these groups (p=0.965 log rank; p=0.738 log rank). Further, no statistical difference regarding oncological risk factors (tumor staging, pathological characteristics, etc.) were observed between the groups.

**Conclusion:**
To the best of our knowledge, this study is the first comprehensive analysis of TTT in surgically resected CCA patients. Within a large and representative European patient cohort, TTT was not associated with earlier tumor recurrence or reduced cancer-specific survival. A delay in treatment might therefore be justified in times of limited resources or to preoperatively improve the physical condition of the patient and conducted detailed preoperative staging examinations.

**Preoperative CT-based lung volumetry predicts postoperative respiratory complications in patients undergoing major curative liver resection for colorectal metastases.**
ID: 808

**Kategorie: DGAV - Hepato-pankreato-biliäre Chirurgie**

Suzan Elmaagacli[1], Christoph Thiele[2], Philipp Menne[1], Katharina Jöchle[1], Alexandros Chrysos[1], Lea Hitpass[3], Daniel Truhn[3], Philipp Bruners[3], Johannes Bickenbach[2], Gernot Marx[2], Tom Florian Ulmer[1], Ulf Neumann[1], Sven Arke Lang[1], Iakovos Amygdalos[1]

[1]*University Hospital RWTH Aachen, Department of General, Visceral and Transplantation Surgery, Aachen, Deutschland*, [2]*University Hospital RWTH Aachen, Department of Operative Intensive Care and Intermediate Care, Aachen, Deutschland*, [3]*University Hospital RWTH Aachen, Department of Diagnostic and Interventional Radiology, Aachen, Deutschland*

**Background:**
Colorectal liver metastases (CRLM) are the predominant factor limiting survival in patients with colorectal cancer. Patients requiring major resection for CRLM are at increased risk of perioperative morbidity. This study examines the predictive capability of body composition measurements carried out on preoperative computed tomographies (CT) of the thorax, in terms of postoperative complications.

**Materials and methods:**
Patients undergoing major curative liver resection for CRLM between 2010-2021 at the University Hospital RWTH Aachen, with a preoperative CT scan of the thorax within 6 weeks of surgery, were eligible for this retrospective study. Patients with recurrent metastases were excluded. Total lung volume (TLV) was calculated by volumetry software 3D-Slicer version 4.11.20210226 including Chest Imaging Platform extension. A receiver-operating curve (ROC) was used to define a cut-off value for TLV, to predict the occurrence of postoperative respiratory complications. These were defined as the presence of any of the following within 90 days of operation: respiratory failure, pulmonary embolism, pneumonia, pleural empyema, pleural effusion needing thoracentesis and pneumothorax needing thoracic drainage. Differences between patients with TLV below and above the cut-off were examined with chi square and Mann-Whitney U tests and logistic regression was used to determine independent risk factors for the development of respiratory complications.

**Results:**
A total of 124 patients were included, of which 35 (28%) developed respiratory complications. ROC analysis showed an Area Under the Curve (AUC) of 0.62 for prediction of respiratory complications ($p=0.03$), with a cut-off value for TLV of 4500mm$^3$ (Youden Index = 0.256). Patients with TLV<4500mm$^3$ suffered from significantly more respiratory complications (44% vs 21%, $p=0.006$). Logistic regression showed a decreased risk of respiratory complications for patients above the cut-off (odds ratio 0.329, $p=0.008$).

**Conclusion:**
Preoperative CT-based lung volume measurements can predict the development of respiratory complications after major liver resection for CRLM. These patients could benefit from aggressive pre-habilitation and postoperative physiotherapy. Further studies with a larger cohort and prospective design are necessary to explore the value and limitations of this technique.

**Textbook outcomes in patients undergoing surgical resection for clinical stage I-III pancreatic ductal adenocarcinoma – an analysis of the German national cancer registry (GCRG/ADT)**
ID: 843

**Kategorie: DGAV - Hepato-pankreato-biliäre Chirurgie**

Louisa Bolm[1], Natalie Petruch[1], Hryhoriy Lapshyn[1], Rüdiger Braun[1], Kim Honselmann[1], Richard Hummel[1], Monika Klinikhammer-Schalke[2], Kees Kleihues-van Tol[2], Sylke Zeissig[2], Tobias Keck[1], Ulrich Wellner[1]

[1]*Department of Surgery, University Medical Center Schleswig-Holstein, Campus Lübeck, 23562, Deutschland*, [2]*German Cancer Registry Group of the Society of German Tumor Centers - Network for care, quality and research in Oncology, Berlin (GCRG/ADT) , Berlin, Deutschland*

**Background:**
Pancreatic ductal adenocarcinoma (PDAC) therapy is a complex multidisciplinary treatment. Textbook outcome is a quality control endpoint of therapies concordant with the national treatment guidelines. We aim to evaluate textbook outcomes in patients with surgical resection for PDAC in Germany based on the national cancer registry (GCRG/ADT).

**Materials and methods:**
Patients with clinical stage I-III PDAC undergoing surgery alone and with perioperative therapy were identified from the GCRG/ADT database. Baseline characteristics, surgical and perioperative parameters and overall survival were evaluated.

**Results:**
1,392 patients from the GCRG/ADT database were included, median age was 69 years and 48.6% of the patients were female. Median time from diagnosis to surgical resection was 23 days. Regarding surgical outcomes, 1011 (72.6%) patients had a complete oncological resection (R0) while 341 (24.5%) had R1 resections and 40 (2.9%) had R2 resections. The median number of lymph nodes harvested was n=17, 180 (12.9%) patients had fewer than n=12 lymph nodes harvested. 883 (63.4%) patients underwent perioperative therapy with 95 (6.8%) having received neoadjuvant therapy alone, 691 (49.6%) with adjuvant therapy alone and 97 (7.0%) undergoing both together. The endpoint of textbook surgical outcome (R0 and number of LN harvested >= 12) was reached in 931 (66.9%) patients. The composite endpoint of textbook surgical and perioperative therapy outcome (R0, number of LN harvested >= 12, perioperative therapy received) was achieved in 761 (54.7%) patients. Median overall survival in patients with composite textbook outcome was 16.9 months, while those with non-textbook outcome had a median overall survival of 6.1 months (p < 0.001).

**Conclusion:**
Surgical and perioperative therapy textbook outcomes combined were achieved in more than half of the patients resected for PDAC in Germany. In the context of other international registries, the rate is comparable to hepatopancreatic surgery textbook outcome measures within the European Union or in the United States.

### Postoperative Outcomes after Liver Resections using the Comprehensive Complication Index: a Meta-Analysis

ID: 906

**Kategorie: DGAV - Hepato-pankreato-biliäre Chirurgie**

Ramin Raul Ossami Saidy[1], Nora Nevermann[1], Simon Moosburner[1], Sebastian Knitter[1], Felix Krenzien[1], Wenzel Schöning[1], Georg Lurje[1], Theresa Keller[1], Johann Pratschke[1], Nathanael Raschzok[1]

[1]*Charité Universitätsmedizin Berlin, Berlin, Deutschland*

**Background:**

Surgical resection remains a backbone in curative treatment of benign and malignant liver tumors as well as an important therapeutical option in patients with liver metastases. However, morbidity after liver resections remains significant with up to 40% of overall postoperative complications. Up to now, the "textbook" outcome after liver surgery has not been defined. The Comprehensive Complication Index (CCI) summarizes all postoperative complications and has been developed to serve as a standardized and widely applicable primary endpoint in surgical trials. We here aim to constitute benchmark outcomes after liver surgery by providing a meta-analysis of the CCI after liver resection in published clinical trials.

**Materials and methods:**

A systematic search was conducted using the following terms: "comprehensive complication index OR cci AND liver surgery". Randomized-controlled studies (RCT) and retrospective studies were considered, if they (i) reported the outcome after surgical liver resections, in (ii) adult patients, and (iii) used the CCI as postoperative outcome parameter. Studies reporting on liver transplantations were excluded. Meta-analysis was performed using a random-effects model and heterogeneity was assessed using $I^2$ statistics.

**Results:**

Overall, 347 studies were found and subsequently screened for eligibility. Out of these, 45 studies were included for further analysis, which reported on the postoperative course of 19,006 patients. Out of these, 89.7% were retrospective and 10.3% were prospective clinical trials. Regarding the surgical approach, 23 (33.8%) studies reported the outcome after laparoscopic surgery, 23 studies (33.8%) reported on open liver resections, and 18 studies (26.5%) included both approaches. Most studies included benign and malignant entities (n=39 / 57.4%). In meta-analysis, the overall mean CCI was 16.9 [95% Confidence Interval (CI): 16.2; 17.7, $I^2$=87.6% (84.8%; 89.9%)]. Regarding the surgical approach, the mean CCI was 10.6 (95% CI: 10.2; 10.9) after open liver resection and 12.7 [95% CI: 7.7; 17.7, $I^2$= 99.2% (99.1%; 99.3%)] after minimally liver resection. A total of 29 studies including 7,573 patients reported on mortality after liver surgery, with an overall number of 139 patients with a CCI of 100 (1.8%).

**Conclusion**

We here present a meta-analysis of the CCI after liver resection in published clinical trials, stratified for open and laparoscopic surgery. Our meta-analysis might provide a landmark for designing future clinical trials of liver resection.

**Comparison of postoperative pain between biological versus synthetic mesh six month after laparoendoscopic hernia repair – results of the randomized, multicenter, self-controlled BIOLAP trial**
ID: 110

**Kategorie: DGAV - Hernien und Bauchwandchirurgie**

C. Simone Seefeldt[1], Jürgen Meyer[1], Judith Knievel[2], Jonas Lange[1], Rolf Lefering[3], Markus Maria Heiss[1]

[1]*Klinik für Viszeral-, Tumor-, Transplantations- und Gefäßchirurgie, Klinikum der Universität Witten/Herdecke, Campus Merheim, Kliniken der Stadt Köln, Köln, Deutschland*, [2]*Zentrum für klinische Studien, Universität Witten/Herdecke, Witten, Deutschland*, [3]*Institut für Forschung in der Operativen Medizin , Universität Witten/Herdecke, Köln, Deutschland*

**Background:**
Inguinal hernia repair is one of the most common surgical operations globally; more than 20 million groin hernias are repaired annually worldwide. A remaining problem after hernia surgery is the occurrence of chronic pain. Up to now, the use of synthetic meshes is the standard procedure, but there is raising evidence that biological meshes could be advantageous concerning occurrence of chronic pain due to a different postoperative remodeling, without the disadvantages of a life-long implant.
The BIOLAP trial compares the use of synthetic and biological meshes in laparoendoscopic inguinal hernia repair. The use of biological meshes, which have been approved for many years, has been insufficiently researched.
To test the hypothesis that the use of biological meshes results in significantly less pain postoperatively than the use of synthetic mesh material, without an increased recurrence rate, patients with bilateral primary inguinal hernias were included. Patients received biological mesh on one side and synthetic mesh on the other (randomized, self-controlled study design with data monitoring).

**Materials and methods:**
In 21 German hernia centres, 982 hernias were treated using TEP or TAPP techniques. In addition to the study endpoints (recurrence rate within 2 years, reduction of pain after 6 months), demographic and perioperative data as well as intraoperative and postoperative complications were recorded. The data collection was carried out in a prospective study design with active patient follow-up.

**Results:**
Recruitment was successfully completed in February 2021 with inclusion of 491 patients. Presence of pain at screening and hospital discharge as well as after 1 week and 6 months are compared between both mesh types. Furthermore general demographics of the patients with bilateral primary inguinal hernias, the types of hernias included, the surgical method used (TEP/TAPP, mesh fixation, drainage) as well as intra- and postoperative complications (including haematoma, seroma) referred to the mesh material used are evaluated.

**Conclusion:**
The study provides multicentre data from 982 hernia operations dependent of the mesh material used. According to the study protocol, at the current time reduction of pain after 6 months comparing both mesh materials has been evaluated as the first endpoint of the trial. Further study endpoints (recurrences, patient satisfaction) will be evaluated after completion of the two-year follow-up phase.

**Register based analysis of robotic TAPP compared to endoscopic TEP for inguinal hernia repair**
ID: 293

**Kategorie: DGAV - Hernien und Bauchwandchirurgie**

Robert Christian Bauer[1], Raffaele Galli[1], Bernd Schenkluhn[1], Dietmar Eucker[1], Robert Rosenberg[1], Sebastian H. Lamm[1]

[1]*Kantonsspital Baselland, Klinik für Allgemein-, Viszeral-, Gefäss- und Thoraxchirurgie, Liestal, Schweiz*

**Background:**
Robotic procedures are becoming more and more popular in minimally invasive hernia surgery. The DaVinci rTAPP (robotic transabdominal preperitoneal inguinal hernia repair) was implemented in autumn 2018 in our department. In our institution, the standard non-robotic technique is the TEP procedure (totally extraperitoneal repair) (54% of all inguinal hernia operations). Since July 2017, 75% of hernia repairs were done by TEP, rTAPP or conventional TAPP, whereas only 25% were open operations (mainly Lichtenstein technique). We present short term and one-year follow-up results for patients undergoing rTAPP compared to endoscopic TEP for inguinal hernia repair at our institution.

**Materials and methods:**
From end of 2018, a DaVinci SI robotic system was used until it was replaced in July 2021 by a XI system. Patients undergoing rTAPP or TEP operations were prospectively entered into the multicenter, internet-based international (German speaking countries) Herniamed Hernia Registry). Preoperative assessment including pain score and risk factors was performed and a standardized one-year follow-up questionnaire was sent to the patients. Light-weight, nonabsorbable mesh was used for all minimally invasive procedures. Operation time and multiple other perioperative parameters were recorded, according to the standards of the Herniamed registry.

**Results:**
From July 2017 – December 2021, a total of 1095 inguinal hernia operations performed at our institution were entered into the Herniamed Registry. Among them, 823 (75%) were done by minimally invasive approach (rTAPP: n=129 (16%), TAPP n=123 (15%), TEP n=571 (69%). Emergency operations were mainly performed as TAPP (21 out of 26, 81%). Comparison of the rTAPP group versus TEP group showed significantly higher age (mean±SD, 61.1±13.5 years vs. 56.5±15.5 years, $p<0.001$) and BMI (mean±SD, 26.1±3.8 kg/m² vs. 25.1±3.1 kg/m², p=0.006) in the rTAPP group. Bilateral operation was performed in 46.5% (60/129) of rTAPP operations vs. 36% (206/571) of TEP operations (p=0.035). There was no statistically significant difference in gender, ASA (American society of Anesthesiology) score and risk factors between unilateral rTAPP and unilateral TEP, however the bilateral rTAPP subgroup included more ASA 3 patients than bilateral TEP group (11/60 (18.3%) vs. 16/206 (7.8%), p=0.027). Large sized bilateral inguinal hernias (EHS III, European Hernia society) were treated in 19 out of 60 bilateral rTAPP cases as compared to only 14 EHS III Hernias in 206 bilateral TEP cases (31.7 % vs. 6.8 %, $p<0.001$). Recurrent Hernia was present in 21.7% of patients scheduled for bilateral rTAPP and in 8.7% of patients who received bilateral TEP (p=0.011). Operation time was significantly longer in rTAPP as compared to TEP, both for unilateral hernias (mean±SD, 76.8±26.7 min vs. 52.4±20.3 min, $p<0.001$) and bilateral hernias (115.5±29.4 min vs. 71.8±25.3 min, $p<0.001$). Intra- and postoperative complication rates were low both for rTAPP and TEP and there were no statistically significant differences [ (intraoperative complication rate for unilateral rTAPP 1.4% vs. unilateral TEP 1.6% (p=1); postoperative complication rate for unilateral rTAPP 0% vs. unilateral TEP 1.1% (p=1); intraoperative complication rate for bilateral rTAPP 0.5% vs. bilateral TEP 0% (p=1); postoperative complication rate for bilateral rTAPP 1.7% vs. bilateral TEP 0.5% (p=0.401) ]. One-year follow-up was completed in 36 patients with unilateral rTAPP and 251

patients with unilateral TEP, as well as 32 patients with bilateral rTAPP and 137 patients with bilateral TEP. Recurrence rate after one year was 0% for both unilateral and bilateral rTAPP, 1.6% for unilateral TEP and 4.4% for bilateral TEP. There was no statistically significant difference in recurrence rate between rTAPP and TEP. Pain at rest was reported by 2.8% of patients after unilateral rTAPP, and 6% after unilateral TEP (p=0.703). Pain at rest was present in 3.2% of patients after bilateral rTAPP, and 7.3% after bilateral TEP (p=0.692).

**Conclusion:**
TEP and rTAPP are the minimally invasive operation techniques of choice at our institution for elective cases. Conventional TAPP was preferred for emergency operations. There was still significantly longer operation time for rTAPP than TEP. However, rTAPP patients were significantly older and had higher BMI. Bilateral rTAPP was performed on more ASA 3 patients, more EHS III Hernias and more recurrent hernias. We found very low complication rate in both TEP and rTAPP techniques. The recurrence rate after one year in TEP was low, and we have not observed recurrences after rTAPP yet. Pain level at one-year follow-up was lower after rTAPP than TEP, even if the difference was not statistically significant. In conclusion, rTAPP is safe and follow up data show comparable results between the two techniques.

**Brauchen wir eine neue Nahttechnik beim Verschluss der abdominellen Faszie ?**
ID: 524

**Kategorie: DGAV - Hernien und Bauchwandchirurgie**

Resit Demir[1]

[1]*Medizinisches Versorgungszentrum Dr. R. Demir und Kollegen, Chirurgische Universitätsklinik Erlangen-Nürnberg, Nürnberg, Deutschland*

**Background:**
The evolution of suturing techniques for the abdominal fascia appears to have ended with these two suturing techniques: interrupted or continues Next step to strengthen the fascia is the recommendation to insert mesh. In the following work, another third suture technique for the closure of an abdominal fascia is described, which improves on the previous suture techniques.

**Materials and methods:**
Between 2014 and 2020, one surgeon performed the "spider suture technique" for umbilical hernias, epigastric hernias and selected incisional hernias in 102 patients. In addition to the continuous suture, the spider suture includes several individual, circular threads that are knotted with itself. This ensures that the fascia is prevented from being torn out due to poor quality of the abdominal fascia or the increased repetitive pressure on the fascia.

**Results:**
Ninety-seven patients were reached. Most patients n=75 had a primary hernia (umbilical or epigastric). More than fifth of the patients had a secondary abdominal wall hernia n=22. Of these, 19 were median and 3 were paramedian. The median follow up was 30 months. A recurrence was not found either in the patients with primary or secondary abdominal wall hernia with median incision. One patient with paramedian incisional hernia showed a recurrence.

**Conclusion:**
The spider suture is an effective new suturing technique to close the abdominal fascia after primary abdominal wall hernias. To what extent this next step in the evolution of the abdominal wall suture technique is suitable for secondary abdominal wall hernias or primary abdominal wall closure after laparotomy will have to be proven in further studies.

**Excellent three-year results with incisional hernia repair based on biomechanical considerations**
ID: 709

**Kategorie: DGAV - Hernien und Bauchwandchirurgie**

Regine Nessel[1], Johannes Rinn[2], Thorsten Löffler[3], Friedrich Kallinowski[4]

[1]*SLK Klinikum am Gesundbrunnen, Allgemein-, Visceral- und Kinderchirurgie, Heilbronn, Deutschland*, [2]*Kreiskrankenhaus Bergstrasse, Allgemein- und Visceralchirurgie, Heppenheim, Deutschland*, [3]*GRN Klinik, Allgemein- und Visceralchirurgie, Eberbach, Deutschland*, [4]*Universitätsklinikum Heidelberg, General, Visceral and Transplantation Surgery, Heidelberg, Deutschland*

**Background:**
Incisional hernia repair is frequently followed by recurrences and chronic pain. The calculation of mechanical stability (GRIP) as a dimensionless measure has been applied clinically to 228 patients (Kallinowski et al. Front. Surg. 4:78. doi: 10.3389/fsurg.2017.00078, 2018; Front Surg 8:602181, 2021; doi: 10.3389/fsurg.2021.602181). How are the three-year results?

**Materials and methods:**
Meshes and fixation materials were subjected to low cyclic loading in a self-built bench test. Meshes with the highest stability were used in four hospitals to reach sufficient grip values for the given defect size. Clinical data, pain levels and recurrence rates were monitored for three years in the HERNIAMED®/ STRONGHOLD registry.

**Results:**
A total of 129 patients (61 female, 68 male) with an average age of 61 ± 13 years (Range: 27 – 92) were included. Mean BMI was 29 ± 6. Primary repairs were done in 93 patients. Recurrences were repaired in 36 patients after 1 to 4 years. Mean OR time was 172 ± 88 min. At an average hernia size of 9 x 13 cm, Dynamesh® Cicat was used in 76 patients. Progrip® was utilized in 53 cases. The average overlap was 5 ± 2 cm. The mean mesh: defect area ratio (MDAR) was 12 ± 17 (median 8). The average grip value was calculated as 228 ± 190 (median 198). Using Progrip® , higher MDAR but lower GRIP was achieved. Higher grip values at larger hernia sizes are a consequence of more fixation. Recurrences or chronic pain were absent in both meshes after three year.

**Conclusion:**
With the GRIP concept, biomechanically stable incisional hernia repair is possible. Biomechanically stable repairs exhibit low recurrence rates and no chronic pain after three years.

### The area of the unstable abdominal wall should be considered for complex incisional hernia repair to achieve low recurrence rates after two years

ID: 710

**Kategorie: DGAV - Hernien und Bauchwandchirurgie**

Friedrich Kallinowski[1], Jacob Jonas Relle[2], Philipp Lösel[2], Samuel Voss[3], Vincent Heuveline[2], Matthias Vollmer[4], Johannes Görich[5], Mark O. Wielpütz[6], Regine Nessel[7]

[1]*University Hospital Heidelberg, General, Visceral and Transplantation Surgery, Heidelberg, Deutschland*, [2]*Engineering Mathematics and Computing Lab , Interdisciplinary Center for Scientific Computing, Heidelberg, Deutschland*, [3]*Laboratory of Fluid Dynamics and Technical Flows, Otto-von-Guericke University Magdeburg, Magdeburg, Deutschland*, [4]*Hamburg University of Technology, Biomechanics, Hamburg, Deutschland*, [5]*Radiological Center, Eberbach, Deutschland*, [6]*Diagnostic and Interventional Radiology, University Hospital Heidelberg, Heidelberg, Deutschland*, [7]*General, Visceral and Pediatric Surgery, SLK Klinikum Am Gesundbrunnen, Heilbronn, Deutschland*

**Background:**
Recurrences and chronic pain are frequent consequences of complex incisional hernia repair. Recurrence rates and pain levels can be reduced if the area of the unstable abdominal wall is stabilized. Can computerized tomography of the abdomen with Valsalva's maneuver determine the required size of the hernia mesh?

**Materials and methods:**
From March 2019 up to July 2020, a total of 24 patients with incisional hernias more than 10 cm wide were investigated prior to hernia repair using computerized tomography of the abdomen with Valsalva's maneuver. Included were 12 primary and 12 recurrent repairs with 42 risk factors for recurrence with 7 being immunosuppressed and 6 transplant recipients. Grip values necessary for a biomechanically stable incisional hernia repair were calculated as previously described (Kallinowski et al. Front. Surg. 4:78. doi: 10.3389/fsurg.2017.00078, 2018; Front Surg 8:602181, 2021; doi: 10.3389/fsurg.2021.602181). The area of the unstable abdominal wall was determined at least in triplicate by hand and sixfold with computer aid from changes of the defect area using previously published models setting the cut-off at a ventral shift of the abdominal wall to 1.5 cms (Lösel et al. Nat Commun 11: 5577, 2020. Doi: 10.1038/s41467-020-19303-w, Voß et al., Current Directions in Biomedical Engineering 2020 6: 20203024 doi.org/10.1515/cdbme-2020-3024).

**Results:**
The average defect area assessed clinically was 258 ± 146 cm² (median 217 cm², average width and length: 15 x 21 cm). The area of the abdominal wall instability was 487 ± 420 cm², median: 396 cm²). All cases were repaired with CICAT® hernia mesh (30 x 45 cm up to 49 x 49 cm, average mesh size 1078 ± 379 cm², median: 1060 cm², average overlap 5.7 ± 1.6 cm, median 5.25) in a retromuscular position. A posterior release was necessary in all cases, combined with a sandwich repair in 5 cases. Mesh-defect area ratio was 5.1 ± 2.2 (median: 4.9). Strain values for each individual were graphically depicted. Hot spots were detected and fixation elements were placed accordingly (average number of fixation points ± SD: 134 ± 71, median 118). After following all patients for three years, no recurrence and no chronic pain occurred.

**Conclusion:**
Computerized tomography with Valsalva's maneuver can assess the area of the unstable zone of the abdominal wall prior to grip-based incisional hernia repair. The evaluation process by artificial intelligence and non-rigid b-spline registration takes 5 – 10 min. The evaluation per hand takes 20 – 45 min per observer. The new algorithms speed the process up and make it useful for routine clinical work. The clinical results after two years are excellent.

**Epiploic appendagitis: an uncommon and a nonsurgical cause of abdominal pain**
ID: 373

**Kategorie: DGAV - Koloproktologie**

Ahmad Alhazmi[1], Claudia Seifarth[1]

[1]*Charité – Universitätsmedizin Berlin Campus Benjamin Franklin, Klinik für Allgemein- und Viszeralchirurgie, Berlin , Deutschland*

**Background:**
Epiploic appendagitis (EA) is an uncommon cause of abdominal pain and due to its non-specific presentation and its rare occurrence often misdiagnosed with other common causes of acute abdomen, such as acute appendicitis, cholecystitis or diverticulitis. Thereby that lead patients to undergo unnecessary surgeries. The treatment is primarily conservative with analgesics and sometimes combined with nonsteroidal anti-inflammatory drugs (NSAIDS). Surgery is sometimes required by persistence or worsening of symptoms. In this Work, we aim to provide a clinical course of the disease, typical radiological feature and to emphasize the importance of the early diagnosis of EA.

**Materials and methods:**
Retrospective review of 40 patients diagnosed with epiploic appendagitis between January 2015 and September 2022 at Charité University Hospital Berlin, Campus Benjamin Franklin. The medical records were reviewed in regards to clinical und radiological feature, anatomical location of the EA und treatment methods.

**Results:**
40 patients (29 male ,11 female) were diagnosed with EA. Almost all the patients present with abdominal pain except in 6 patients (15 %), the diagnosis was as incidental finding in CT. Specific findings in CT with typical picture of EA were found in 31 patients ( 77,5 %). The location of the EA were mostly in the left side of the colon: 12 were found in sigma (30 %), 13 in descending colon (32.5 %), 5 in descend-sigmoidal transition (12.5 %). The rest were found as the following: 5 in ascending colon, 1 in cecum, 1 transverse colon, 1 in the right flexure of the colon, 2 unclear location in the colon. 26 patients (65 %) were admitted for conservative therapy and the rest of the patients were treated as outpatient. No patients underwent surgery and all of them treated with analgesics with (NSAIDS) und some patients became in addition antibiotic therapy.

**Conclusion:**
Epiploic appendagitis is an important deferential diagnosis in patients with acute abdomen that can mimic other diagnosis. Based on disease-nature and its non-operative therapy, the early and accurate diagnosis of EA with is computerized tomography (CT) is important to avoid unnecessary surgery.

**Role of visceral fat on postoperative outcome and recurrence in Crohn's disease patients**
ID: 479

**Kategorie: DGAV - Koloproktologie**

Ioannis Pozios[1], David Kaufmann[2], Katharina Boubaris[1], Antonis Margonis[3], Katharina Beyer[1], Claudia Seifarth[1], Johannes Lauscher[1]

[1]*Charité - Universitätsmedizin Berlin, Klinik für Allgemein- und Viszeralchirurgie, Berlin, Deutschland*, [2]*University Hospital Augsburg, Department of Diagnostic and Interventional Radiology and Neuroradiology, Augsburg, Deutschland*, [3]*Memorial Sloan Kettering Cancer Center, Department of Surgery, New York, United States*

**Background:**
Body fat distribution has been proposed to be prognostic factors of outcomes for various diseases, but its exact role in Crohn's disease (CD) is unknown. The aim of this study is to assess the impact of visceral and subcutaneous fat on postoperative complications and CD recurrence after bowel resection in patients with CD.

**Materials and methods:**
A retrospective analysis of CD patients undergoing ileocecal resection at our tertiary referral center was performed. Patient demographics, operative outcomes and recurrence rates were recorded. Total fat area (TFA), visceral fat area (VFA), subcutaneous fat area (SFA), and abdominal circumference (AC) were measured using an established image analysis method at third lumbar vertebra level on MRI cross-sectional images. Additionally, ratios of the values above were calculated.

**Results:**
A total of 347 patients were retrospectively analyzed. An adequate abdominal MRI scan within 12 months prior to surgery was available for 223 patients with median follow-up time of 48.8 months (IQR: 20.0–82.9). BMI correlated with SFA ($p = 0.0001$), VFA ($p = 0.0001$) and AC ($p = 0.0001$). High ratio of VFA/TFA was associated with prolonged hospital stay ($p = 0.034$). None of the MRI-based variables (VFA, SFA, VFI, VFA/TFA) predicted anastomotic leakage, any surgical morbidity or CD recurrence ($p > 0.05$).

**Conclusion:**
High ratio of viszeral fat area was associated with prolonged hospital stay after bowel resection for CD. None of the radiological obesity variables are reliable predictors of postoperative outcomes or recurrence in CD patients undergoing ileocecal resection. Conventional clinical adiposity markers such as BMI seems to be sufficient to preoperative stratification of obese patients to optimize high-risk surgical patients and improving outcomes.

**Ghost-ileostomy versus conventional loop ileostomy in patients undergoing low anterior resection for rectal cancer: a randomised controlled multicentre trial**
ID: 735

**Kategorie: DGAV - Koloproktologie**

Felix J. Hüttner[1,2], Pascal Probst[2,3], André Mihaljevic[1,2], Colette Dörr-Harim[1,2], Alexis Ulrich[2,4], Markus Diener[2,5], Phillip Knebel[2]

[1]*Universitätsklinikum Ulm, Klinik für Allgemein- und Viszeralchirurgie, Ulm, Deutschland*, [2]*Universitätsklinikum Heidelberg, Klinik für Allgemein-, Viszeral- und Transplantationschirurgie, Heidelberg, Deutschland*, [3]*Spital Thurgau AG, Chirurgische Klinik, Thurgau, Schweiz*, [4]*Lukaskrankenhaus Neuss, Chirurgische Klinik I, Neuss, Deutschland*, [5]*Universitätsklinikum Freiburg, Klinik für Allgemein- und Viszeralchirurgie, Freiburg, Deutschland*

**Background:**
Anastomotic leakage is the most important complication after colorectal surgery. Thus, in most patients undergoing low anterior resection with total mesorectal excision a diverting loop ileostomy is fashioned to protect the low anastomosis. Ghost ileostomy is a pre-stage ileostomy, which can be converted to a diverting ileostomy if anastomotic leakage is suspected, thus avoiding the severe consequences of anastomotic leakage. On the other hand, if there are no signs of anastomotic leakage, an actual ileostomy can be avoided. The aim of the current pilot trial was to evaluate whether ghost ileostomy is a safe alternative to the current reference standard of conventional loop ileostomy.

**Materials and methods:**
A randomised controlled pilot trial comparing ghost ileostomy to conventional loop ileostomy in patients undergoing low anterior resection with total mesorectal excision for rectal cancer was performed. Patients were randomised intraoperatively after the anastomosis was fashioned and only if no intraoperative contraindications were present. All patients were followed-up at regular intervals for 6 months postoperatively. The comprehensive complication index was the main outcome parameter. Furthermore, specific complications, frequency of transformation of ghost ileostomy into conventional ileostomy, the proportion of patients with an ostomy at six months after index surgery and patient-reported outcomes in terms of the Wexner Score and quality of life questionnaires (EORTC QLQ-C30 & CR29) were assessed.

**Results:**
The trial was started as a single centre trial in May 2018. Due to slow recruitment, the trial was expanded into a multicentre design with two additional centres. Finally, the trial was prematurely stopped after accrual of 30 patients in September 2022 due to ongoing slow recruitment despite adjustments of the trial design. There was no 90-day mortality and no patient had to undergo a Hartmann's procedure (terminal ostomy creation). An actual ileostomy could be avoided in eight of the 15 (53.3%) patients in the ghost ileostomy group, while the ghost ileostomy was exteriorised in 7 (46.7%) patients. At the time of abstract submission, the last two patients are still followed-up for the 3- and 6-month results. Full results including the primary and all secondary endpoints will be presented at the conference.

**Conclusion:**
Ghost ileostomy seems to be a valuable tool in the armamentarium of colorectal surgeons in order to avoid an ileostomy in a reasonable proportion of patients undergoing low anterior resection with total mesorectal excision for rectal cancer. The current results are promising, but the feasibility was limited due to the eligibility criteria and surgeons' reluctance. The potential risks and benefits of the procedure should be discussed with eligible patients to provide them with the chance of avoiding an ostomy after rectal surgery.

**Hiatus hernia repair with a new-generation biosynthetic mesh. A 4-year single-center experience.**
ID: 71

**Kategorie: DGAV - Minimalinvasive Chirurgie**

Kristjan Ukegjini[1,2], Diana Vetter[2], Valerian Dirr[2], Christian A. Gutschow[2]

[1]*Kantonsspital St. Gallen, Department of General, Visceral, Endocrine and Transplant Surgery, St. Gallen, Schweiz*, [2]*University Hospital Zürich, Department of Visceral and Transplant Surgery, Zürich, Schweiz*

**Background:**
Mesh augmentation is a highly controversial adjunct of hiatus hernia (HH) surgery. The current scientific evidence remains unclear and even experts disagree on indications and surgical techniques. With an aim to avoid the downsides of both non-resorbable synthetic and biological materials, biosynthetic long-term resorbable meshes (BSM) have recently been developed and are becoming increasingly popular. In this context, we aimed at assessing outcomes after HH repair with this new generation of mesh at our institution

**Materials and methods:**
From a prospective database, we identified all consecutive patients that underwent HH repair with BSM augmentation. Data was extracted from electronic patient charts of our hospital information system. Endpoints of this analysis included perioperative morbidity, functional results and recurrence rates at follow-up.

**Results:**
Between December 2017 and July 2022, 97 patients (elective primary cases n=76, redo cases n=13, emergency cases n=8) underwent HH with BSM augmentation. Indications in elective and emergency cases were paraesophageal (Type II-IV) HH in 83%, and large Type I HH in 4%. There was no perioperative mortality, and overall (Clavien-Dindo ≥2) and severe (Clavien-Dindo ≥3b) postoperative morbidity was 15% and 3%, respectively. Textbook outcome without postoperative complications was achieved in 85% of cases (elective primary surgery 88%, redo cases 100%, emergencies cases 25%). After a median (IQR) postoperative follow-up of 12 months, 69 patients (74%) were asymptomatic, 15 (16%) reported improvement, and 9 (10%) had clinical failure, of which 2 patients (2%) required revisional surgery.

**Conclusion:**
Our data suggest that HH repair with BSM augmentation is feasible and safe with low perioperative morbidity and acceptable postoperative failure rates at early to mid-term follow-up. BSM may be a useful alternative to non-resorbable materials in HH surgery.

**Robot-assisted minimally-invasive splenectomy - selective dissection of the hilar structures**
ID: 181

**Kategorie: DGAV - Minimalinvasive Chirurgie**

Thilo Welsch[1], Benjamin Müssle[1], Florian Corvinus[1], Helmut Linster[1], Alina Lesan[1]

[1]*Klinik für Allgemein-, Viszeral- und Thoraxchirurgie, St. Elisabethen-Klinikum Ravensburg, Ravensburg, Deutschland*

**Background:**
Probably, robotic and laparoscopic splenectomy are equal in terms of safety, conversion rate and hospital stay. Robotic splenectomy (RS) may lead to less intraoperative blood loss. Here, we demonstrate that RS allows a fine selective dissection of the hilar vessels and safe preservation of the pancreatic parenchyma in a series of RS for young patients.

**Materials and methods:**
A retrospective analysis of elective RS cases between June and September 2022 at a single center. Elective RS was indicated for different localized intrasplenic cystic or solid lesions. An intraoperative video of the procedure was captured and will be presented. The patients were placed in a French position and operated using the DaVinci Xi system. A concise review of the literature on RS is further provided.

**Results:**
RS was performed in 3 patients (2 female) aged 20, 20, and 30 years for large epithelial cysts (n=2) or littoral cell angioma, respectively. The operative time was 162–207 min (range) and there was no documented blood loss or postoperative morbidity in any case. The postoperative hospital stay was 4 days. The operative approach started with division of the gastrocolic and gastrosplenic ligament and mobilization of the left colic flexure if needed. The peritoneum to open the greater sac towards the diaphragm and the lateral peritoneal folds were divided. Next, the selective dissection and ligation of the hilar vessels was performed under preservation of the adjacent pancreas under close visual control.
The spleen was harvested in a specimen bag and removed using a Pfannenstiel incision.

**Conclusion:**
The video exemplifies high quality visualization and vascular control during minimally-invasive selective hilar dissection for elective splenectomy in a series of young patients. RS resulted in a favorable postoperative outcome with no blood loss, no complications and a fast recovery.

**Minimally invasive emergent esophagectomy for a longitudinal esophageal perforation**
ID: 212

**Kategorie: DGAV - Minimalinvasive Chirurgie**

Emrullah Birgin[1], Christoph Reißfelder[1], Nuh N. Rahbari[1]

[1]*University Hospital Mannheim, Medical Faculty Mannheim, Heidelberg University, Department of Surgery, Mannheim, Deutschland*

**Background:**
Esophageal rupture is a life-threatening condition and requires urgent diagnostic and effective management. Treatment options include emergent surgical and endoscopic techniques while open conventional esophagectomy has been traditionally performed in septic patients. A minimally invasive approach has been rarely used in the emergency setting with mediastinal contamination.

**Materials and methods:**
We herein report a case of a 68-year-old male undergoing minimally invasive esophagectomy for a longitudinal esophageal rupture after a failure of an endoscopic stent treatment. A video of thoracoscopic esophagectomy was demonstrated.

**Results:**
The patient was admitted to our hospital for an endoscopic submucosal dissection of an early gastrointestinal stroma tumor of the stomach. During the endoscopic procedure, the patient suffered an iatrogenic longitudinal rupture of the esophagus with upfront endoscopic stent placement. However, the patient developed a septic shock after the initial management and an emergent surgical treatment was mandatory. The patient underwent a minimally invasive esophagectomy in a prone position with extraction of the stent thoracoscopically. The total operating time was 300 min. The postoperative course was uneventful, and the patient was discharged on postoperative day 10 after completion of the antibiotic treatment.

**Conclusion:**
Minimally invasive esophagectomy is feasible in the emergency setting for patients with longitudinal rupture of the esophagus**.**

**PRAY – a prospective, observational study for assessing risk factors for intestinal anastomosis leakage**
ID: 697

**Kategorie: DGAV - Minimalinvasive Chirurgie**

Maximilian Denzinger[1], Colette Doerr-Harim[1], Lisa Hunold[1], Maren Brenk[1], Christoph Michalski[1], Marko Kornmann[1], Benno Traub[1]

[1]*Universitätsklinikum Ulm, Klinik für Allgemein- und Viszeralchirugie, Ulm, Deutschland*

**Background:**
With nearly 16 million surgeries performed in Germany in 2020 and almost 17.000 oncologic surgeries, the reduction of the perioperative complication rate has high medical and economical relevance (1). Intestinal anastomotic leakage is one of the most relevant complications in the field of visceral and general surgery with high impact on the mortality of patients undergoing surgery (2).
In patients undergoing major abdominal surgery, an activation of pro- and anti-inflammatory cells and cascades resulting in an alteration of adaptive and innate immunity can be observed (3). The patient's response towards this trauma is highly individual. While some patients present lightly elevated inflammatory parameters, others, undergoing the same surgery, show a massive immune dysregulation and activation leading to conditions comparable to septic shock called the systemic inflammatory response syndrome (SIRS) (4). Patients with the clinical presentation of SIRS show higher mortality and morbidity after surgical trauma (5).
Yet, we are not able to detect patients with a higher risk for developing SIRS before surgery and therefore cannot intervene early and adequately. Malignant disease has been shown to severly alter the patients immune system and thus we believe that especially tumor patients would benefit from a preoperative risk stratification to minimize perioperative complications (6).
We hypothesize that a subset of patients already displays dysregulations of their immune system preoperatively. These dysfunctions impair physiologic wound healing and can lead to outright SIRS.

**Materials and methods:**
We conducted a translational-clinical study to establish a biomarker panel to predict anastomotic leakage in patients undergoing visceral surgery with at least one intestinal anastomosis. Additional perioperative complications such as thromboembolic /ischemic complications, surgical site infections and organ failure are evaluated as secondary endpoints. The study was registered at the DRKS (Deutsches Register klinischer Studien) with the register number: DRKS00029194. The first patient was enrolled in the study in August 2022.
Previous studies in patients with perioperative complications showed high anti- and pro-inflammatory mediators after surgery. We believe this immunologic imbalance exists even before surgery and could therefore be modified. In detail we analyze leukocyte subpopulations by flow cytometry such as immunosuppressive macrophages, regulatory T-cells, myeloderived suppressor cells (MDSC) and surface markers involved in transmigrations processes as well as circulating immune mediators, such as Interleukin-6 and Interleukin-8 by ELISA and their association with perioperative complications. Blood tests will be performed preoperatively and on post-operative days (POD) 1, 3, and 5. Further patients will be visited on the postoperative days 1, 3 and 5 to clinically assess the occurrence of an anastomotic leakage, wound healing disorder and other complications. Assessed complications are graded according to the Classification of Clavien-Dindo and documented in an electronical case report file (eCRF) using the RedCap System. A total of 500 patients shall be included in the next two years.

**Results:**
Up to now we are able to present preliminary results of the ongoing study. After recruiting in the beginning of September we included 11 patients. Most patients were treated for malignant diseases and received robotic assisted, minimally invasive tumor resection. In the next six months we plan to include up to 100 patients and therefore we will present first preliminary results of our translational study.

**Conclusion:**
In the future we hope to be able to stratify each patient’s individual risk score for perioperative complications. After identification of individual risk profiles, we will focus on therapeutic strategies for modification of these risk factors, hopefully resulting in an interventional study to reduce the risk for perioperative complications.

**References:**
[1] Diers, J.; Acar, L.; Baum, P.; Flemming, S.; KastnerAU - Germer, C.; Germer, C.T.; L'hoest, H.; Marschall, U.; Lock, J.F.; Wiegering, A. Fewer Operations for Cancer in Germany during the First Wave of COVID-19 in 2020. Dtsch. Arztebl Int. 2021, 118, 481-482.
[2] Baum, P.; Diers, J.; Lichthardt, S.; Kastner, C.; Schlegel, N.; Germer, C.T.; Wiegering, A. Mortality and Complications Following Visceral Surgery: A Nationwide Analysis Based on the Diagnostic Categories used in German Hospital Invoicing Data. Dtsch. Arztebl Int. 2019, 116, 739-746.
[3] Dabrowska, A.M.; Slotwinski, R. The Immune Response to Surgery and Infection. Cent. Eur. J. Immunol. 2014, 39, 532-537.
[4] Hotchkiss, R.S.; Monneret, G.; Payen, D. Sepsis-Induced Immunosuppression: From Cellular Dysfunctions to Immunotherapy. Nature Reviews Immunology 2013, 13, 862-874.
[5] Talmor, M.; Hydo, L.; Barie, P.S. Relationship of Systemic Inflammatory Response Syndrome to Organ Dysfunction, Length of Stay, and Mortality in Critical Surgical Illness: Effect of Intensive Care Unit Resuscitation. Archives of surgery 1999, 134, 81-87.
[6] Kawakami, Y.; Qian, L.; Kawamura, N.; Miyazaki, J.; Tsubota, K.; Kinoshita, T.; Nakamura, K.; Ohmura, G.; Satomi, R.; Sugiyama, J. Cancer Induced Immunosuppression and Its Modulation by Signal Inhibitors. In Resistance of Cancer Cells to CTL-Mediated Immunotherapy.; Anonymous .; Springer, 2015, pp. 287-301.

### Transdiaphragmatic herniation after robot-assisted minimally invasive esophagectomy (RAMIE)

ID: 60

**Kategorie: DGAV - Ösophagus- und Magenchirurgie**

Stefanie Brunner[1], Dolores Thea Müller[1], Jennifer Eckhoff[1], Valentin Lange[2], Thomas Schmidt[1], Wolfgang Schröder[1], Christiane J. Bruns[1], Hans Fuchs[1]

[1]*University Hospital of Cologne, Department of General, Visceral, Cancer and Transplantation Surgery, Cologne, Deutschland*, [2]*University of Cologne, Köln, Deutschland*

**Background:**

Diaphragmatic transposition of intestinal organs is a major complication after esophagectomy with a significant mortality and morbidity. However, in the current literature, treatment procedures and outcomes are not evaluated for patients who received a robot-assisted Ivor Lewis minimally invasive esophagectomy (IL- RAMIE). The aim of this study was to evaluate the outcomes for this patient cohort and to determine whether using a minimal invasive approach in the management of patients with a diaphragmatic hernia after an IL-RAMIE procedure is safe and feasible.

**Materials and methods:**

This study includes all patients who developed a postesophagectomy hernia after IL-RAMIE at our high volume center between April 2017 and December 2021. The analysis focuses on time to herniation, symptoms, chosen operation procedure, and follow up treatments for these patients.

**Results:**

A total of 145 patients underwent an IL-RAMIE in the study period. 9 patients (6,2 %) developed a postesophagectomy hernia. Patients presented with symptoms like reflux, nausea, vomiting and pain. However, one third of the patients didn’t suffer from any symptoms. In all cases, a CT scan was performed in which the colon transversum always presented as the herniated organ (see Figure 1). In one patient, a herniation of the small intestine, pancreas and omentum majus occurred additionally. 78% of these patients were operated in a minimally invasive manner with a mean hospital stay of 8 days. No severe adverse events occurred.

**Conclusion:**

This study shows that transdiaphragmatic herniation is an underestimated problem. A minimally invasive approach in repositioning a postesophagectomy hernia after IL-RAMIE is safe and effective.

### Assessment of the effect of nitroglycerine and hypervolemia on gastric tube microperfusion by ICG fluorescence imaging in a porcine model

ID: 134

**Kategorie: DGAV - Ösophagus- und Magenchirurgie**

Philipp Holger von Kroge[1], Detlef Russ[2], Henrik Rieß[3], Eike Debus[3], Hans Pinnschmidt[4], Jakob Izbicki[1], Oliver Mann[1], Sabine Wipper[5], Anna Duprée[1]

[1]*Universitätsklinikum Hamburg-Eppendorf, Klinik für Allgemein-, Viszeral- und Thoraxchirurgie, Hamburg,*, [2]*Institut für Lasertechnologien in der Medizin und Meßtechnik an der Universität Ulm*, [3]*Universitätsklinikum Hamburg-Eppendorf, Klinik für Gefäßmedizin, Hamburg, Deutschland*, [4]*Universistätsklinikum Hamburg-Eppendorf, Insititut für Biometrie und Epidemiologie, Hamburg, Deutschland*, [5]*Medizinische Universität Innsbruck, Universitätsklinik für Gefäßchirurgie, Österreich*

**Background:**
Impaired microperfusion of the gastric tube is a significant risk factor for anastomotic leakage in patients requiring esophagectomy. Furthermore, the influence of hypervolemia and intraoperative administration of nitroglycerine on gastric tube microperfusion remains unclear, whereas hypervolemia is associated with increasing mortality in patients undergoing major pancreatic surgery.
Fluorescence imaging using indocyanine green (ICG-FI) and its quantification is a promising tool for intraoperative microperfusion evaluation. In previous studies, we could demonstrate that quantification of ICG-FI can sufficiently predict gastric tube microperfusion. Moreover, reduced fluorescence was associated with higher rates of anastomotic leakage.
In the present study we aimed to investigate the influence of administration of nitroglycerin and hypervolemia in different hemodynamic settings on gastric tube microperfusion by quantifying ICG-FI.

**Materials and methods:**
Three groups with seven pigs each were formed using noradrenaline, nitroglycerin, and hypervolemia for hemodynamic management, respectively. After median laparotomy, a gastric tube was created in each pig.
A total of three measurement times were set, which were determined on the basis of the mean arterial blood pressure (MAP): Hypotension (MAP < 60mmHg), normotension (MAP 60-90mmHg), and hypertension (MAP >90mmHg). Each measurement was carried out in three predefined regions of the gastric tube: the fundus, corpus, and prepyloric area. ICG-FI, hemodynamic parameters and transit-time flow measurement in the right gastroepiploic artery were continuously assessed. Fluorescent microspheres (FM) were administered, and the partial pressure of tissue oxygen was quantified. After finishing the experiments, the gastic tubes were processed histologically for quantification of tissue damage.
Quantification of ICG-FI was performed using customized software. The parameters were calculated from a time-dependent fluorescence intensity cure: Slope of fluorescence intensity (SFI), background-subtracted fluorescence intensity (BSFI) and time to slope (TTS).

**Results:**
Administration of nitroglycerine and hypervolemia were both associated with significantly impaired microperfusion compared to the noradrenaline group quantified by ICG-FI. The results significantly correlated with the FM measurement, which represents the gold standard for experimental perfusion measurement. In contrast to ICG-FI, the use of tissue oxygenation and TTFM for perfusion measurement, the changes in microperfusion could not be comprehended.

**Conclusion:**
Our results support current clinical practice with restrictive volume administration and catecholamine administration in major surgery. Hypervolemia should be avoided in the setting of esophagectomy with gastric tube pull up. Furthermore, continuous administration of nitroglycerine is not suitable for improving microperfusion of the gastric tube.

**Management of Paraesophageal Hiatus Hernia: Recommendations following a European Expert Delphi Consensus**
ID: 161

**Kategorie: DGAV - Ösophagus- und Magenchirurgie**

Stephan Gerdes[1], Sebastian Schoppmann[2], Luigi Bonavina[3], Nicholas Boyle[4], Beat Müller-Stich[5], Christian Gutschow[1]

[1]*Universitäts Spital Zürich, Klinik für Viszeral- und Transplantationschirurgie, Zürich, Schweiz*, [2]*Medizinische Universität Wien, Wien,*, [3]*IRCCS San Donato Polyclinic, San Donato Milanese, Italy*, [4]*RefluxUK, United Kingdom*, [5]*Universitätsklinikum Heidelberg, Heidelberg, Deutschland*

**Background:**
There is considerable controversy regarding optimal management of patients with paraesophageal hiatus hernia (pHH). This survey aims at identifying recommended strategies for work-up, surgical therapy, and postoperative follow-up using Delphi methodology.

**Materials and methods:**
We conducted a 2-round, 33-question, web-based Delphi survey on perioperative management (preoperative work-up, surgical procedure and follow-up) of non-revisional, elective pHH among European surgeons with expertise in upper GI. Responses were graded on a 5-point Likert scale and analyzed using descriptive statistics. Items from the questionnaire were defined as “recommended” or “discouraged” if positive or negative concordance among participants was >75%. Items with lower concordance levels were labelled “acceptable” (neither recommended nor discouraged).

**Results:**
Seventy-two surgeons with a median (IQR) experience of 23 (14-30) years from 17 European countries participated (response rate 60%). The annual median (IQR) individual and institutional caseload was 25 (15-36) and 40 (28-60) pHH-surgeries, respectively. After Delphi round 2, “recommended” strategies were defined for preoperative work-up (endoscopy), indication for surgery (typical symptoms and/or chronic anemia), surgical dissection (hernia sac dissection and resection, preservation of the vagal nerves, crural fascia and pleura, resection of retrocardial lipoma) and reconstruction (posterior crurorrhaphy with single stitches, lower esophageal sphincter augmentation (Nissen or Toupet), and postoperative follow-up (contrast radiography). In addition, we identified “discouraged” strategies for preoperative work-up (endosonography), and surgical reconstruction (crurorrhaphy with running sutures, tension-free hiatus repair with mesh only). In contrast, many items from the questionnaire including most details of mesh augmentation (indication, material, shape, placement, and fixation technique) were “acceptable”.

**Conclusion:**
This multinational European Delphi survey represents the first expert-led process to identify recommended strategies for the management of pHH. Our work may be useful in clinical practice to guide the diagnostic process, increase procedural consistency and standardization, and to foster collaborative research.

**Effects of endoluminal vacuum therapy on the perfusion of gastric conduit in a porcine model for esophagectomy**
ID: 222

**Kategorie: DGAV - Ösophagus- und Magenchirurgie**

Eleni Felinska[1], Michale Allers[1], Zi-Wei Chen[1], Alexander Studier-Fischer[1], Estelle Willuth[1], Philipp Anthony Wise[1], Beat Müller-Stich[1], Felix Nickel[1]

[1]*Klinik für Allgemein-, Viszeral- und Transplantationschirurgie, Universitätsklinikum Heidelberg , Heidelberg, Deutschland*

**Background:**
After esophagectomy, the postoperative rate of anastomotic leakage is between 5 and 30% and is the main driver of morbidity and mortality. Complication management is often based on endoluminal vacuum sponge therapy (EndoVAC) with good success rates. Vacuum therapy has been shown to improve tissue perfusion in superficial wounds but this has not been shown for gastric conduits. The present study aimed to assess changes in gastric conduit perfusion in a porcine model for esophagectomy.

**Materials and methods:**
Porcine model (n=18) underwent gastric conduit formation and induction of conduit ischemia. In three experimental groups EndoVAC therapy was used in the gastric conduit (-40 mmHg, -125 mmHg and -200 mmHg) with continuous suction. The changes in tissue perfusion and edema were assessed using a hyperspectral imaging system (HSI).

**Results:**
After EndoVAC treatment with -125 mmHg a significant and stable increase in the oxygenation index from 44.9±7.6% to 61.9±5.5% and 62.9±9.4%. (p=0.02) was seen after 60 minutes and 120 minutes respectively. A similar trend was seen under the EndoVAC treatment with -200 mmHg. There was a non-significant increase in tissue oxygenation under treatment with -40 mmHg. A significant increase of tissue water content from 40.8±7.4 to 59.5±10.3 (p=0.004) was observed after 60 minutes of EndoVAC therapy with -200 mmHg. There were no significant differences in TWI under therapy with -40 mmHg and -125 mmHg.

**Conclusion:**
EndoVAC treatment with a pressure of at least -125 mmHg significantly increases perfusion of ischemic gastric conduit. With better understanding of underlying physiology, the optimal use of EndoVac treatment can be guided and determined including a possible preemptive use for gastric conduits with impaired arterial perfusion or venous congestion. This can potentially be translated into clinical practice.

**Super-charged jejunal and colonal interposition – a promising surgical treatment option for long segment esophageal defects**
ID: 294

**Kategorie: DGAV - Ösophagus- und Magenchirurgie**

Friederike Martin[1], Jonas Raakow[1], Max Magnus Maurer[1], Beate Rau[1], Andreas Brandl[1], Eva Maria Dobrindt[1], Matthias Biebl[2], Johann Pratschke[1], Christian Denecke[1]

[1]*Charité - Universitätsmedizin Berlin, Chirurgische Klinik CCM/CVK, Berlin, Deutschland*, [2]*Abteilung für Chirurgie, Ordensklinikum Linz , Linz, Österreich*

**Background:**
Patients with long segment esophageal defects without a viable stomach conduit due to injury, surgical complications or extensive malignancy, represent a surgical challenge. In the anglo-american countries, as well as in Japan, super-charged pedicled jejunal or colonal interposition is a well-established treatment option for those patients. Here we report our single center experience with supercharged jejunal and colonal interposition.

**Materials and methods:**
In 2021 two patients with long segment esophageal defects and without viable stomach conduit, underwent super-charged jejunal or colonal interposition in our clinic. We herein describe the cases of a 56-year old woman who underwent super-charged jejunal interposition following esophageal injury caused by alkali burn and a 67-year old woman with complicated postoperative course after repeated fundoplication, who received a super-charged ileo-colic interposition. Both patients had multiple previous abdominal operations including esophagectomy and gastrectomy and presented with cervical fistulas.

**Results:**
In the case of the 67-year old patient after fundoplication, a long segment esophageal reconstruction was performed using the right hemicolon as interposition. Right hemicolon and terminal ileum were mobilized and ileocolic vessels were dissected while the Drummond arcade was preserved. The interposition graft was placed in retrosternal position and revascularisation was achieved by microvascular end-to-end anastomosis of the ileocolic vessels with the left internal mammary vessels via an access in the third intercostal space. Gastrointestinal continuity was established by side-to-side esophagoileostomy and ceocojejunostomy. Regarding the 56-year old patient after alkali burn, cervical esophageal reconstruction was performed using a jejunal interposition. After uplifting in a retrosternal position, the pedicle of the first jejunal vessel arcade was anastomosed end-to-end with the left internal mammary vessels in the third intercostal space, while the lower two thirds of the jejunal interposition graft had preserved abdominal blood flow. In both cases, proximal anastomoses healed without ischemic complications and restoration of intestinal continuity could be safely achieved.

**Conclusion:**
We recommend the consideration of supercharged jejunal or colonic interposition as a promising surgical treatment option for patients with long segment esophageal defects without viable stomach conduit. In particular, the microvascular reconstruction, performed by a plastic surgeon, provided optimal blood supply in all segments of the graft.

### Systematic Review and Meta-Analysis comparing Proximal Gastrectomy with Double-Tract-Reconstruction and Total Gastrectomy in Gastric and Gastro-Esophageal Junction Cancer Patients – still no sufficient evidence for clinical decision-making (PROSPERO registration number: CRD42021291500)

ID: 302

**Kategorie: DGAV - Ösophagus- und Magenchirurgie**

Julian Hipp[1], Hans-Christian Hillebrecht[1], Eva Kalkum[2], Rosa Klotz[2], Jasmina Kuvendjiska[1], Verena Martini[1], Stefan Fichtner-Feigl[1], Markus Diener[1]

[1]*Klinik für Allgemein- und Viszeralchirurgie, Uniklinik Freiburg, Freiburg, Deutschland*,
[2]*Studienzentrum der Deutschen Gesellschaft für Chirurgie, Uniklinik Heidelberg, Heidelberg, Deutschland*

**Background:**
To compare proximal gastrectomy with double-tract-reconstruction (PG-DTR) and total gastrectomy (TG) in patients with gastro-esophageal junction (AEG II-III) and gastric cancer.

**Materials and methods:**
We conducted systematic searches in Medline, Web of Science, and Cochrane Library until December 20, 2021. Risk of bias (RoB) was assessed using the Revised Cochrane RoB-tool and the ROBINS-I-tool, as applicable. Evidence was rated by the GRADE-approach.

**Results:**
One randomized-controlled trial (RCT), and 13 non-RCTs with 1317 patients (715 patients with TG and 602 patients with PG-DTR) were included. Patients treated by TG had a significantly higher proportion of advanced cancer stages UICC IB-III (Odds Ratio (OR): 0.68, 95%-CI: 0.51 to 0.91, p=0.01). This heterogeneity biases the observed improved overall survival of patients following PG-DTR (OR: 0.67, 95%-CI: 0.44 to 1.01, p=0.05). Both procedures were comparably efficient regarding perioperative parameters. Postoperative/Preoperative Bodyweight Ratio (Mean Difference (MD): 3.56, 95%-CI: 1.32 to 5.79, p=0.002), Postoperative/Preoperative Serum-Hemoglobin Ratio (MD 3.73, 95%-CI: 1.59 to 5.88, p<0.001) and postoperative Serum-Vitamin-B12 levels (MD 42.46, 95%-CI: 6.37 to 78.55, p=0.02) were superior after PG-DTR, while Postoperative/Preoperative Serum-Albumin Ratio (MD 1.24, 95%-CI: -4.76 to 7.24, p=0.69) and Postoperative/Preoperative-Serum-Total-Protein-Ratio (MD 1.12, 95%-CI: -2.77 to 5.00, p=0.57) were not different. Health-related Quality-of-Life-data were only reported in two studies, which found no significant advantages for PG-DTR.

**Conclusion:**
PG-DTR offers advantages in postoperative nutritional parameters compared to TG (GRADE: Moderate quality of evidence). Oncological effectiveness of PG-DTR cannot be assessed (GRADE: Very low quality of evidence). Further thoroughly-planned RCTs in Western patient cohorts are necessary to improve treatment for gastric cancer patients.

**Pathological complete response in multimodal treatment of esophageal cancer – a retrospective cohort study**
ID: 304

**Kategorie: DGAV - Ösophagus- und Magenchirurgie**

Julian Hipp[1], Jasmina Kuvendjiska[1], Hans-Christian Hillebrecht[1], Stefan Fichtner-Feigl[1], Jens Höppner[2], Markus Diener[1]

[1]*Klinik für Allgemein- und Viszeralchirurgie, Uniklinik Freiburg, Freiburg, Deutschland*, [2]*Klinik für Chirurgie – Allgemein-, Viszeral-, Thorax-, Gefäß- und Transplantationschirurgie, Universitätsklinikum Schleswig-Holstein, Campus Lübeck, Lübeck, Deutschland*

**Background:**
To evaluate pathological complete response (pCR, ypT0ypN0) after neoadjuvant treatment compared to non-complete response (non-CR) in patients with esophageal cancer (EC).

**Materials and methods:**
393 patients were retrospectively analyzed. Survival probability was analyzed in patients with: 1) pCR vs non-CR; 2) complete response of the primary tumor but persisting lymphatic metastases (non-CR-T0N+); 3) pCR and tumor-free lymphnodes exhibiting signs of postneoadjuvant regression vs. no signs of regression.

**Results:**
1) Median overall survival (mOS) was favorable in patients with pCR (pCR: mOS not reached vs. non-CR: 41 months, p<0.001). Multivariate analysis revealed, that grade of regression was not an independent predictor for prolonged survival. Instead, the achieved postneoadjuvant TNM-stage (T-stage: Hazard ratio (HR) ypT3-T4 vs ypT0-T2: 1.837; N-stage: HR ypN1-N3 vs ypN0: 2.046; Postneoadjuvant M-stage: HR ypM1 vs ycM0: 2.709), the residual tumor (R)-classification (HR R1 vs R0: 4.195) and the histologic subtype of EC (HR ESCC vs EAC: 1.688) were prognostic factors. Patients with non-CR-T0N+ have a devastating prognosis, similar to those with local non-CR and lymphatic metastases (non-CR-T+N+) (non-CR-T0N+: 22.0 months, non-CR-T+N-: mOS not reached, non-CR-T+N+: 23.0 months; p-values: non-CR-T0N+ vs. non-CR-T+N-: 0.016; non-CR-T0N+ vs. non-CR-T+N+: 0.956; non-CR-T+N- vs. non-CR-T+N+: <0.001). Regressive changes in lymphnodes after neoadjuvant treatment did not influence survival-probability in patients with pCR (mOS not reached in each group; EAC-patients: p=0.0919; ESCC-patients: p=0.828).

**Conclusion:**
Particularly the achieved postneoadjuvant ypTNM-stage influences the survival probability of patients with EC. Patients with non-CR-T0N+ have a dismal prognosis, and only true pathological complete response with ypT0ypN0 offers superior survival probabilities.

**Postoperative hiatal hernia after Ivor-Lewis esophagectomy- a growing problem in the age of minimally-invasive surgery**
ID: 307

**Kategorie: DGAV - Ösophagus- und Magenchirurgie**

Jasmina Kuvendjiska[1], Julian Hipp[1], Mira Fink[1], Jens Höppner[2], Markus Diener[1], Stefan Fichtner-Feigl[1]

[1]*UNIVERSITÄTSKLINIKUM FREIBURG, Department Chirurgie, Klinik für Allgemein und Viszeralchirurgie , Freiburg im Breisgau, Deutschland*, [2]*University Medical Center Schleswig-Holstein, Lübeck, Germany, Department of Surgery*

**Background:**
Esophageal carcinoma is the eighth most common cancer in the world and the sixth most common cause of cancer death worldwide [1]. Although multimodal treatment protocols are progressively applied, surgery remains the central part of curative treatment for most patients. Different techniques of esophagectomy with variations in terms of resection and reconstruction are applied internationally. The two-field (abdominal and thoracic) procedure is the technique most used in the western world today. In the majority of patients, the stomach is used for esophageal replacement and less commonly the colon or jejunum. Postoperative morbidity has been reduced by the use of minimally-invasive techniques, regardless of the respective technique, e.g. complete minimally-invasive or hybrid technique (HMIE) [2–4]. The main benefit of minimally-invasive esophagectomy is a reduction of pulmonary morbidity [2,3]. However, with the consecutive clinical implementation of HMIE as the standard technique, an increased occurrence of postoperative paraconduit hiatal hernia (PHH) has been observed. Although most PHH are asymptomatic, there is a risk of ischemia of the herniated intestine, with potentially fatal consequences [5]. Evidence for the comparison of HMIE versus OE with special respect to PHH occurrence is still limited.

**Materials and methods:**
A retrospective single-centre analysis was done on patients who underwent Ivor Lewis esophagectomy between 01/2009 and 04/2018 at the University Medical Center, Freiburg. Computed tomography scans and patient files were reviewed to identify the presence and characteristics of PHH.

**Results:**
306 (152 HMIE; 154 OE) patients were included. 23 patients (8%) developed PHH. In every case, the transverse colon was the herniated organ, with additional herniation of small intestine in three cases. Most frequently the hiatal hernia occurred on the left thoracic side (n= 18, 78.3%) The majority of patients (13/23) were asymptomatic at the time of diagnosis and only 4 patients (17 %) presented in an emergency setting with incarceration. The most common symptom was abdominal pain and discomfort. The rate of PHH was significantly higher after HMIE compared to OE (13.8 % vs 1.3%, p <0.001). Median time to diagnosis after HMIE was 14 months and 75.5 months after OE. No other risk factors for the development of PHH were identified in uni- and multivariate analysis. Surgical repair of PHH was done in 19/23 patients (83%). Recurrence rate of PHH after surgical repair was 6/19 (32%).

**Conclusion:**
Minimally-invasive esophagectomy represents a relevant risk factor for the development of PHH. Although most patients are asymptomatic, surgical repair is recommended to avoid incarceration with potentially fatal outcome. This new risk factor for postoperative complications must raise awareness in postoperative follow-up. Furthermore, innovative techniques for the prevention as well as repair of PHH are urgently needed especcially since there is no standard treatment for PHH

1. Ferlay, J.; Shin, H.-R.; Bray, F.; Forman, D.; Mathers, C.; Parkin, D.M. Estimates of worldwide burden of cancer in 2008: GLOBOCAN 2008. International journal of cancer 2010, 127, 2893–2917.
2. Biere, S.S.A.Y.; van Berge Henegouwen, M.I.; Maas, K.W.; Bonavina, L.; Rosman, C.; Garcia, J.R.; Gisbertz, S.S.; Klinkenbijl, J.H.G.; Hollmann, M.W.; Lange, E.S.M. de; et al. Minimally invasive versus open oesophagectomy for patients with oesophageal cancer: A multicentre, open-label, randomised controlled trial. Lancet (London, England) 2012, 379, 1887–1892.
3. Mariette, C.; Markar, S.R.; Dabakuyo-Yonli, T.S.; Meunier, B.; Pezet, D.; Collet, D.; D'Journo, X.B.; Brigand, C.; Perniceni, T.; Carrère, N.; et al. Hybrid Minimally Invasive Esophagectomy for Esophageal Cancer. The New England journal of medicine 2019, 380, 152–162.
4. Ben-David, K.; Sarosi, G.A.; Cendan, J.C.; Howard, D.; Rossidis, G.; Hochwald, S.N. Decreasing morbidity and mortality in 100 consecutive minimally invasive esophagectomies. Surgical endoscopy 2012, 26, 162–167.
5. Lubbers, M.; Kouwenhoven, E.A.; Smit, J.K.; van Det, M.J. Hiatal Hernia with Acute Obstructive Symptoms After Minimally Invasive Oesophagectomy. Journal of gastrointestinal surgery: official journal of the Society for Surgery of the Alimentary Tract 2021, 25, 603–608.

**Modified postoperative care after esophagectomy to reduce morbidity and accelerate recovery**
ID: 441

**Kategorie: DGAV - Ösophagus- und Magenchirurgie**

Christian Hillebrecht[1], Julian Hipp[1], Jasmina Kuvendjiska[1], Stefan Fichtner-Feigl[1], Markus Diener[1]

[1]*Klinik für Allgemein- und Viszeralchirurgie, Uniklinik Freiburg, Freiburg im Breisgau, Deutschland*

**Background:**
Esophageal cancers are among the most common forms of cancer and one of the leading causes of cancer-related mortality, affecting almost half a million people annually worldwide[1, 2]. Survival from EC – even with modern multimodal treatment – is poor and surgery remains a mainstay of treatment for potentially resectable esophageal cancers in the absence of distant metastasis[3]. Even though great advances have been made in surgical and postoperative treatment after esophagectomy, it remains a highly complicative procedure even at high-volume centers. Overall complication rates reach as high as 56-59% with pulmonary complications, anastomotic leakage and atrial arrhythmias being the most frequent causes of 30-day morbidity[4-6]. Postoperative morbidity is associated with increased risk of mortality and reduced oncological survival[7-9], decreases health-related quality of life for patients[10, 11], increases healthcare cost[12-14] and impedes further oncological treatment which again results in further impaired prognosis. Multifactorial perioperative management programs aiming to reduce risk factors and improve outcomes have been adopted in the form of fast-track or "enhanced recovery after surgery"-schemes[15] for colorectal surgery and have been shown to reduce perioperative morbidity. For esophageal resections however, adoption of such programs is less common and international recommendations are mostly grounded on extrapolatory evidence from similar regimens after other gastrointestinal operations. Extrapolated evidence[16-18] has led to the empirical adoption of a multifactorial post-esophagectomy routine comprising enteral decontamination (ED), application of hydroxyethyl starch solution (HES), enforced near-zero fluid balance and consequent nasogastric decompression with nil per os for the immediate postoperative course. Few clinical data exist for application of HES in elective surgical patients, only extrapolated evidence for oral decontamination after esophagectomy with gastric conduit reconstruction exists[19] and consequently leaving nasogastric tubes and adhering to nil per os until after exclusion of anastomotic leakage on postoperative day 5 is in direct contrast to international society guidelines[15].
Regarding the scarcity and inhomogeneity of data for postoperative care after esophagectomy, it was the aim of this study to describe a single-center experience at a high-volume center cohort of esophageal resections and evaluate safety and efficacy of combination of measures to reduce the severe impact of postoperative morbidity after esophagectomy.

**Materials and methods:**
Patient-, disease-, surgery-specific data as well as postoperative course and morbidity data from consecutive patients undergoing esophagectomy for cancer at a high-volume tertiary referral center between 01/2014 and 01/2021 were collected and retrospectively analyzed in our database (DRKS00024369). The Medical Ethics Committee of the University of Freiburg approved the study (21-1093 and 21-1713).
Descriptive statistics and Multivariate analyses were performed by appropriate regression models with treatment specifics and baseline values as independent variables. Statistical analysis was performed using SPSS Version 28.0.1.0 (IBM Corp., Armonk, New York, USA) and R version 4.0.0 (R Foundation for Statistical Computing, Vienna, Austria) with R-Studio (R Studio Inc., Boston, MA, USA). Differences were considered statistically significant for $p<0.05$.
The primary hypothesis was that an modified care (MC) consisting of enteral decontamination (ED), application of hydroxyethyl starch solution (HES), enforced near-zero fluid balance and consequent nasogastric decompression with nil per os for the immediate postoperative course would be associated with reduced morbidity.

**Results:**
325 patients were included in this study. 46 (14%) had received standard postoperative care (SC), whereas 279 (86%) received modified care (MC; with ED, HES, enforced near-zero fluid balance, nasogastric decompression and nil per os).
Patient and disease characteristics were not statistically significant between groups. Operation time was significantly prolonged in EC group (340.4min vs 307.2min; mean difference -33.2min, 95CI: -53.1 — -13.4). Clavien-Dindo>/=3 complications occurred significantly less frequent in EC group (58.7% vs 39.2%; mean difference -19.5%; 95CI: -34.8 — -4.1), comprehensive complication index reflected this as well (40.3 vs 29.02; mean difference -11.3; 95CI: -20.1 — -2.5). Anastomotic leakages were significantly reduced in EC group (13% vs 3.2%; mean difference -9.8, 95CI: -16.3 — -3.2), pulmonary complications, cardiac arrhythmias, delayed gastric emptying and wound infections were not significantly different between groups. Interestingly, application of HAES in EC group did not increase rates of akute kidney injury, no patient in either group required renal replacement therapy. Diuretics use did not differ significantly between groups, but average fluid balance on postoperative day 4 was significantly reduced in EC group (1733ml vs 751ml; mean difference -981.7, 95CI: -1908.7 — -54.7). Length of ICU stay and ICU readmission rates were significantly reduced (ICU stay: 10.6d vs 8.8d; mean difference -1.76, 95CI: -4.8 — -1.3; readmissions: 17.4% vs 8.6%; mean difference -8.8, 95CI: -18.1 — -1) and hospital stay hospital was significantly shorter in EC group (22.02d vs 18.7d; mean difference -3.3, 95CI: -7.1— -0.49).

**Conclusion:**
This single-center experience of a modified standard of care after esophagectomies demonstrates safety and efficacy in reducing postoperative morbidity. The compound intervention consisting of enteral decontamination, application of hydroxyethyl starch solution, enforced near-zero fluid balance and consequent nasogastric decompression with nil per os for the immediate postoperative course can safely be applied. Severe complications were significantly reduced in MC group, length of ICU and hospital stay were reduced and significantly less ICU readmissions were required. Severely restrictive volume therapy relying on colloidal solutions and diuretics did not incur additional morbidity. This study will serve to estimate effect sizes for a planned prospective trial.

1. Ferlay J, Soerjomataram I, Dikshit R, et al. Cancer incidence and mortality worldwide: sources, methods and major patterns in GLOBOCAN 2012. *Int J Cancer*. Mar 1 2015;136(5):E359-86. doi:10.1002/ijc.29210
2. Pennathur A, Gibson MK, Jobe BA, Luketich JD. Oesophageal carcinoma. *Lancet*. Feb 2 2013;381(9864):400-12. doi:10.1016/S0140-6736(12)60643-6
3. Lordick F, Mariette C, Haustermans K, Obermannova R, Arnold D, Committee EG. Oesophageal cancer: ESMO Clinical Practice Guidelines for diagnosis, treatment and follow-up. *Ann Oncol*. Sep 2016;27(suppl 5):v50-v57. doi:10.1093/annonc/mdw329
4. Low DE, Kuppusamy MK, Alderson D, et al. Benchmarking Complications Associated with Esophagectomy. *Ann Surg*. Feb 2019;269(2):291-298. doi:10.1097/SLA.0000000000002611
5. Schmidt HM, Gisbertz SS, Moons J, et al. Defining Benchmarks for Transthoracic Esophagectomy: A Multicenter Analysis of Total Minimally Invasive Esophagectomy in Low Risk Patients. *Ann Surg*. Nov 2017;266(5):814-821. doi:10.1097/SLA.0000000000002445
6. Liesenfeld LF, Sauer P, Diener MK, et al. Prognostic value of inflammatory markers for detecting anastomotic leakage after esophageal resection. *BMC Surg*. Dec 9 2020;20(1):324. doi:10.1186/s12893-020-00995-2
7. Rutegard M, Lagergren P, Rouvelas I, Mason R, Lagergren J. Surgical complications and long-term survival after esophagectomy for cancer in a nationwide Swedish cohort study. *Eur J Surg Oncol*. Jul 2012;38(7):555-61. doi:10.1016/j.ejso.2012.02.177
8. Bundred JR, Hollis AC, Evans R, Hodson J, Whiting JL, Griffiths EA. Impact of postoperative complications on survival after oesophagectomy for oesophageal cancer. *BJS Open*. Jun 2020;4(3):405-415. doi:10.1002/bjs5.50264

9. Booka E, Takeuchi H, Suda K, et al. Meta-analysis of the impact of postoperative complications on survival after oesophagectomy for cancer. *BJS Open*. Sep 2018;2(5):276-284. doi:10.1002/bjs5.64
10. Kauppila JH, Johar A, Lagergren P. Postoperative Complications and Health-related Quality of Life 10 Years After Esophageal Cancer Surgery. *Ann Surg*. Feb 2020;271(2):311-316. doi:10.1097/SLA.0000000000002972
11. Derogar M, Orsini N, Sadr-Azodi O, Lagergren P. Influence of major postoperative complications on health-related quality of life among long-term survivors of esophageal cancer surgery. *J Clin Oncol*. May 10 2012;30(14):1615-9. doi:10.1200/JCO.2011.40.3568
12. Lofgren A, Akesson O, Johansson J, Persson J. Hospital costs and health-related quality of life from complications after esophagectomy. *Eur J Surg Oncol*. Sep 29 2020;doi:10.1016/j.ejso.2020.09.032
13. Strik C, ten Broek RP, van der Kolk M, van Goor H, Bonenkamp JJ. Health-related quality of life and hospital costs following esophageal resection: a prospective cohort study. *World J Surg Oncol*. Sep 4 2015;13:266. doi:10.1186/s12957-015-0678-3
14. Goense L, van Dijk WA, Govaert JA, van Rossum PS, Ruurda JP, van Hillegersberg R. Hospital costs of complications after esophagectomy for cancer. *Eur J Surg Oncol*. Apr 2017;43(4):696-702. doi:10.1016/j.ejso.2016.11.013
15. Low DE, Allum W, De Manzoni G, et al. Guidelines for Perioperative Care in Esophagectomy: Enhanced Recovery After Surgery (ERAS((R))) Society Recommendations. *World J Surg*. Feb 2019;43(2):299-330. doi:10.1007/s00268-018-4786-4
16. Glatz T, Kulemann B, Marjanovic G, Bregenzer S, Makowiec F, Hoeppner J. Postoperative fluid overload is a risk factor for adverse surgical outcome in patients undergoing esophagectomy for esophageal cancer: a retrospective study in 335 patients. *BMC Surg*. Jan 13 2017;17(1):6. doi:10.1186/s12893-016-0203-9
17. Marjanovic G, Villain C, Timme S, et al. Colloid vs. crystalloid infusions in gastrointestinal surgery and their different impact on the healing of intestinal anastomoses. *Int J Colorectal Dis*. Apr 2010;25(4):491-8. doi:10.1007/s00384-009-0854-4
18. Veelo DP, van Berge Henegouwen MI, Ouwehand KS, et al. Effect of goal-directed therapy on outcome after esophageal surgery: A quality improvement study. *PLoS One*. 2017;12(3):e0172806. doi:10.1371/journal.pone.0172806
19. Roos D, Dijksman LM, Tijssen JG, Gouma DJ, Gerhards MF, Oudemans-van Straaten HM. Systematic review of perioperative selective decontamination of the digestive tract in elective gastrointestinal surgery. *Br J Surg*. Nov 2013;100(12):1579-88. doi:10.1002/bjs.9254

**Endoscopic Management of Anastomotic Leakage after Esophageal Surgery**
ID: 454

**Kategorie: DGAV - Ösophagus- und Magenchirurgie**

Nader El-Sourani[1], Sorin Miftode[1], Maximilian Bockhorn[1], Alexander Arlt[2], Christian Meinhardt[2]

[1]*Universitätsklinik für Allgemein - und Viszeralchirurgie, Klinikum Oldenburg, Oldenburg, Deutschland*,
[2]*Universitätsklinik für Gastroenterologie, Klinikum Oldenburg, Oldenburg, Deutschland*

**Background:**
Anastomotic leakage after esophageal surgery remains a feared complication. During the last decade, management of this complication changed from surgical revision to a more conservative and endoscopic approach. However, the treatment remains controversial as the indications for conservative, endoscopic, and surgical approaches remain non-standardized.

**Materials and methods:**
Between 2010 and 2020, all patients who underwent Ivor Lewis esophagectomy for underlying malignancy were included in this study. The data of 28 patients diagnosed with anastomotic leak were further analyzed.

**Results:**
Among 141 patients who underwent resection, 28 (19.9%) developed an anastomotic leak, eight (28.6%) of whom died. Thirteen patients were treated with endoluminal vacuum therapy (EVT), seven patients with self-expanding metal stents (SEMS) four patients with primary surgery, one patient with a hemoclip, and three patients were treated conservatively. EVT achieved closure in 92.3% of the patients with a large defect and no EVT-related complications. SEMS therapy was successful in clinically stable patients with small defect sizes.

**Conclusion:**
EVT can be successfully applied in the treatment of anastomotic leakage in critically ill patients, while SEMS should be limited to clinically stable patients with a small defect size. Surgery is only warranted in patients with sepsis with graft necrosis

### Textbook Outcome after Gastrectomy for Gastric Cancer is associated with a higher survival rate

ID: 542

**Kategorie: DGAV - Ösophagus- und Magenchirurgie**

Candan Çetinkaya Hosgör[1], Philippa Seika[1], Matthias Biebl[1,2], Jonas Raakow[1], Andreas Brandl[1], Eva Maria Dobrindt[1], Max Magnus Maurer[1], Ramin Raul Ossami Saidy[1], Johann Pratschke[1], Christian Denecke[1]

[1]*Charité Universitätsmedizin Berlin, Chirurgische Klinik, Campus Charité Mitte* | *Campus Virchow-Klinikum, Berlin, Deutschland*, [2]*Ordensklinikum Linz, Department of Surgery, Linz, Österreich*

**Background:**
The complexity of the perioperative outcome for patients with gastric cancer is not well reflected by single quality metrics. To study the effect of surgical outcome on survival, we have evaluated the relationship between textbook outcome (TO) - a new composite parameter - and oncological outcome.

**Methods:**
All patients undergoing total gastrectomy or transhiatal extended gastrectomy for gastric adenocarcinoma and signet ring cell carcinoma with curative intent between 2017 and 2020 were included. TO was defined using the classification system proposed by the PRESTO group including negative margins; collection of >15 lymph nodes; the absence of perioperative complications (Clavien-Dindo >3); the absence of reinterventions; absence of unplanned ICU admission; length of stay < 21 days; absence of 30-day readmission and 30-day mortality. We evaluated factors affecting TO by multivariate logistic regression and analyzed the correlation between TO and long-term survival using a multivariate cox proportional-hazards model.

**Results:**
Of the 111 patients included in this study, 56 (50.5%) achieved all TO metrics. Open surgery was the only factor associated with failure to achieve TO on multivariate analysis (OR 2.674, 95% CI = 1.080-6.622; p=0.034). After a mean follow up period of 598 days (12-2010) a higher survival rate (OS) was seen in patients with a perioperative TO compared to non-TO patients (OR 4.454, 95% CI = 1.55-12.78; p=0.005). Similarly, DFS was reduced in the non-TO group (OR 2.112, 95% CI = 0.55-2.83; p=0.035).

**Conclusions:**
Achieving TO is strongly associated with improved medium-term survival in gastric cancer patients and merits further focus in surgical quality improvement efforts. Of note, open surgery is a significant risk factor for failure of TO, thus underlining the importance of implementing laparoscopic gastrectomy as the standard procedure in Germany.

**Signet ring cell carcinoma is an independent risk factor for Pathologic Upstaging in Patients with T1 and T2- Gastroesophageal Adenocarcinoma**
ID: 604

**Kategorie: DGAV - Ösophagus- und Magenchirurgie**

Philippa Seika[1], Jonas Raakow[1], Andreas Brandl[1], Eva Maria Dobrindt[1], Max Magnus Maurer[1], Ramin Raul Ossami Saidy[1], Matthias Biebl[2], Johann Pratschke[1], Christian Denecke[1]

[1]*Charité Universitätsmedizin Berlin, Chirurgische Klinik, Berlin, Deutschland*, [2]*Ordensklinikum Linz, Department of Surgery, Linz, Österreich*

**Background:**
Selection of an appropriate multimodal therapy is dependent on preoperative staging in patients with gastroesophageal adenocarcinoma. Inaccuracy of staging may result in upstaging of the tumor or nodal status; whereby the extent of the tumor or nodal metastasis is underestimated. Neoadjuvant therapy remains controversial in patients with uT2 staged cancer as underestimating the tumor stage may have important therapeutical consequences. Preoperative patient and tumor characteristics may be predictive of the likelihood of pathological upstaging. We aim to determine pre-operative factors associated with pathological upstaging in T2 Gastroesophageal Adenocarcinoma.

**Materials and methods:**
All patients with a uT2 N0 stage gastroesophageal adenocarcinoma who received gastroesophageal resection without prior chemotherapy at our tertiary referral center were included in our analysis. In patients with a higher pTN compared to cTN stage upstaging was considered to have occurred. Univariate analysis and multivariable logistic regression were performed. Variables included age, sex, tumor size, histology, grade and tumor location.

**Results:**
Between 2006 and 2020, 63 patients with a T2N0 gastroesophageal adenocarcinoma were operated. Upon definitive pathological examination, 37 (27.8%) patients were upstaged beyond T2. On univariate analysis, significant predictors of upstaging include diabetes (n=5(21.42%) vs. n=5(35.71%), p=0.021), a history of heart failure (n= 2(4.08 %) vs. n=3 (21.42%), p=0.034) and signet ring cell carcinoma (n= 2(4.25 %) vs. n=5(41.67%), p=0.002). On multivariable logistic regression analysis, a significant predictor of upstaging was the presence of a signet cell carcinoma (p=<0.001). Interestingly, preoperative histological grading (G1-G3) or tumor localization were not associated with the risk of preoperative upstaging.

**Conclusion:**
A significant population of patients with a T2N0 carcinoma are understaged prior to primary operation. Importantly, the presence of a signet cell carcinoma is an independent risk factor for postoperative upstaging. Upon multidisciplinary tumor board (MDTB) discussion, preoperative therapy should be considered liberally in T2N0 signet cell gastric cancer patients.

**The evolution of management of anastomotic leaks following esophagectomy – a 10-year experience from a tertiary referral center.**
ID: 618

**Kategorie: DGAV - Ösophagus- und Magenchirurgie**

Philippa Seika[1], Jonas Raakow[1], Andreas Brandl[1], Eva Maria Dobrindt[1], Max Magnus Maurer[1], Ramin Raul Ossami Saidy[1], Matthias Biebl[2], Johann Pratschke[1], Christian Denecke[1]

[1]*Charité Universitätsmedizin Berlin, Chirurgische Klinik, Berlin, Deutschland*, [2]*Ordensklinikum Linz, Department of Surgery, Linz, Österreich*

**Background:**
Esophagectomy is the primary therapy for esophageal cancer in both early and advanced locoregional stages. It is a complex procedure with a non-negligeable rate of complications, particularly a significant rate of anastomotic leakage. There is currently no internationally accepted algorithm for the management of AL while many new innovations have been adopted in recent years. The current study covers the 10-year experience of a single institution in managing esophageal leaks.

**Materials and methods:**
Between 2010 and 2020, all patients receiving esophagectomy with gastric reconstruction and intrathoracic or cervical anastomosis at our institution were evaluated. Preoperative patient characteristics, histopathological characteristics were evaluated as well as perioperative and long-term outcome. A high thoracic anastomosis was defined as above the azygos vein. Primary outcomes were perioperative morbidity and mortality while secondary outcomes included length of stay (LOS) as well as overall- and disease-free survival. The cohort was divided into two different time periods: period A (2010-2015); period B (2016–2020).

**Results:**
Among 327 patients, 54 (16.5%) experienced an anastomotic leak. Therapy involved endosponge in 24 patients, (44.4%) stent in 38 patients (70.3%) and surgical revision in 13 patients (24.0%). Cervical anastomosis (p=<0.001), high thoracic anastomosis (p=<0.001) and no systemic inflammation (p=0.010) were predictors of successful endosponge therapy while no systemic inflammation (p=0.046) and shorter time to intervention (p=0.035) were predictors of successful stent therapy. Time to intervention (p=0.041), preoperative anaemia (p=0.030) and ASA score (p=0.012) were predictors of perioperative mortality. Failure to rescue occurred at a higher rate in period A (n=8, 5.5% vs. n=2, 1.1%; p=0.031). The rate of surgical revision and stent were significantly higher in period A while the rate of endosponge was significantly higher in period B.

**Conclusion:**
The introduction of endosponge therapy has changed the management of AL as it provides higher success rates and reduces failure to rescue particularly in cervical and high thoracic anastomoses. The necessity for surgical revision has decreased significantly throughout the observation period and plays a minor role in current treatment algorithms. Of note, the time to treatment was a significant parameter for the success of interventional treatment. Consequently, it is important to facilitate an early diagnosis of AL and successful response in patients with a high ASA score. In this regard, failure to rescue rates significantly dropped over time, indicating advancements in AL-treatment.

**Is neoadjuvant chemotherapy followed by surgery the appropriate treatment for esophagogastric signet ring cell carcinomas? A systematic review and meta-analysis.**
ID: 670

**Kategorie: DGAV - Ösophagus- und Magenchirurgie**

Sabine Schiefer[1], Nerma Crnovrsanin[1], Eva Kalkum[2], Henrik Nienhüser[1], Ingmar F Rompen[1], Georg Martin Haag[3], Beat P. Müller[1], Rosa Klotz[1], Leila Sisic[1]

[1]*Klinik für Allgemein-, Viszeral- und Transplantationschirurgie, Universitätsklinikum Heidelberg, Heidelberg, Deutschland*, [2]*Studienzentrum der Deutschen Gesellschaft für Chirurgie, Universitätsklinikum Heidelberg, Heidelberg, Deutschland*, [3]*Nationales Zentrum für Tumorerkrankungen, Universitätsklinikum Heidelberg, Heidelberg, Deutschland*

**Background:**
The prognostic impact of neoadjuvant chemotherapy on survival and tumor response in patients with esophagogastric signet ring cell carcinoma (SRCC) is still controversial. The aim of this meta-analysis is to summarize the currently available evidence regarding this topic.

**Materials and methods:**
We performed a systematic literature search and extracted data on overall survival (OS) and tumor regression in resected esophagogastric SRCC patients with or without previous neoadjuvant chemotherapy.
Eligible randomized and non-randomized, prospective and retrospective two-arm studies published from 1991-2022 were identified by systematic literature search in MEDLINE, Web of Science and Cochrane Central Register of Controlled Trials (CENTRAL) databases on September 29th, 2022. Non-english language articles were excluded. Regarding survival analysis, we used published hazard ratios (HR) if available or determined it from other survival data or survival curves. Histopathologic response rates by type of tumor were also investigated. The study was registered in PROSPERO (CRD42022298743).
According to the search results, two separate meta-analyses were performed: meta-analysis A investigating SRCC patients treated with neoadjuvant chemotherapy followed by surgery vs. surgery only regarding OS, meta-analysis B investigating patients treated with neoadjuvant chemotherapy followed by surgery comparing SRCC vs. non-SRCC patients with regard to OS and tumor regression.
The risk of bias in randomized controlled trials (RCT) was analysed using the Cochrane Collaboration tool. For non-randomized studies the risk of bias assessment was conducted in accordance to ROBINS-I tool.

**Results:**
Ten of 559 studies (1 RCT, 9 non-RCTs) were included in this systematic review investigating 3408 patients in total. Four studies compared OS in SRCC patients treated by neoadjuvant chemotherapy + surgery vs. surgery alone (1 RCT, 3 non-RCTs), three studies compared OS and four studies compared tumor regression in SRCC vs. non-SRCC patients treated by neoadjuvant chemotherapy followed by surgery.
There was no significant survival benefit for SRCC patients treated with neoadjuvant chemotherapy + surgery compared to surgery alone (HR 1.01 – CI 0.61;1.67; p=0.98). In patients treated by neoadjuvant chemotherapy + surgery non-SRCC patients showed better survival (HR 1.45 – CI 1.21; 1.74; p<0.01) and higher histopathological tumor response rates (OR 0.4 – CI 0.29; 0.56; p<0.01) after neoadjuvant chemotherapy than SRCC patients.
The risk of bias for the included studies was low to moderate according to the tools used.

**Conclusion:**
This systematic review and meta-analysis could not show a significant survival benefit for esophagogastric signet ring cell carcinoma patients treated by neoadjuvant chemotherapy followed by surgery compared to surgery alone. However, data from randomized controlled studies is scarce so far.
Signet ring cell carcinoma patients showed significantly worse survival and histopathological response rate than non-signet ring cell carcinoma patients after neoadjuvant chemotherapy + surgery. Thus, the optimal therapeutic strategy in SRC tumors remains unclear. Further data, especially from randomized controlled studies is needed to allow more individualized treatment decisions in SRCC patients.

### Reconstruction after Esophagogastrectomy by using colon conduit and Hyperspectral Imaging (HSI)

ID: 741

**Kategorie: DGAV - Ösophagus- und Magenchirurgie**

Yusef Moulla[1], Ines Gockel[2]

[1]*Universitätsklinikum Leipzig , Klinik und Poliklinik für Viszeral-, Transplantation-, Thorax-, und Gefäßchirurgie , Leipzig, Deutschland*, [2]*Universitätsklinikum Leipzig, Klinik und Poliklinik für Viszeral-, Transplantation-, Thorax-, und Gefäßchirurgie , Leipzig, Deutschland*

**Background:**
Colon conduit is an alternative interposition, which is sometimes necessary, when the stomach cannot be used. Moreover, this operation is quite complex, as it usually requires a three-cavity incision procedure (abdomen, thorax and neck), at least three anastomoses, and the surgeon must choose (ensuring a sufficient vascular supply) the optimal side of the colonic conduit and the good-perfused anastomosis region. In this presentation, we would be able to demonstrate our experience in this kind of operations and the impact of using HSI as an image-guided Surgery.

**Materials and methods:**
The surgical technique as well as the detecting of the perfusion of the colon conduit by using HSI would be demonstrate as a video presentation, The duration of this video is about 15 min.

**Results:**
The surgical technique in eight patients, who underwent reconstruction with a long segment colon conduit after esophagectomy and detecting the perfusion by using HSI from 01/05/2018 until 01/04/2022 will be demonstrate in this video-presentation

**Conclusion:**
The reconstruction after esophagectomy by using colon conduit and HSI as an image-guided surgery is a possible safe option, when the gastric conduit cannot be used.

**Validation of three different scores for risk stratification prior to esophagectomy**
ID: 754

**Kategorie: DGAV - Ösophagus- und Magenchirurgie**

Sabine Schiefer[1], Nerma Crnovrsanin[1], Henrik Nienhüser[1], Ingmar F Rompen[1], Georg Martin Haag[2], Beat P. Müller[1], Leila Sisic[1]

[1]*Allgemein-, Viszeral- und Transplantationschirurgie, Uniklinik Heidelberg, Heidelberg, Deutschland*, [2]*Nationales Zentrum für Tumorerkrankungen, Uniklinik Heidelberg, Heidelberg, Deutschland*

**Background:**
Esophagectomy remains the mainstay in the treatment of esophageal cancer. As perioperative mortality is still high for certain patient subgroups different score systems exist to identify patients with high risk. Aim of this study was to evaluate different score systems with regard to ability of identifying high risk patients in a large single center cohort, in order to possibly reduce mortality by making individual treatment decisions.

**Materials and methods:**
We identified 714 patients from a prospective database that underwent esophagectomy at our institution from 2002-2021. For every patient, the individual risk score was calculated using the 90-Day mortality risk prediction model of the International Esodata Study Group (IESG), the 30-Day mortality Steyerberg score, and the preoperative risk scale of Fuchs et al.
The areas under the receiver operating characteristic (ROC) curves were used to estimate the diagnostic performance of the different risk scores.

**Results:**
Of 714 patients 587 patients were male (82.2%), 127 were female (17.8%). 626 (87.67%) patients underwent abdomino-thoracic esophagectomy with intrathoracic anastomosis (Ivor-Lewis procedure), 42 (5.88%) underwent abdomino-thoracic esophagectomy with cervical anastomosis, 23 (3.22%) underwent transhiatal esophagectomy, 17 patients (2.38%) underwent esophago-gastrectomy, and 6 patients (0.84%) underwent other types of resection.
According to IESG score 52.1% were very low, 26.6% low, 17.5% middle, 2.8% high, and 1% very high risk.
In our collective the overall 90d-mortality rate was 6.9%, the 30d-mortality rate was 3%, and the in-hospital mortality rate was 6.7%.
The area under the ROC curve (AUC) was 0.360 (0.283-0.437) for the IESG risk prediction model, 0.645 (0.534-0.756) for the Steyerberg Score, and 0.679 (0.602-0.756) for the Fuchs risk scale.

**Conclusion:**
Existing scoring systems provide a possibility for an individual preoperative risk stratification. However, three different preoperative risk scores could not be validated in our large single-center collective. A combination of the different scoring systems might be a way to better identify high-risk patients and monitor them more intensively in order to be able to identify and treat complications at an early stage and thus reduce perioperative mortality.

**Laparoscopic versus robotic Ivor-Lewis esophagectomy – an analysis of costs and postoperative outcomes in a single-center setting**
ID: 872

**Kategorie: DGAV - Ösophagus- und Magenchirurgie**

Sebastian Knitter[1], Max Maurer[1], Axel Winter[1], Ramin Ossami Saidy[1], Andreas Brandl[1], Eva Dobrindt[1], Jonas Raakow[1], Johann Pratschke[1], Christian Denecke[1]

[1]*Chirurgische Klinik Campus Charité Mitte Campus Virchow-Klinikum, Charité – Universitätsmedizin Berlin, Berlin, Deutschland*

**Background:**
For patients with esophageal cancer or cancer of the gastroesophageal junction (EC/GEJ) scheduled for surgery, the introduction of robotic esophagectomy (RE) has raised concerns about the added financial burden in comparison to laparoscopic esophagectomy (LE). We aimed to analyze postoperative outcomes and cost-effectiveness of both approaches.

**Materials and methods:**
We collected clinicopathological and financial data on 128 consecutive patients who underwent Ivor-Lewis esophagectomy for EC/GEJ with intrathoracic anastomosis from 2017-2021 at our department. Patients were grouped according to the technical approach in LE and RE. Baseline characteristics, postoperative outcomes and financial expenses were compared between LE and RE. A binary logistic regression model was used to identify parameters associated with increased costs.

**Results:**
LE and RE accounted for median daily costs of 1,818 € and 2,023 €, respectively ($p$ = 0.246). Median total costs were comparable between the groups (29,180 € vs. 30,510 €, $p$ = 0.460), however, costs for surgery were higher for RE (12,370 € vs. 10,059 €, $p$ < 0.001). Readmission to ICU (hazard ratio [HR] = 7.0, 95% confidence interval [CI] = 1.7-28.0, $p$ = 0.006), length of stay (HR [95% CI] = 14.5 [1.7-125.2], $p$ = 0.015), anastomotic leak (HR [95% CI] = 8.5 [1.8-40.4], $p$ = 0.007), and postoperative pneumonia (HR [95% CI] = 4.3 [1.1-17.1], $p$ = 0.036) were independently associated with increased financial expenses.

**Conclusion:**
RE may be considered a valid alternative to LE in terms of financial expenses. Still, typical postoperative cost drivers should be considered for improved perioperative management.

**Tables and Figures**
**Table 1.** Clinicopathological data of 128 patients who underwent laparoscopic or robotic Ivor-Lewis esophagectomy

| Characteristics | LE (N = 91) | RE (N = 37) | *p* |
|---|---|---|---|
| Male sex, n (%) | 75 (82) | 32 (87) | 0.573 |
| Median age at resection, years (range) | 63 (44-82) | 64 (44-81) | 0.948 |
| Age ≥ 65 years, n (%) | 38 (42) | 17 (46) | 0.664 |
| Median BMI, kg/m$^2$ (range) | 25.1 (16.1-36.4) | 25.8 (15.7-36.1) | 0.673 |
| BMI ≥ 30 kg/m$^2$, n (%) | 15 (17) | 4 (11) | 0.375 |

| | | | |
|---|---|---|---|
| Comorbidities | | | |
| Diabetes, n (%) | 12 (13) | 6 (16) | 0.655 |
| Cardiovascular disease, n (%) | 9 (10) | 4 (11) | 1 |
| Arterial hypertension, n (%) | 55 (60) | 20 (54) | 0.506 |
| Pulmonary disease, n (%) | 14 (15) | 4 (11) | 0.500 |
| Liver cirrhosis, n (%) | 11 (12) | 3 (8) | 0.756 |
| ASA physical status, n (%) | | | 0.625 |
| I | 5 (6) | 1 (3) | |
| II | 35 (39) | 15 (42) | |
| III | 46 (52) | 20 (56) | |
| IV | 3 (3) | 0 (0) | |
| Preoperative therapy, n (%) | | | **0.045** |
| None | 8 (9) | 2 (5) | |
| Chemotherapy | 50 (55) | 29 (78) | |
| Radiochemotherapy | 33 (36) | 6 (16) | |
| Tumor location, n (%) | | | 0.893 |
| Esophagus | 48 (53) | 20 (54) | |
| Gastroesophageal junction | 43 (47) | 17 (46) | |
| Median duration of resection (range), min | 372 (205-570) | 421 (305-543) | **<0.001** |
| T category, n (%) | | | 0.837 |
| T0 | 26 (29) | 11 (30) | |
| T1 | 20 (22) | 8 (22) | |
| T2 | 14 (15) | 7 (19) | |
| T3 | 28 (31) | 11 (30) | |
| T4 | 3 (3) | 0 (0) | |
| N category, n (%) | | | 0.516 |
| N0 | 58 (64) | 22 (60) | |
| N1 | 10 (11) | 6 (16) | |
| N2 | 18 (20) | 5 (14) | |
| N3 | 5 (6) | 4 (11) | |
| Median number of lymph nodes removed (range) | 32 (9-72) | 34 (22-61) | 0.177 |
| UICC stage, n (%) | | | 0.972 |
| I | 47 (53) | 19 (53) | |
| II | 9 (10) | 4 (11) | |
| III | 25 (28) | 9 (25) | |
| IV | 8 (9) | 4 (11) | |
| Lymphangiosis carcinomatosa, n (%) | 16 (17) | 7 (19) | 0.741 |
| Histologic type, n (%) | | | 0.078 |
| Adenocarcinoma | 61 (68) | 30 (83) | |

| | | | |
|---|---|---|---|
| Squamous cell carcinoma | 29 (32) | 6 (17) | |
| Tumor grading (G), n (%) | | | 0.845 |
| G1 | 3 (4) | 1 (4) | |
| G2 | 42 (58) | 16 (64) | |
| G3 | 28 (38) | 8 (32) | |
| Positive resection margins, n (%) | 5 (6) | 2 (5) | 1 |
| Median duration of ICU stay (range), days | 3 (1-67) | 4 (1-10) | 0.528 |
| Median duration of hospital stay (range), days | 17 (9-110) | 15 (8-80) | 0.205 |
| Need for intraoperative RBC transfusions, n (%) | 9 (10) | 0 (0) | 0.058 |
| Anastomotic leak, n (%) | 16 (18) | 4 (11) | 0.339 |
| Postoperative pneumonia, n (%) | 23 (25) | 3 (8) | **0.029** |
| Readmission to ICU, n (%) | 21 (23) | 7 (19) | 0.606 |
| 30-day mortality, n (%) | 0 (0) | 0 (0) | - |
| 90-day mortality, n (%) | 1 (2) | 0 (0) | 1 |

LE, laparoscopic esophagectomy; RE, robotic esophagectomy

**Table 2.** Financial data of 128 patients who underwent laparoscopic or robotic Ivor-Lewis esophagectomy

| **Parameters** | **LE (N = 91)** | **RE (N = 37)** | ***P*** |
|---|---|---|---|
| **Costs**, median, € (range) | | | |
| Surgery | 10,059 (6,589-20,170) | 12,370 (9,862-19,046) | **<0.001** |
| Anesthesia | 3,106 (0-9,816) | 3,375 (1,691-6,746) | 0.090 |
| ICU | 4,981 (696-206,750) | 4,248 (548-126,105) | 0.236 |
| Dialysis | 0 (0-29,785) | 0 (0-0) | 0.084 |
| Normal ward | 6,412 (705-41,230) | 6,708 (3,177-25,709) | 0.758 |
| Laboratory tests | 1,748 (688-12,232) | 1,684 (1,080-5,481) | 0.795 |
| Cardiology | 0 (0-3,937) | 0 (0-2,142) | 0.152 |
| Radiology | 803 (118-8,161) | 540 (117-4,671) | 0.109 |
| Endoscopy | 587 (0-14,024) | 339 (0-14,045) | 0.228 |
| Other diagnostics | 215 (0-1,078) | 212 (0-384) | 0.732 |
| Other therapeutics | 360 (0-6,282) | 355 (68-2,427) | 0.476 |
| Patient admission | 0 (0-200) | 0 (0-67) | 0.625 |
| Daily costs | 1,818 (811-3,365) | 2,023 (1,051-4,180) | 0.246 |
| Total costs | 29,180 (18,649-303,453) | 30,510 (22,256-185,871) | 0.460 |

LE, laparoscopic esophagectomy; RE, robotic esophagectomy

**Table 3.** Multivariate analysis of factors associated with increased total costs in 128 patients who underwent laparoscopic or robotic Ivor-Lewis esophagectomy

| | **UV** | | | **MV** | |
|---|---|---|---|---|---|
| **Parameters** | **<42,990 € per case (n = 96)** | **≥42,990 € per case (n = 32)** | ***P*** | **HR (95 % CI)** | ***P*** |
| Male sex, n (%) | 81 (84) | 26 (81) | 0.679 | | |
| Age ≥65 years, n (%) | 39 (41) | 16 (50) | 0.354 | | |
| BMI ≥30 kg/m$^2$, n (%) | 15 (16) | 4 (13) | 1 | | |
| ASA score ≥3, n (%) | 49 (52) | 20 (65) | 0.229 | | |
| Length of procedure, n (%) | | | | | |
| ≥386 minutes | 51 (53) | 13 (41) | 0.221 | | |
| Readmission to ICU, n (%) | 8 (8) | 20 (63) | **<0.001** | 7.0 (1.7-28.0) | **0.006** |
| Length of ICU stay, n (%) | | | | | |
| ≥4 days | 44 (46) | 21 (66) | **0.052** | | NS |
| Length of hospital stay, n (%) | | | | | |
| ≥16 days | 37 (39) | 31 (97) | **<0.001** | 14.5 (1.7-125.2) | **0.015** |
| Anastomotic leak, n (%) | 4 (4) | 16 (50) | **<0.001** | 8.5 (1.8-40.4) | **0.007** |
| Postoperative pneumonia, n (%) | 10 (10) | 16 (50) | **<0.001** | 4.3 (1.1-17.1) | **0.036** |

UV, univariate analysis; MV, multivariate analysis; BMI, body mass index; ASA, American Society of Anesthesiologists; ICU, intensive care unit; NS, not significant; n/a, not applicable

**Open versus hand-assisted laparoscopic gastrectomy for gastric cancer – an analysis of costs and postoperative outcomes in a single-center setting**
ID: 874

**Kategorie: DGAV - Ösophagus- und Magenchirurgie**

Sebastian Knitter[1], Max Maurer[1], Axel Winter[1], Ramin Ossami Saidy[1], Andreas Brandl[1], Eva Dobrindt[1], Jonas Raakow[1], Johann Pratschke[1], Christian Denecke[1]

[1]*Chirurgische Klinik Campus Charité Mitte Campus Virchow-Klinikum, Charité – Universitätsmedizin Berlin, Berlin, Deutschland*

**Background:**
Since minimal-invasive procedures have been introduced to the surgical field, concerns about the safety and increased financial expanses of hand-assisted laparoscopic gastrectomy (HALG) in comparison to the conventional open gastrectomy (OG) for patients with gastric cancer (GC) were raised. We aimed to evaluate postoperative outcomes and cost-effectiveness after OG and HALG for patients with GC.

**Materials and methods:**
Clinical and financial data on patients who underwent gastrectomy for GC from 2017 to 2020 at our institution were collected. Patients were stratified according to the surgical access in OG or HALG. The Chi square, Fisher's Exact or Kruskal-Wallis H test were used as appropriate.

**Results:**
We identified 43 patients who underwent OG (n = 10) or HALG (n = 33) for gastric cancer. Clinical baseline characteristics and postoperative outcomes after OG and HALG were comparable (see Table 1). While OG and HALG accounted for similar daily costs of 1,309 € and 1,334 €, respectively ($p$ = 0.854), total costs after HALG were slightly reduced in comparison to OG (17,320 vs. 20,897 €, $p$ = 0.402). Individual expanses were mostly comparable between the groups (see Table 2).

**Conclusion:**
HALG is a safe and cost-effective alternative to OG and should be seen as the first choice for patients with gastric cancer. Total costs may even be reduced after HALG.

### Tables and Figures

**Table 1.** Clinicopathological data of 43 patients who underwent open or hand-assisted laparoscopic gastrectomy

| Characteristics | OG (N = 10) | HALG (N = 33) | *p* |
|---|---|---|---|
| Male sex, n (%) | 5 (50) | 20 (61) | 0.717 |
| Median age at resection, years (range) | 55 (45-82) | 68 (33-89) | 0.175 |
| Age ≥ 65 years, n (%) | 4 (40) | 20 (61) | 0.295 |
| Median BMI, kg/m$^2$ (range) | 26.2 (20.7-35.4) | 23.7 (16.8-31.1) | 0.077 |
| BMI ≥ 30 kg/m$^2$, n (%) | 1 (10) | 2 (6) | 0.558 |
| Comorbidities | | | |
| Diabetes, n (%) | 1 (10) | 6 (18) | 1 |
| Cardiovascular disease, n (%) | 0 (0) | 3 (9) | 1 |
| Arterial hypertension, n (%) | 4 (40) | 20 (61) | 0.295 |
| Pulmonary disease, n (%) | 1 (10) | 3 (9) | 1 |
| Liver cirrhosis, n (%) | 1 (10) | 2 (6) | 0.558 |
| ASA physical status, n (%) | | | 0.587 |
| I | 1 (10) | 1 (3) | |
| II | 2 (20) | 12 (36) | |
| III | 7 (70) | 19 (58) | |
| IV | 0 (0) | 1 (3) | |
| Preoperative chemotherapy, n (%) | | | 0.253 |
| FLO | 1 (10) | 3 (10) | |
| FLOT | 7 (70) | 27 (87) | |
| Combination or other agents | 1 (10) | 0 (0) | |
| None | 1 (10) | 1 (3) | |
| Type of gastrectomy, n (%) | | | 1 |
| Total | 10 (100) | 30 (31) | |
| Subtotal | 0 (0) | 3 (9) | |
| Median duration of resection (range), min | 285 (197-413) | 250 (183-385) | 0.524 |
| T category, n (%) | | | 0.112 |
| T0 | 2 (20) | 1 (3) | |
| T1 | 0 (0) | 9 (27) | |

| | | | |
|---|---|---|---|
| T2 | 2 (29) | 8 (24) | |
| T3 | 3 (30) | 11 (33) | |
| T4 | 3 (30) | 4 (12) | |
| N category, n (%) | | | 0.680 |
| N0 | 6 (60) | 22 (67) | |
| N1 | 1 (10) | 6 (18) | |
| N2 | 1 (10) | 1 (3) | |
| N3 | 2 (20) | 4 (12) | |
| Median number of lymph nodes removed (range) | 28.5 (13-61) | 35 (15-70) | 0.561 |
| UICC stage, n (%) | | | 0.098 |
| I | 3 (30) | 6 (18) | |
| II | 0 (0) | 13 (39) | |
| III | 1 (10) | 4 (12) | |
| IV | 6 (60) | 10 (30) | |
| Lymphangiosis carcinomatosa, n (%) | 5 (50) | 7 (21) | 0.110 |
| Lauren type, n (%) | | | 0.488 |
| Diffuse | 6 (60) | 15 (46) | |
| Intestinal | 4 (40) | 18 (55) | |
| Tumor grading (G), n (%) | | | 0.416 |
| G1 | 0 (0) | 1 (3) | |
| G2 | 1 (11) | 10 (30) | |
| G3 | 8 (89) | 22 (67) | |
| Positive resection margins, n (%) | 1 (10) | 1 (3) | 0.415 |
| Median duration of hospital stay (range), days | 14 (8-27) | 13 (7-26) | 0.470 |
| Need for RBC transfusions, n (%) | 2 (20) | 3 (9) | 0.575 |
| Postoperative morbidity (grade 1-2), n (%) | 1 (10) | 3 (9) | 1 |
| Major postoperative morbidity (grade ≥3a), n (%) | 1 (10) | 0 (0) | 0.233 |
| Anastomotic leak, n (%) | 0 (0) | 0 (0) | - |
| Postoperative pneumonia, n (%) | 0 (0) | 0 (0) | - |
| 30-day mortality, n (%) | 0 (0) | 0 (0) | - |
| 90-day mortality, n (%) | 0 (0) | 0 (0) | - |

OG, open gastrectomy; HALG, hand-assisted laparoscopic gastrectomy

**Table 2.** Financial data of 43 patients who underwent open or hand-assisted laparoscopic gastrectomy

| **Parameters** | **OG (N = 10)** | **HALG (N = 33)** | ***P*** |
|---|---|---|---|
| **Costs**, median, € (range) | | | |
| Surgery | 6,150 (3,648-10,354) | 6,774 (3,719-9,336) | 0.470 |
| Anesthesia | 2,073 (1,754-3,479) | 2,232 (906-4,611) | 0.832 |
| ICU | 1,481 (411-6,139) | 802 (118-5,242) | 0.237 |
| Dialysis | 0 (0-0) | 0 (0-0) | 1 |
| Normal ward | 4,501 (2,987-9,398) | 4,562 (2,235-9,881) | 0.989 |
| Laboratory tests | 1,962 (1,292-4,544) | 1,269 (623-2,514) | **0.001** |
| Cardiology | 0 (0-0) | 0 (0-0) | 1 |
| Radiology | 309 (43-388) | 323 (42-1,673) | 0.356 |
| Endoscopy | 240 (0-1185) | 226 (0-5,661) | 1 |
| Other diagnostics | 110 (0-260) | 82 (0-257) | 0.112 |
| Other therapeutics | 129 (57-699) | 154 (-97-415) | 0.561 |
| Patient admission | 0 (0-0) | 0 (0-136) | 0.788 |
| Daily costs | 1,309 (776-1,800) | 1,334 (849-2,075) | 0.854 |
| Total costs | 20,897 (12,330-26,137) | 17,320 (12,018-26,387) | 0.402 |

OG, open gastrectomy; HALG, hand-assisted laparoscopic gastrectomy

### Regional Transplant Rates Depend More on Physician-Dependent Variables than on Proximity to Transplant Center

ID: 626

**Kategorie: DGAV - Transplantationschirurgie**

Elnaz Payani[1], Nikolaus Börner[2], Dionysios Kolliogiannis[2], Stefan M. Brunner[3], Ingo Klein[4], Ursula Ehmer[5], Gerald Denk[6], Christian M. Lange[6], Klaudja Ograja[7], Jens Werner[8], Markus Guba[8]

[1]*LMU, Allgemein-, Viszeral- und Transplantationschirurgie, Munich, Deutschland*, [2]*Allgemein-, Viszeral- und Transplantationschirurgie, Munich, Deutschland*, [3]*Allgemein-, Viszeral- und Transplantationschirurgie Regensburg, Regensburg, Deutschland*, [4]*Allgemein-, Viszeral- und Transplantationschirurgie, Würzburg, Deutschland*, [5]*Gastroenterologie Rechts der Isar, München, Deutschland*, [6]*Gastroenterologie LMU, München, Deutschland*, [7]*Transplantzentrum LMU, München, Deutschland*, [8]*Allgemein-, Viszeral- und Transplantationschirurgie LMU, München, Deutschland*

**Background:**
Several reports from the United States (US) and United Kingdom (UK) describe unequal access to liver transplantation at multiple levels, in waiting list admission, delisting practices, and donor organ allocation. Since the health care and social security systems in the US and UK are not comparable with those in Germany, the results from these countries cannot be applied without restriction to Germany.
The objective of this work was to uncover inequalities in access to liver transplantation in Bavaria, Germany.

**Materials and methods:**
For this purpose, the annual transplantation rate per 1 million inhabitants for the respective districts was determined from the aggregated postal codes of the place of residence of transplanted patients. The variables examined were proximity and travel time to the nearest transplant center, as well as the care category of the regional hospital. In addition, we assessed whether the head of gastroenterology at the regional hospital through which liver transplant candidates are referred was trained at a liver transplant center.

**Results:**
We could not demonstrate a direct relationship between proximity or travel time to the nearest transplant center and access to liver transplantation. Multivariate regression analysis show that liver transplant training ($p < 0.0001$) of the chief physician (gastroenterologist) of the regional hospital was the most decisive independent factor for access to liver transplantation within a district.

**Conclusion:**
We show that the transplant training experience of the head of gastroenterology at a regional hospital is an independent factor for the regional transplantation rate. Therefore, it appears important to maintain some liver transplant expertise outside the transplant centers in order to properly identify and assign potential transplant candidates for transplantation.

**Transplantation of Liver Grafts from elderly donors results in reduced Long-term Graft- and Patient Survival: A nationwide Registry Analysis in Germany**
ID: 793

**Kategorie: DGAV - Transplantationschirurgie**

Simon Moosburner[1,2], Karl Hillebrandt[1,2], Joseph Gaßner[1,2], Georg Lurje[1], Zoltan Czigany[1], Brigitta Globke[1,2], Robert Öllinger[1], Wenzel Schöning[1], Felix Braun[3], Johann Pratschke[1], Igor Sauer[1], Nathanael Raschzok[1,2]

[1]*Charité – Universitätsmedizin Berlin, corporate member of Freie Universität Berlin and Humboldt-Universität zu Berlin, Department of Surgery Campus Charité Mitte | Campus Virchow-Klinikum, Berlin, Deutschland*, [2]*Berlin Institute of Health at Charité – Universitätsmedizin Berlin, BIH Academy, Clinician Scientist Program, Berlin, Deutschland*, [3]*Department of General, Visceral, Thoracic, Transplantation and Pediatric Surgery, Campus Kiel, UKSH, Kiel, Deutschland*

**Background:**
Liver transplantation remains the treatment of choice for patients with end-stage liver disease and certain hepatic malignancies. Measures to expand the donor pool, including the acceptance of so-called extended criteria donor (ECD) grafts, are necessary to alleviate the shortage of suitable allografts for liver transplantation. ECD grafts are from elderly (>65 years) or obese donors (BMI >30), donors with elevated liver enzymes or with the presence of steatosis hepatis. Risks associated with ECD grafts include higher rates of early allograft dysfunction, primary dysfunction, and increased rates of retransplantation. However, due to the organ shortage, most grafts currently transplanted in Germany present with at least one ECD criterion. We here aimed to analyze the effects of grafts from donors >65 years on the outcome after liver transplantation on a nationwide level.

**Materials and methods:**
We analyzed the German Transplant registry, which is comprised of data from 2006 to 2016 of all liver transplantations performed in Germany and includes anonymized patient and graft survival data. Kaplan-Meier survival analysis were performed.

**Results:**
During the analysis period, 11,409 liver transplantations were performed in Germany. 95% were full size graft transplantations and 3.4% were extended right lobe transplantations. Almost a third of livers (32%) were allocated via the rescue allocation procedure of Eurotransplant, and 1,642 (14%) patients were transplanted with the high urgency status. Most recipients (64%) were male, while donors were equally male and female. Median cold ischemia time was 523 minutes. Donor age > 65 years was the most common ECD criterion (27%) in transplanted livers. When stratifying for donor age, 1- and 3-year patient survival was 76% and 69% vs. 69% and 62% in recipients of elderly donors (> 65 years), respectively ($p < 0.001$) (Figure 1). 1- and 3-year graft survival was 72% and 65% compared to 65% and 59% in patients receiving grafts from elderly donors ($p < 0.001$) (Figure 2). Donor and recipient age correlated well (Spearman $R = 0.25$, $p < 0.001$). Recipients of elderly donors had a lower media MELD score (18 vs. 21, $p < 0.001$) and were less frequently listed as high urgent (5.3% vs. 14%) at the time of transplantation. Temporal trends could not be analyzed due to the anonymization of data.

**Conclusion:**
This is the first analysis of the German Transplantation registry database to date. Initial analysis suggests an impact of donor age on long-term outcomes after liver transplantation. Confounding factors such as recipient health status and other organ quality parameters do not seem to influence this result. Further in-depth analyses are needed, to assess the effect on future liver transplantations.

**Hepatocellular Carcinoma Recurrence and its impact on survival after Liver Transplantation in the Swiss Transplant Cohort Study**
ID: 825

**Kategorie: DGAV - Transplantationschirurgie**

Severin Gloor[1], Pierre-Yves Bochud[2], Beat Müllhaupt[3], Emiliano Giostra[4], David Semela[5], Christian Garzoni[6], Andreas Cerny[7], Jean-Francois Dufour[1], Annalisa Berzigotti[1], Anja Lachenmayer[1]

[1]*Inselspital, Bern University Hospital, Department of Visceral Surgery and Medicine, Bern, Schweiz*, [2]*Lausanne University Hospital, Infectious Diseases Service, Lausanne, Schweiz*, [3]*University Hospital Zürich, Swiss Hepato-Pancreato-Biliary Center and Department of Gastroenterology and Hepatology, Zürich, Schweiz*, [4]*Geneva University Hospital, Department of Gastroenterology and Hepatology, Geneva, Schweiz*, [5]*Cantonal Hospital St.Gallen, Division of Gastroenterology, St.Gallen, Schweiz*, [6]*Clinica Luganese, Departments of Internal Medicine and Infectious Disease, Lugano, Schweiz*, [7]*Epatocentro Ticino, Lugano, Schweiz*

**Background:**
Hepatocellular carcinoma (HCC) is the second most common cause of cancer-related death and the main cause of death in patients with cirrhosis. Although liver transplantation (LT) represents a curative treatment option for both HCC and the underlying liver disease, outcomes are often hampered by tumor recurrence. Despite limited organ availability several groups have proposed to expand the inclusion criteria for LT for HCC due to acceptable post-transplant outcomes even in more advanced tumor stages. We therefore aimed to analyze the incidence of recurrence and the long-term outcomes after LT for HCC in association with known clinical risk factors in the Swiss Transplant Cohort study (STCS).

**Materials and methods:**
This study is a retrospective analysis of the prospective multicenter STCS database analyzing all patients transplanted for HCC in Switzerland from May 2008 until December 2018. The aims of this study were to assess the HCC recurrence rate after liver transplantation in Switzerland and to identify clinical and histopathological factors associated with HCC recurrence and survival, respectively.

**Results:**
Forty-seven (10.4%) of 451 transplanted patients developed HCC recurrence after LT with a mean time to recurrence of 22 months (SD ±17). While MELD score (10.2 vs. 12.6, $p = 0.132$) and ALBI score (-2.23 vs. -2.03, $p = 0.080$) were comparable between patients with (n=47) and without recurrence (n=404), alpha-fetoprotein (AFP) prior to transplantation was significantly higher (123 ng/ml vs. 42 ng/ml, $p < 0.001$) in patients developing recurrence. Patients transplanted outside the MILAN criteria developed recurrence significantly more often as when transplanted within MILAN (26 [19%] vs. 19 [8%], $p > 0.001$).
Univariate analysis for predictors of HCC recurrence showed that elevated AFP ($p = 0.001$), macrovascular invasion ($p < 0.001$), previous transarterial tumor embolization ($p < 0.001$) and microvascular invasion ($p < 0.001$) were associated with HCC recurrence, whereas solitary tumors <5 cm ($p < 0.001$) and transplantation inside of MILAN criteria ($p < 0.001$) were a negative predictor of HCC recurrence. Multivariate analysis revealed microvascular tumor invasion (hazard ratio 2.6, 95% confidence interval 1.2-5.8, $p = 0.018$) as a significant independent predictor of HCC recurrence.
The 3- and 5-year recurrence free survival rate was 89% and 85%, respectively. Patients transplanted within MILAN criteria had a 3- and 5-year recurrence free survival rate of 93% and 91%, respectively, compared with 81% and 75% when transplanted outside MILAN. The 3- and 5-year overall survival rate was 85% and 79%, respectively, for all patients and 89% and 85%, respectively, for patients transplanted within MILAN compared with 78% and 71%, respectively, for patients outside MILAN.

HCC progression was the main cause of death (n=34/36 [94%]) in patients with recurrence and the only significant predictor of death in the multivariate analysis (hazard ratio 19.1, 95% confidence interval 8.6-42.2, *p >0.001*).

**Conclusion:**
Although HCC recurrence after liver transplantation occurs in 10.4 % of all patients in Switzerland and is associated with the known predictors AFP, previous tumor embolization, macrovascular and microvascular invasion, the 5-year overall survival of 79% (85% within MILAN vs. 71% outside MILAN) remains high compared with the minimum proposed benchmark of 65% at 5 years.

### Oxygenation in rodent normothermic ex vivo liver machine perfusion

ID: 900

**Kategorie: DGAV - Transplantationschirurgie**

Linda Gilles[1], Kristina Knaub[1], Johannes Bornemann[1], Simon Moosburner[1,2], Johann Pratschke[1], Igor Maximilian Sauer[1], Nathanael Raschzok[1,2], Joseph Gassner[1,2]

[1]*Charité – Universitätsmedizin Berlin, corporate member of Freie Universität Berlin and Humboldt-Universität zu Berlin, Department of Surgery, Experimental Surgery, Berlin, Deutschland*, [2]*Berlin Institute of Health at Charité – Universitätsmedizin Berlin, BIH Academy, Clinician Scientist Program, Berlin, Deutschland*

**Background:**
Liver transplantation remains the treatment of choice for patients with end-stage liver diseases, although the availability of suitable allografts remains limited (1). Normothermic machine perfusion (NMP) has been proposed as a technique to overcome the scarcity of viable grafts in the donor pool by improving acceptance of extended criteria donors (2-4). Experimental research of small animal liver perfusion has been proven to be valuable for the establishment of NMP (5). Experimental protocols allow the investigation of optimal perfusion conditions. Under normothermic conditions, oxygenation via oxygen carriers is vital for organ function and viability. In this study we investigate the effects of varying levels of oxygenation on organ function and quality in a rat NMP system.

**Materials and methods:**
12 male Sprague-Dawley rat livers were perfused via the portal vein as well as the hepatic artery using a custom-built small animal perfusion circuit with integrated dialysis circuit for 6 hours with varying levels of oxygenation (6-8). By either reducing the surface area of the silicon membrane oxygenator or the airflow oxygen percentage, we generated conditions ranging from low to superphysiological oxygenation. In a final group, the oxygen supply was continuously adjusted to the perfusate $pO_2$ in order to maintain levels between 100 and 200mmHg.

**Results:**
Perfusion with a reduced surface area for gas exchange led to an accumulation of $CO_2$ ($pCO_2$ at T6h: 63 mmHg vs. 29 mmHg; p=0.048) and a corresponding decrease in pH (pH at T6h: 6.86 vs. 7.17; p=0.002) and even haemolysis (Hct at T6h 11.8% vs. 16.9%; p=0.001). All livers started with a good lactate clearance, which was maintained by hyper- and normoxygenated livers but for the low-oxygenated livers lactate increased at the end of perfusion (lactate at T6h: 4.579 ±0.960 mmol/l vs. 2.812 ±0.289 mmol/l; p=0.073). Bile production normalized to liver weight was significantly higher in hyperoxygenated livers than for low oxygenated livers (Bile/weight at T6h: 12.75 vs. 45.34 mg/g/h; p=0.016). The calculated oxygen uptake rate (OUR) of the graft was significantly higher in the hyperoxygenated perfusion compared to the other two groups (p=0.04; Figure 1).

**Conclusion:**
Livers perfused with reduced gas exchange showed inferior performance in our small animal NMP model compared to physiological and superphysiological oxygenation. Further research should focus on levels of reactive oxygen species in hyperoxygenated grafts as well as metabolomic changes. This will help to improve clinically used protocols for NMP.

**References:**

1. Tacke F, Kroy DC, Barreiros AP, Neumann UP. Liver transplantation in Germany. Liver Transpl. 2016;22(8):1136-42.

2. Michelotto J, Gassner J, Moosburner S, Muth V, Patel MS, Selzner M, et al. Ex vivo machine perfusion: current applications and future directions in liver transplantation. Langenbecks Arch Surg. 2021;406(1):39-54.
3. Moosburner S, Raschzok N, Schleicher C, Bosebeck D, Gassner J, Ritschl PV, et al. [Declined Liver Grafts - Analysis of the German Donor Population from 2010 to 2018]. Zeitschrift für Gastroenterologie. 2020;58(10):945-54.
4. Nasralla D, Coussios CC, Mergental H, Akhtar MZ, Butler AJ, Ceresa CDL, et al. A randomized trial of normothermic preservation in liver transplantation. Nature. 2018;557(7703):50-6.
5. Muth V, Gassner JMGV, Moosburner S, Lurje G, Michelotto J, Strobl F, et al. Ex vivo liver machine perfusion: comprehensive review of common animal models. Tissue Engineering Part B: Reviews. 2022.
6. Claussen F, Gassner J, Moosburner S, Wyrwal D, Nosser M, Tang P, et al. Dual versus single vessel normothermic ex vivo perfusion of rat liver grafts using metamizole for vasodilatation. PLoS One. 2020;15(7):e0235635.
7. Gassner J, Nosser M, Moosburner S, Horner R, Tang P, Wegener L, et al. Improvement of Normothermic Ex Vivo Machine Perfusion of Rat Liver Grafts by Dialysis and Kupffer Cell Inhibition With Glycine. Liver Transpl. 2019;25(2):275-87.
8. Nosser M, Gassner J, Moosburner S, Wyrwal D, Claussen F, Hillebrandt KH, et al. Development of a Rat Liver Machine Perfusion System for Normothermic and Subnormothermic Conditions. Tissue Eng Part A. 2020;26(1-2):57-65.

**GRAPHICS**

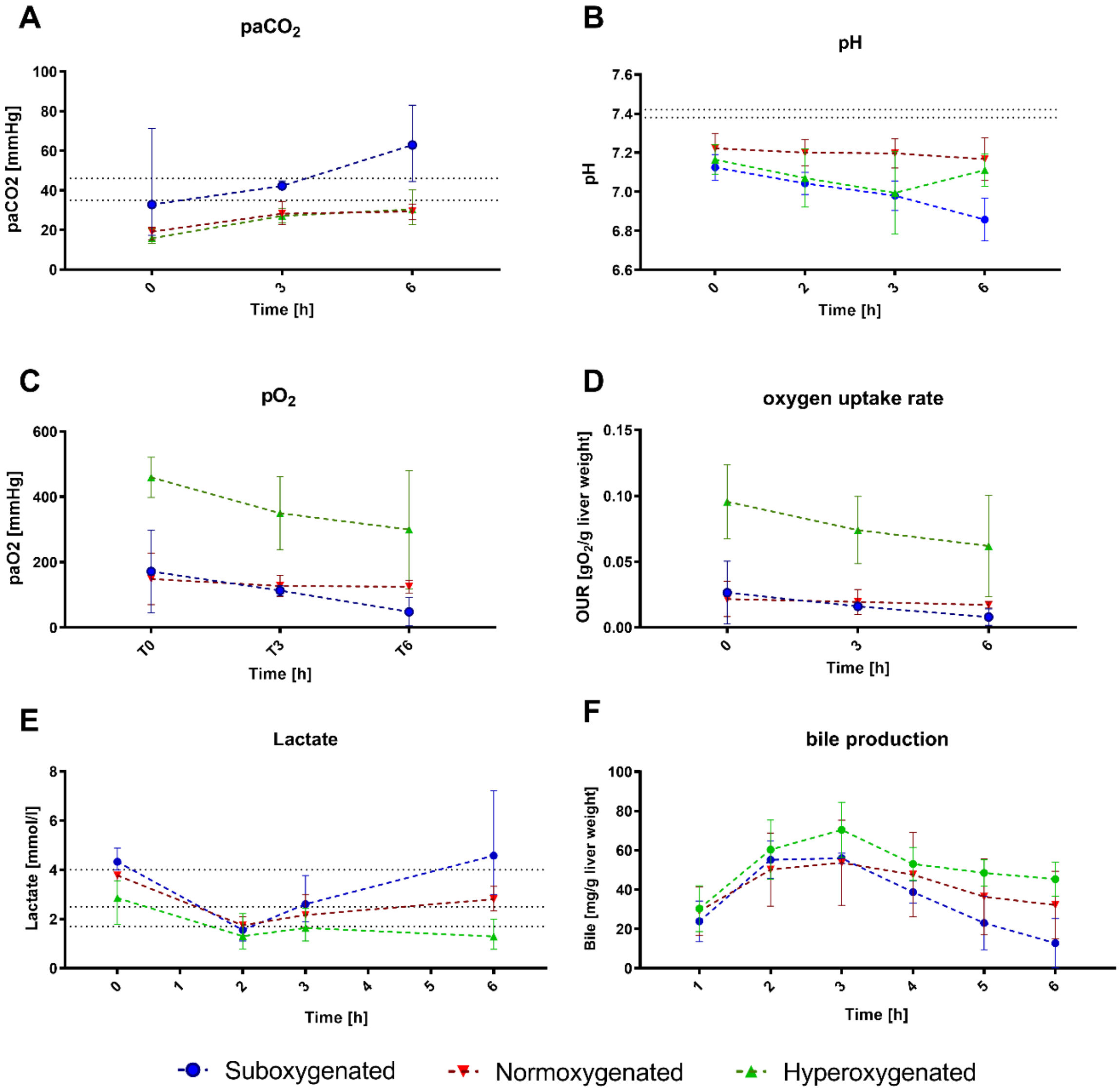

**Figure 1: A)** partial CO2 pressure, **B)** pH, **C)** arterial oxygen partial pressure, **D)** calculated oxygen uptake rate, **E)** perfusate lactate concentration and **F)** bile production normalized to liver weight during 6h of normothermic ex vivo liver perfusion.

**Steatotic Liver Grafts treated with 2,4 Dinitrophenol during Normothermic Machine Perfusion**
ID: 922

**Kategorie: DGAV - Transplantationschirurgie**

Joseph Gassner[1,2], Julian Michelotto[1], Simon Moosburner[1,2], Kristina Knaub[1], Andreas Birkenfeld[3], Anica Kurzbach[3], Gerald I. Shulman[4], Xian-Man Zhang[4], Markus Selzner[5], Yuki Noguchi[5], Johann Pratschke[1], Igor Maximilian Sauer[1], Nathanael Raschzok[1,2]

[1]*Charité – Universitätsmedizin Berlin, corporate member of Freie Universität Berlin and Humboldt-Universität zu Berlin, Department of Surgery, Experimental Surgery, Berlin, Deutschland*, [2]*Berlin Institute of Health at Charité – Universitätsmedizin Berlin, BIH Academy, Clinician Scientist Program, Berlin, Deutschland*, [3]*University Hospital Tübingen, Division of Endocrinology, Diabetology and Nephrology, Department of Internal Medicine, Internal Medicine IV, Tübingen, Deutschland*, [4]*Yale School of Medicine, Departments of Internal Medicine and Cellular & Molecular Physiology, Yale Diabetes Research Center, New Haven, United States*, [5]*Toronto General Hospital, University Health Network, University of Toronto, Multi Organ Transplant Program, Ajmera Family Transplant Center, Toronto, Canada*

**Background:**
The incidence of hepatic steatosis is increasing world-wide (1). As both a cause of liver cirrhosis as well as a factor for non-acceptance of potential liver grafts, steatosis hepatis increases the strain on the donor pool for liver transplantation (2, 3). Normothermic machine perfusion (NMP) in contrast to static cold storage aims to sustain the metabolism of the liver graft during the preservation period. This allows for evaluation and possibly treatment of steatotic liver grafts before transplantation (4). 2,4 Dinitrophenol (DNP) is an uncoupler of the mitochondrial ATP production, leading to increased catabolism of cellular energy storages, namely fats (5). The purpose of the study is to demonstrate that steatotic liver grafts can be defatted by DNP during NMP

**Materials and methods:**
Hepatic steatosis was induced in male Sprague Dawley rats by a 3-week high-fat diet. Steatotic liver grafts and controls from lean rats were perfused for 3 and 6 hours using a NMP system with 100, 200, 300, 400 and 500 µmol of DNP (n=6 per group). In addition, two severely steatotic, discarded human livers were treated over 12 hours with and without 200µmol DNP by NMP. Perfusate and tissue samples were used for biochemical, molecular, and histological analyses.

**Results:**
Both tissue triglyceride content ($p<0.0001$) and Oil-red-O staining showed a time- and dose-dependent effect on hepatic steatosis by DNP. Tissue triglyceride levels (81.2 vs. 56.01mg/g; $p=0.021$) indicated optimal dose-response after 6 hours of perfusion with 200µmol of DNP (Figure 1), with sustained bile production (622.5 vs. 488.8mg at 6h; $p=0.93$) and low transaminase secretion (127U/L vs. 127.5U/L ALT at 6h; $p>0.999$) even when compared to livers from lean donors. DNP accumulation was minimal in liver tissue. Liver grafts treated with DNP showed increased lactate clearance (34.5 vs. 8.3mg/dL; $p=0.001$) Western blot analyses showed significantly increased levels of mitochondrial fission factor and a positive effect on posphorylated Akt kinase (p-Akt) and the Akt substrate caspase 9 levels. In contrast to the control, the human liver treated with DNP cleared lactate after 1 hour, maintained perfusate pH without correction after 9 hours and resumed producing bile after 11 hours of NMP.

**Conclusion:**
Treatment of steatotic liver grafts during NMP is able to reduce triglyceride content and improve lactate clearance without increased transaminase release both in human and rat experiments. It therefor appears to have the potential of future clinical application for conditioning of severely steatotic liver grafts and make them available for transplantation.

**References:**

1. Maurice J, Manousou P: Non-alcoholic fatty liver disease. Clinical medicine (London, England) 2018; 18: 245-50.
2. Tacke F, Kroy DC, Barreiros AP, Neumann UP: Liver transplantation in Germany. Liver Transpl 2016; 22: 1136-42.
3. Moosburner S, Raschzok N, Schleicher C, et al.: [Declined Liver Grafts - Analysis of the German Donor Population from 2010 to 2018]. Zeitschrift für Gastroenterologie 2020; 58: 945-54.
4. Nasralla D, Coussios CC, Mergental H, et al.: A randomized trial of normothermic preservation in liver transplantation. Nature 2018; 557: 50-6.
5. Perry RJ, Shulman GI: Treating fatty liver and insulin resistance. Aging (Albany NY) 2013; 5: 791-2.

**Graphics:**

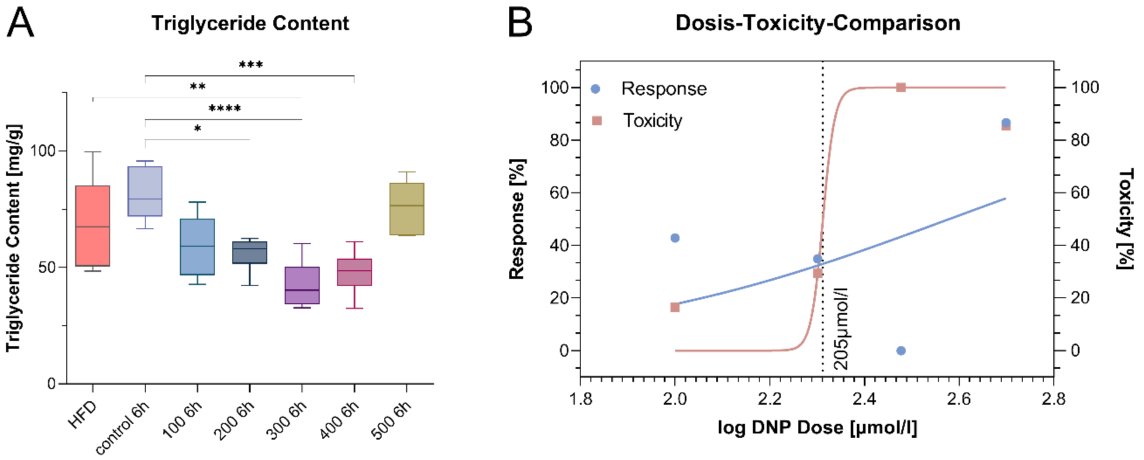

**Figure 2: A)** Triglyceride content in steatotic liver grafts without perfusion (HFD) 6h NMP without 2,4-Dinitrophenol (DNP) and with increasing DNP concentrations. **B)** Dose-Toxicity calculation according to ALT release and reduction of triglyceride content

**Intraoperative fluorescence lymphography to avoid lymphoceles in kidney transplantation**
ID: 932

**Kategorie: DGAV - Transplantationschirurgie**

Michael Thomas[1], Marielle Hummels[1], Hans Fuchs[1], Rabi Datta[1], Roger Wahba[1], Christiane Bruns[1], Christine Kurschat[2], Dirk Stippel[1]

[1]*Uniklinik Köln, Klinik für Allgemeine-, Viszeral-, Tumor- und Transplantationschiururgie, Köln, Deutschland*, [2]*Uniklinik Köln, Klinik für Nephrologie, Köln, Deutschland*

**Background:**
The occurrence of postoperative lymphoceles is one of the most common complications after kidney transplantation. Their incidence is listed in the literature between 0.6% and 51%. In addition to asymptomatic courses, lymphoceles can cause serious complications that can result in organ loss of the transplanted organ.

**Materials and methods:**
Intraoperative fluorescence lymphography was performed in 20 consecutive living-donor kidney transplantions. For this purpose, 2 ml indocyanine green (ICG) (2.5 mg/ml) was applied subcutaneously into the trigonum femorale lateral to the femoral artery. During kidney transplantation the integrity of the lymphatics was documented at 3 different time points (before exposure of the iliac vessels (T0), after exposure of the iliac vessels (T1) after kidney transplantation (T2)) using a fluorescence camera (Spy-Phi/Stryker). Postoperatively, weekly ultrasound investigations were carried out to document perirenal fluid and an MRI of the Tx kidney was performed 6 months after transplantation.

**Results:**
In all 20 consecutively performed living-donor kidney transplants, the retroperitoneal lymphatic vessels could be identified and spared during preparation by ICG application. In the sonographic controls carried out postoperatively, none of the analyzed patients showed perirenal fluid. The MRI examination of the pelvis carried out after 6 months also showed no perirenal fluid collection in any of the analyzed patients. In none of the patients an undesirable side effect from subcutaneous injection of ICG was observed.

**Conclusion:**
Intraoperative use of fluorescence lymphography is an effective and technically feasible method to protect lymphatic structures during kidney transplantation.
